# Bias in Odds Ratios From Logistic Regression Methods With Sparse Data Sets

**DOI:** 10.2188/jea.JE20210089

**Published:** 2023-06-05

**Authors:** Masahiko Gosho, Tomohiro Ohigashi, Kengo Nagashima, Yuri Ito, Kazushi Maruo

**Affiliations:** 1 Department of Biostatistics, Faculty of Medicine, University of Tsukuba, Ibaraki, Japan; 2 Graduate School of Comprehensive Human Sciences, University of Tsukuba, Ibaraki, Japan; 3 Department of Biostatistics, Tsukuba Clinical Research & Development Organization, University of Tsukuba, Ibaraki, Japan; 4 Research Center for Medical and Health Data Science, The Institute of Statistical Mathematics, Tokyo, Japan; 5 Department of Medical Statistics, Research & Development Center, Osaka Medical College, Osaka, Japan

**Keywords:** Bayesian methods, exact logistic regression method, Firth’s penalization, *ɡ*-prior

## Abstract

**Background:**

Logistic regression models are widely used to evaluate the association between a binary outcome and a set of covariates. However, when there are few study participants at the outcome and covariate levels, the models lead to bias of the odds ratio (OR) estimated using the maximum likelihood (ML) method. This bias is known as sparse data bias, and the estimated OR can yield impossibly large values because of data sparsity. However, this bias has been ignored in most epidemiological studies.

**Methods:**

We review several methods for reducing sparse data bias in logistic regression. The primary aim is to evaluate the Bayesian methods in comparison with the classical methods, such as the ML, Firth’s, and exact methods using a simulation study. We also apply these methods to a real data set.

**Results:**

Our simulation results indicate that the bias of the OR from the ML, Firth’s, and exact methods is considerable. Furthermore, the Bayesian methods with hyper-*ɡ* prior modeling of the prior covariance matrix for regression coefficients reduced the bias under the null hypothesis, whereas the Bayesian methods with log *F*-type priors reduced the bias under the alternative hypothesis.

**Conclusion:**

The Bayesian methods using log *F*-type priors and hyper-*ɡ* prior are superior to the ML, Firth’s, and exact methods when fitting logistic models to sparse data sets. The choice of a preferable method depends on the null and alternative hypothesis. Sensitivity analysis is important to understand the robustness of the results in sparse data analysis.

## INTRODUCTION

Logistic regression models are most frequently used to assess the relationship between a binary outcome and a set of covariates, and the relationship can be interpreted using an odds ratio (OR) with a 95% confidence interval (CI). Although the OR is usually estimated using the maximum likelihood (ML) method, the ML estimate of the OR can have considerable upward or downward bias^1^ when there are few study participants at combinations of the outcome and covariate levels, or when there is an unbalanced structure of covariates. This bias is known as small sample bias or sparse data bias.^2^ Note that this issue occurs when the event rate is low relative to the sample size. Accordingly, the issue can occur even in studies with a large sample size.

In their clinical study, Masuda et al (2017) determined significant risk factors of thromboembolic events among patients with atrial tachyarrhythmias using univariate logistic regression analyses.^3^ Their results suggested that vascular disease is a significant risk factor for thromboembolic events with an OR of 7.83 (95% CI, 1.19–52.53). However, Ayubi et al (2017) sent a letter to the editor of the journal in which this study was published, expressing their disagreement with this analysis and interpretation.^4^ They highlighted the issue of possible sparse data bias when using an OR estimated using logistic regression analyses based on the ML method; the combination of vascular disease and thromboembolic events were rare, as there were only five participants with vascular disease, two of whom had thromboembolic events (Table 1). This is a common example of sparse data sets.

**Table 1.** Contingency table between vascular disease and thromboembolic event in Masuda et al (2017)

		Thromboembolic event		
Vascular disease		No	Yes	Total
	No	141	12	153
	Yes	3	2	5
	Total	144	14	158

Unadjusted odds ratio 7.83 (95% confidence interval, 1.19–52.53).

The issue is worse in multivariate regression analyses than in univariate regression analyses.^2^ The multivariate analyses are often performed to assess the OR for a factor of interest after a regression adjustment for the confounding variables; however, sparse data bias of the OR can be larger than the bias removed by the adjustment.^2^ The estimated ORs could yield impossibly large values because of data sparsity. This estimate inflation might be apparent in light of background information, but is rarely noted or accounted for in epidemiological studies.^2^ As another example, a cohort study provided the unadjusted OR of 21.0 (95% CI, 1.1–120.0) for the association of hydramnios with neonatal death, and the adjusted OR of 60.0 (95% CI, 5.7–365.0) in a logistic model involving 14 covariates and 17 events.^5^ The hydramnios association is implausible and provides a warning that the increase in estimates after an adjustment is likely to reflect sparse data bias, rather than the removal of confounding. This case study is described further in the Application section below.

To address sparse data bias, several methods have been proposed. These include: (1) Firth’s penalizations^6^; (2) the exact logistic regression method^7^^,^^8^; and (3) the Bayesian approaches. Particularly, several approaches have been proposed in Bayesian inference, as the specification of the prior distribution for regression coefficients is wide-ranging and controversial. The log *F*-type priors are currently a popular method for solving the sparse data problem in applications.^2^^,^^9^ In fact, Ayubi et al (2017) applied this method to the previous example for analyzing the association between thromboembolic events and vascular disease.^4^ Furthermore, Held et al (2015)^10^ utilized Zellner’s *ɡ*-prior,^11^ with the prior covariance matrix proportional to the covariance matrix of the regression coefficients estimated using the ML method. They proposed Bayesian procedures using a suitable hyper-*ɡ* prior in logistic regression. These methods are illustrated in the Methods section below.

Some researchers have provided insightful reviews and practices of several methods to handle sparse data bias,^2^^,^^12^^,^^13^ although they did not quantitatively compare the magnitude of the bias of the OR estimate using bias-correction methods. By contrast, Heinze and colleagues have compared the performance of these methods for logistic regression with sparse data quantitively, and have shown that Firth’s penalized method outperformed the standard ML method and the exact logistic regression method in terms of reducing the bias when estimating the OR.^14^^–^^16^ However, Bayesian approaches have not been sufficiently compared with Firth’s method and the exact method, which are commonly used in applications with sparse data. The aim of this study is to evaluate the performance of the Bayesian approaches in comparison with that of the frequentist approaches, such as the ML, Firth’s, and exact methods, through simulation studies.

## METHODS

In this section, we briefly review several frequentist and Bayesian approaches for addressing sparse data bias.

### Frequentist approaches

#### Logistic regression models and the ML estimate

Let *Y_i_* ( i=1,…,n ) be a binary outcome for the *i*th participant that follows a Bernoulli distribution with the binominal probability *π_i_* = Pr(*Y_i_* = 1). The logistic regression model is defined as:

$$\log\frac{\pi_{i}}{1-\pi_{i}}=\beta^{\text{T}}x_{i}$$

where $\beta^{\text{T}}=(\beta_{0},\beta_{1},\ldots ,\beta_{j},\ldots ,\beta_{k})$ is the (*k* + 1)-vector of regression coefficients, and $x_{i}=(1,x_{i1},\ldots ,x_{ik}{)}^{\text{T}}$ is the covariate (*k* + 1)-vector with *k*-risk factors for the *i*th participant. Ordinarily, β is estimated using the ML method; that is, the ML estimate $\hat{\beta }$ is calculated by maximizing the log-likelihood l(β) . In other words, $\hat{\beta }$ is a solution of the score equation

$$U(\beta_{j})=\frac{\partial l(\beta )}{\partial \beta_{j}}=\sum_{i=1}^{n}(Y_{i}-\pi_{i})x_{ij}=0\;(j=0,\ldots ,k),$$

where $\pi_{i}=\{1+\exp(-\beta^{\text{T}}x_{i}){\}}^{-1}$ . The ML estimate $\hat{\beta }$ would be biased upward or downward when the number of events is small and the number of participants is considerably unbalanced among covariate levels.

The CIs of β are commonly obtained through normal approximation (ie, the Wald method) or profile likelihood. The Wald-type CI performs poorly compared with the profile likelihood-type CI in sparse data sets because of the failure of the normal approximation that is included in its computation.^15^ Thus, in this study, we use the profile likelihood-type CI. The 100 (1 − *α*) % CI is the set of all values *β_j_* ( j=0,1,…,k ) where a generalized likelihood ratio test of the null hypothesis H_0_: *β_j_* = *β_j_*_0_ would not be rejected at the *α* significance level.^17^

#### Firth’s method

To reduce the bias of the ML estimator, Firth (1993) proposed a penalty term $\frac{1}{2}\text{trace}\left\{\frac{I(\beta {)}^{-1}\partial I(\beta )}{\partial \beta_{j}}\right\}$ in score function U(*β_j_*) = 0, where I(β) is the Fisher information matrix for β .^6^ The modified score equation is given by

$$U_{\text{M}}(\beta_{j})=U(\beta_{j})+\frac{1}{2}\text{trace}\left\{\frac{I(\beta {)}^{-1}\partial I(\beta )}{\partial \beta_{j}}\right\}=0\;(j=0,\ldots ,k).$$

The corresponding penalized log-likelihood function for the above-modified score function is

$$l^{*}(\beta )=l(\beta )+\frac{1}{2}\log|I(\beta )|.$$ (1)

The penalty term in (1) is known as a Jeffreys invariant prior, and penalization is mathematically identical to what is known as Bayesian analysis. Firth’s method removes the first-order term in the asymptotic bias expansion of ML estimate $\hat{\beta }$ . The ML estimate and Firth’s estimate will virtually coincide in large data sets because the penalty term in (1) is asymptotically negligible.^18^

Firth’s method is particularly attractive in contrast to *post hoc* bias corrections.^19^ In addition, it always leads to finite estimates of the OR and its CI. Although the CI can be obtained using the Wald or profile likelihood methods defined above, the profile likelihood CI is statistically superior to the Wald CI with sparse data.^15^ Further, Firth’s method can be used not only for logistic regression models but also for generalized linear models (GLMs) and Cox regression models.^6^^,^^16^ Conversely, Firth’s method does not minimize the mean squared error of the estimated OR, and it can produce implausible estimates in applications to sparse data.^12^ Firth’s estimate may lead to more bias than that in the ML estimate when the true OR moves away from 1.^20^ Although the Firth penalty corresponds with the Jeffreys prior in the Bayesian analysis framework, the Jeffreys prior underlying the Firth penalty can lead to artifact estimates lying outside the range of the prior median and the ML estimation.^12^ That is, Firth’s estimate is sometimes larger than the ML estimate, and Firth’s method may not sufficiently reduce the bias of the estimated OR.

#### Exact method

The exact method is useful for calculating parameter estimates and standard errors when the sample size is very small and/or is unbalanced between covariate levels. The method constructs a statistical distribution that can be completely determined. Mehta & Patel (1995) introduced the exact conditional logistic regression using permutational distributions of sufficient statistics.^8^ The estimate and inference of *β_j_* are based on the exact null distribution of the sufficient statistic $T_{j}=\sum_{i=1}^{n}y_{i}x_{ij}$ of *β_j_*, conditioning on the sufficient statistics *T_J_* ( J={0,…,k},j∉J ) corresponding to all other regression parameters, except for *β_j_*. The estimate of *β_j_* is given by maximizing the conditional likelihood:

$$\begin{array}{rl} \mathrm{Pr}(T_{j}=t_{j}|\beta_{j},T_{J}=t_{J}) & \equiv {\mathrm{Pr}}_{j}(T_{j}=t_{j}|\beta_{j})\\ & =L_{j}(\beta_{j}|T_{j}=t_{j},T_{J}=t_{J})\\ & =\frac{\exp(\beta_{j}t_{j})}{\sum_{\Omega_{j}}\exp\left(\beta_{j}\sum_{i=1}^{n}y_{i}^{*}x_{ij}\right)} \end{array}$$

with Ω*_j_* denoting the set of all $\sum_{i=1}^{n}y_{i}^{*}x_{ij}$ from permutations $y^{*}=(y_{1}^{*},\ldots ,y_{n}^{*})$ of the observed outcome vector $y=(y_{1},\ldots ,y_{n})$ .^16^ Here, *t_j_* and *t_J_* are the observed values of *T_j_* and *T_J_*, respectively. If *t_j_* is at the maximum (minimum) of its distribution, then the estimate of *β_j_* is not finite. In this case, the estimate is replaced by a median unbiased estimate, which is defined as the value of *β_j_* that satisfies *L_j_*(*β_j_*|*T_j_* = *t_j_*, *T_J_* = *t_J_*) = 1/2.

Using the exact conditional distribution of *T_j_*, 100 (1 − *α*)% CI is estimated by computing the values *β_j_*_−_ and *β_j_*_+_ that satisfy $\mathrm{Pr}(T_{j}\geq t_{j}|\beta_{j-})=\frac{\alpha }{2}$ and $\mathrm{Pr}(T_{j}\leq t_{j}|\beta_{j+})=\frac{\alpha }{2}$ , respectively. This interval estimation is generally conservative when the sample size is small and the data are sparse. To address this issue, the mid *P*-type CI is sometimes used and defined by the values *β_j_*_−_ and *β_j_*_+_ that satisfy $\mathrm{Pr}(T_{j}\geq t_{j}|\beta_{j-})+\frac{\mathrm{Pr}(T_{j}=t_{j}|\beta_{j-})}{2}=\frac{\alpha }{2}$ and $\mathrm{Pr}(T_{j}\leq t_{j}|\beta_{j+})+\frac{\mathrm{Pr}(T_{j}=t_{j}|\beta_{j+})}{2}=\frac{\alpha }{2}$ , respectively.^21^

The advantages of the exact method are that it allows for exact inference and yields an unbiased finite median estimate, even in the case of complete separation. However, the exact method may not sufficiently reduce the bias of the estimated OR, and loss of efficiency, which is an increasing variance of the parameter estimate, and may result from conditioning on parameters that are not subject to inference in multivariate logistic regression analysis.^16^

### Bayesian approaches

Bayesian statistical inference is conducted using the posterior distribution of the parameter of interest. The posterior distribution is proportional to two components: a likelihood function incorporating information about the parameter based on observed data and prior distribution containing known information about the parameter. To summarize the posterior information, we use the posterior median and the equal-tailed 95% posterior credible interval for log OR in this paper. Thus, the posterior median will be contrasted with the ML estimate for log OR in this paper. In addition, the 95% posterior credible interval has a natural interpretation not shared by the 95% CI in frequentist statistics.^22^ The 95% credible interval contains the 95% most plausible parameter values based on the observed data and prior information, while the 95% CI does or does not contain the true value for a particular data set.^22^ For more information about the interpretation of the two intervals, see Lesaffre and Lawson (2012).^22^

Although there are various Bayesian methods,^12^^,^^23^^–^^26^ we introduce the normal prior as a non-informative prior, the hyper-*ɡ* prior, and the log *F*-type prior approaches. The hyper-*ɡ* prior is discussed for the posterior estimation in the small number of events in Held & Sauter (2017).^27^ Use of the log *F*-type prior is recommended for analyzing the small-sample data set in Ayubi et al (2017)^4^ introduced above.

#### The normal prior and hyper-ɡ prior

In the general framework of Bayesian inference on logistic regression analyses, we often use a multivariate normal prior distribution for regression coefficients because the estimated regression coefficients asymptotically follow the multivariate normal distribution. If there is no evidence for the coefficients, we would assume that β is a priori normally distributed with mean **0** and covariance matrix Σ that is a diagonal matrix with all variances equal to a large value (eg, 100).

Zellner’s *ɡ*-prior for Gaussian linear models,^11^ with a prior covariance matrix proportional to the covariance matrix **M** of the ML estimate of the regression coefficients, is a commonly used default prior. Zellner (1986) introduced a multivariate normal prior with mean **0** and covariance matrix *ɡ***M**, using hyper-parameter *ɡ*.^11^ This is a natural approach incorporating prior correlations between regression coefficients and automatically adjusts for different variances of the covariates.^27^ Sabanés Bové & Held (2011) extended the hyper-parameter *ɡ* to GLMs, including logistic regression models.^29^

Liang et al (2008) discussed a Bayesian approach with a hyper-*ɡ* prior with prior density on *ɡ*^30^:

$$f(g)=\frac{a-2}{2}(1+g{)}^{-\frac{a}{2}},\;g>0,$$

which is a proper distribution for *a* > 2. This prior includes priors used by Strawderman (1971)^31^ to provide improved mean square risk over ordinary ML estimates in the normal means problem. In addition, this prior distribution is a special case of a class of prior distributions proposed by Cui & George (2008)^32^ and induces a beta distribution for the shrinkage factor *ɡ*/(*ɡ* + 1) ∼ Beta(1, *a*/2 − 1). Of particular interest is the case *a* = 4, where the prior on the shrinkage factor is standard uniform, and thus, the prior median of *ɡ* is 1. The cumulative density function of *ɡ* has a simple analytic form, *F*(*ɡ*) = *ɡ*/(*ɡ* + 1). The prior probabilities of interest can easily be calculated. For example, according to Held and Suter (2017), Pr(1/2 ≤ *ɡ* ≤ 2) = 1/3 or Pr(1/19 ≤ *ɡ* ≤ 19) = 0.9.^27^ The hyper-parameter *ɡ* has an infinite expectation and dispersion.^27^ Choice 2 < *a* ≤ 4 may be reasonable.^30^ *a* = 4 is also recommended.^27^ Thus, we set *a* = 4 in this study.

In this study, we assign the hyper-*ɡ* prior N(ν,gΣ) to β . The prior mean parameter is set to ν=0 , assuming no evidence for the location of β , and avoiding arbitrary choices. We also set the prior covariance matrix $\Sigma =\text{diag}(\frac{1}{2})$ based on Held & Sauter (2017).^27^

#### Log F-type priors

Greenland & Mansournia (2015) proposed data augmentation methods with log *F*-type priors.^12^ They considered an intercept-only logistic model for *Y_i_* (ie, assume $\beta =\beta_{0}=\beta$ ). In this case, $I(\beta )=I(\beta )=n\exp(\beta )/\{1+\exp(\beta ){\}}^{2}$ and log|*I*(*β*)|^−1^ = 2 log{1 + exp(*β*)} − *β* − log(*n*). Apart from the constant term, using log|*I*(*β*)|^−^*^m^* = 2*m* log{1 + exp(*β*)} − *mβ* − log(*n*), indexed by *m* ≥ 0, the log-likelihood $l^{*}(\beta )$ in (1) is expressed as

$$l^{*}(\beta )=l(\beta )+m\beta /2-m\log\{1+\exp(\beta )\}.$$ (2)

The antilog of the augmentation term *mβ*/2 − *m* log{1 + exp(*β*)} in (2) is proportional to the log *F*(*m*, *m*) density for *β*, which is in the conjugate family for binomial logistic regression; it is also the prior distribution for *β* induced by a conjugate Beta(*m*/2, *m*/2) density for *π*.^12^

Practically, to shrink overestimated OR estimates in sparse data analyses, one may begin by specifying an interval that encodes the idea that the true OR is not overestimated. For example, assume that the OR falls between 1/40 and 40. The interval of 1/40 to 40 would become a conservative 95% prior interval for the OR, where “conservative” means that the 95% is a minimum certainty, and “prior” means the limits were derived from background information, rather than the study data.^5^ This approach assumes that the OR has an *F*(*m*, *m*) prior distribution with degrees of freedom *m*. The 95% prior interval for the OR corresponds to the *F*(*m*, *m*) prior and becomes narrower as *m* increases.

In the relationship between *F*(*m*, *m*) priors and data augmentation, Greenland & Mansournia (2015) showed that the regression coefficient β with log *F*(0, 0) prior is equivalent to the ML estimate, and log *F*(1, 1) includes the Jeffreys prior in an intercept-only model.^12^ Log *F*(1, 1) and log *F*(2, 2) priors correspond to the 95% prior intervals for the OR of 1/648 to 648 and 1/39 to 39, respectively. In addition, based on Brown et al (2002), who introduced the log *F* distributions, the variances of β for log *F*(1, 1) and *F*(2, 2) priors are approximately 9.87 and 3.29, respectively.^39^ In this study, we focus on log *F*(1, 1) and log *F*(2, 2) priors because of computational simplicity and their popularity in medical research.^2^^,^^28^

To apply the data augmentation method, the prior intervals are translated into simple prior data (pseudo data) that are added to the actual data. Data augmentation treats them as distinct data records that would reproduce the prior interval for each variable as CIs. Appending this prior data set to the actual data incorporates the information contained in the prior intervals, pulling extreme estimates into a more reasonable range.^2^ Detailed procedures are shown in Sullivan & Greenland (2013).^5^

## SIMULATION STUDY

In simulations using sparse data sets, we compared the performance of the ML method (under ML), Firth’s method (FIR), the exact method (EX), mid-*P* type method (MDP), and Bayesian methods with normal prior *N*(0, 100) (NP), with the hyper-*ɡ* prior (HG), with log *F*(1, 1) prior (F1), and with log *F*(2, 2) prior (F2), introduced in the methods section. Simulation studies are performed using SAS 9.4 (SAS Institute, Cary, NC, USA).

The total sample size, *n*, is set to 100, 300, 1,000, and 3,000. The expected number of events, *n_e_*, is 5, 10, and 20. Data generation is repeated 1,000 times for each setting. The required number of replications was 957 based on the 95% CI for the difference in the log OR between two estimation methods to be ±log(1.1) under the null hypothesis when the variance of the log OR was maximized within our simulation settings and the correlation coefficient between the methods was assumed to be 0.5.

We present six covariate scenarios. The covariate is the exposure *x*_1_ of interest in all the scenarios. *x*_1_ follows a Bernoulli distribution with probability, $\pi_{x_{1}}=0.05$ , 0.1 or 0.2. The levels of exposure *x*_1_ are unbalanced. In scenario 1, the covariate is the only exposure *x*_1_. In scenario 2, the covariates are exposures *x*_1_ and *x*_2_. The covariates *x*_1_ and *x*_2_ independently follow a Bernoulli distribution with probability $\pi_{x_{1}}$ and 0.5, respectively. In scenario 3, the covariates are *x*_1_ and *x*_2_, and these are correlated. *x*_1_ follows a Bernoulli distribution with probability $(x_{2}+\frac{1}{2})\pi_{x_{1}}$ . *x*_2_ follows a Bernoulli distribution with probability 0.5. In scenario 4, there are four covariates. The distribution of *x*_1_ is the same as in scenario 3. *x*_2_, *x*_3_, and *x*_4_ independently follow a Bernoulli distribution with probability 0.5, 0.3, and 0.1, respectively. There are eight covariates in scenario 5. The distribution of *x*_1_ is the same as in scenario 3. *x*_2_, *x*_3_, *x*_4_, *x*_5_, *x*_6_, and *x*_7_ independently follow a Bernoulli distribution with probability 0.5, 0.3, 0.1, 0.5, 0.3, and 0.1, respectively. *x*_8_ follows a trinomial distribution with probability 0.8, 0.1, and 0.1. In scenario 6, the simulation data were generated assuming a case study illustrated below. The eight covariates are generated as: *x*_1_ ∼ *Bin*(1, 0.0033), *x*_2_ ∼ *Bin*(1, 0.1858), *x*_3_ ∼ *Bin*(1, 0.0100), *x*_4_ ∼ *Bin*(1, 0.0157), *x*_5_ ∼ *Bin*(1, 0.0391), *x*_6_ ∼ *Multi*(0.8021, 0.1715, 0.0264), *x*_7_ ∼ *Multi*(0.9850, 0.0090, 0.0060), *x*_8_ ∼ *Multi*(0.8269, 0.0234, 0.0498, 0.0999). Here, *Multi* is the multinomial distribution. Based on Sullivan & Greenland (2013),^5^ all the covariates are included in the multivariate model as quantitative variables.

Binary outcome *y* is generated from a Bernoulli distribution with probability *π_y_*, where $\pi_{y}=\frac{\exp(\beta^{\text{T}}x_{i})}{\left[1+\exp(\beta^{\text{T}}x_{i})\right]}$ . The simulated number of events follows a binominal distribution *Bin*(*n*, *π_y_*). The true OR for *x*_1_ was set to 1 (ie, *β*_1_ = log 1), 4 (ie, *β*_1_ = log 4), and 16 (ie, *β*_1_ = log 16). The first is under the null hypothesis, and the second and third are under the alternative hypothesis. The OR for *x*_2_ to *x*_8_ was set to 2 (ie, *β*_2_ = … = *β*_8_ = log 2) in scenarios 2 to 5. *β*_0_ is specified based on the expected number of events, true OR(s), and the binominal probability of covariate(s). In scenario 6, we set *β*_0_ = −6.40, *β*_2_ = 0.57, *β*_3_ = 0.96, *β*_4_ = 2.09, *β*_5_ = 1.24, *β*_6_ = 1.52, *β*_7_ = 0.99, and *β*_8_ = −0.64 based on the case study below.

We applied the logistic regression methods introduced in the methods section to the generated data sets. We assessed the OR for *x*_1_, defined as the exponential of the estimated *β*_1_, or the exponential of the posterior median of *β*_1_ for NP and HG. The average OR for *x*_1_ is defined as the exponential average estimated *β*_1_, or the exponential average of the posterior median of *β*_1_. We calculated the bias for the log OR, defined as the estimated *β*_1_ or the posterior median of *β*_1_ minus the logarithm of the true OR. We also assessed the coverage probability (CP) of the two-sided 95% CIs for *β*_1_, which was defined as the percentage of 95% profile likelihood CIs that contained the true value of *β*_1_. The CP for the NP and HG was defined as equal-tailed 95% posterior credible intervals for *β*_1_ that contained the true value of *β*_1_. The OR is always the same for the exact and mid-*P* type methods. The convergence success was defined such that the OR can be estimated in SAS default settings, and the estimated OR fell within the range between 0.001 and 999. The proportions of successful convergence were 70% for the ML; >99% for FIR, EX, and MDP; 91% for NP; and >99% for HG, F1, and F2 in all the scenarios.

The supplementary materials contain all the results from the simulation scenarios. Although FIR and EX generally produce estimates regardless of the “separation,” which is defined as a situation in which a single independent variable or a linear combination of covariates perfectly predicts the binary outcome,^15^ we restrict our discussion to cases where there are ML estimates to ensure the comparability of the ML method with the other methods, since the ML method is routinely used for logistic regression analysis on sparse data. As reference, we provide the results for all cases without the restriction in the supplementary materials. There was no significant difference in the simulation results between the cases with and without the restriction when $\pi_{x_{1}}=0.2$ and *n_e_* ≥ 10 or $\pi_{x_{1}}=0.1$ and *n_e_* = 20. Conversely, the ORs without the restriction were generally smaller than those with the restriction in the other settings for $\pi_{x_{1}}$ and *n_e_* (see eFigure 48 through eFigure 63).

**Figure 1.**
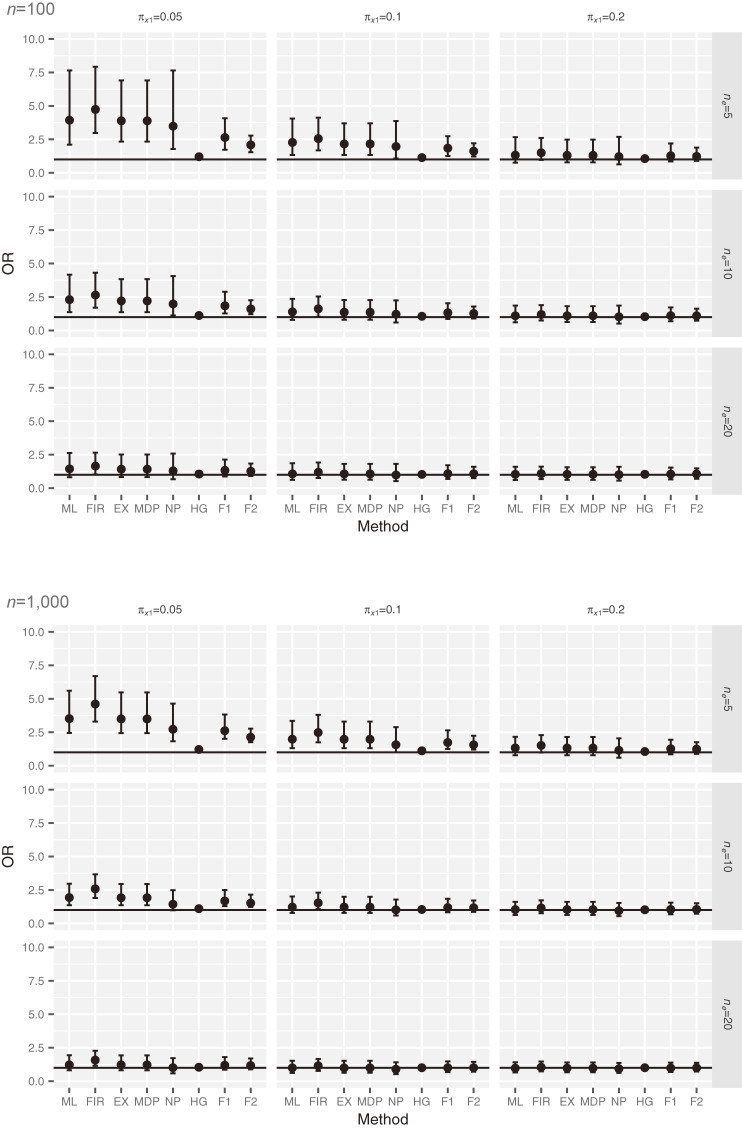
Distribution of simulated OR under the null hypothesis in scenario 4. The square represents the median and the error bar represents quartiles 1 and 3. The solid horizontal line is the true OR value. EX, exact method; F1, Bayesian data augmentation with log *F*(1, 1); F2, Bayesian data augmentation with log *F*(2, 2); FIR, Firth’s method; HG, Bayesian method with hyper-*ɡ* prior; MDP, mid *P*-type exact method; ML, ML method; NP, Bayesian method with *N*(0, 100) prior.

Figure 1 presents the distribution of the simulated OR under the null hypothesis with *n* = 100 and 1,000 in scenario 4. The ORs for ML, FIR, EX, and NP are overestimated when $\pi_{x_{1}}$ and *n_e_* are small. For example, when *n* = 100, $\pi_{x_{1}}=0.05$ , and *n_e_* = 5, the average ORs for ML, FIR, EX, and NP are 3.91, 4.90, 4.09, and 2.95, respectively. Additionally, the biases of the OR for F1, F2, and HG are smaller than those using the classical methods (ML, FIR, and EX), and the average ORs for HG, F1, and F2 are 1.28, 2.65, and 2.08, when *n* = 100, $\pi_{x_{1}}=0.05$ , and *n_e_* = 5.

**Figure 2.**
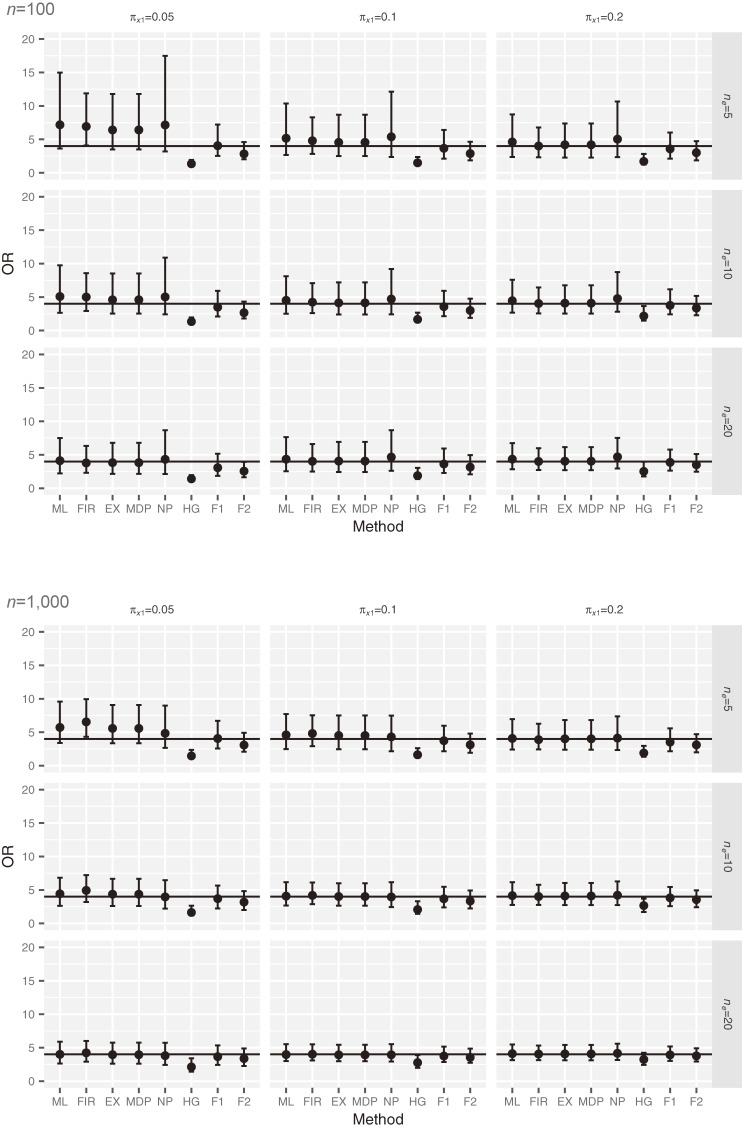
Distribution of simulated OR under the alternative hypothesis (true OR = 4) in scenario 4. The square represents the median and the error bar represents quartiles 1 and 3. The solid horizontal line is the true OR value. EX, exact method; F1, Bayesian data augmentation with log *F*(1, 1); F2, Bayesian data augmentation with log *F*(2, 2); FIR, Firth’s method; HG, Bayesian method with hyper-*ɡ* prior; MDP, mid *P*-type exact method; ML, ML method; NP, Bayesian method with *N*(0, 100) prior.

Figure 2 presents the distribution of the OR under the alternative hypothesis (true OR = 4) with *n* = 100 and 1,000 in scenario 4. The ORs for ML, FIR, EX, and NP are overestimated when $\pi_{x_{1}}$ and *n_e_* are small, as under the null hypothesis. For example, when *n* = 100, $\pi_{x_{1}}=0.05$ , and *n_e_* = 5, the average ORs for ML, FIR, EX, and NP are 7.38, 7.04, 6.58, and 7.11, respectively. By contrast, when *n* = 100, $\pi_{x_{1}}=0.05$ , and *n_e_* = 5, the ORs for HG and F2 are 1.70 and 3.13, respectively, and are lower than the true value of 4. The OR for F1 is close to 4 even when $\pi_{x_{1}}$ and *n_e_* are small. For example, when *n* = 100, $\pi_{x_{1}}=0.05$ , and *n_e_* = 5, the average OR for F1 is 4.35.

**Figure 3.**
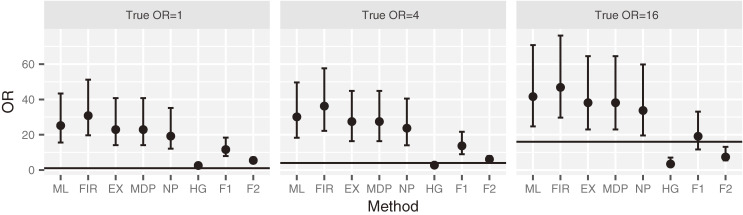
Distribution of simulated OR in scenario 6 based on the case study. The square represents the median and the error bar represents quartiles 1 and 3. EX, exact method; F1, Bayesian data augmentation with log *F*(1, 1); F2, Bayesian data augmentation with log *F*(2, 2); FIR, Firth’s method; HG, Bayesian method with hyper-*ɡ* prior; MDP, mid *P*-type exact method; ML, ML method; NP, Bayesian method with *N*(0, 100) prior.

Figure 3 presents the distribution of the OR in scenario 6 based on the case study. When the true OR is 1, the average ORs for ML, FIR, EX, NP, HG, F1, and F2 are 26, 32, 24, 20, 2.8, 12, and 5.5, respectively. The HG has the best performance in terms of bias of OR. When the true OR is 4, the average ORs for ML, FIR, EX, NP, HG, F1, and F2 are 31, 37, 28, 25, 3.2, 14, and 6.4, respectively. The ORs for HG and F2 are relatively close to the true value of 4. When the true OR is 16, the average ORs for ML, FIR, EX, NP, HG, F1, and F2 are 44, 49, 39, 36, 4.5, 21, and 9.3, respectively. The OR for F1 is the closest to the true value among the methods.

**Figure 4.**
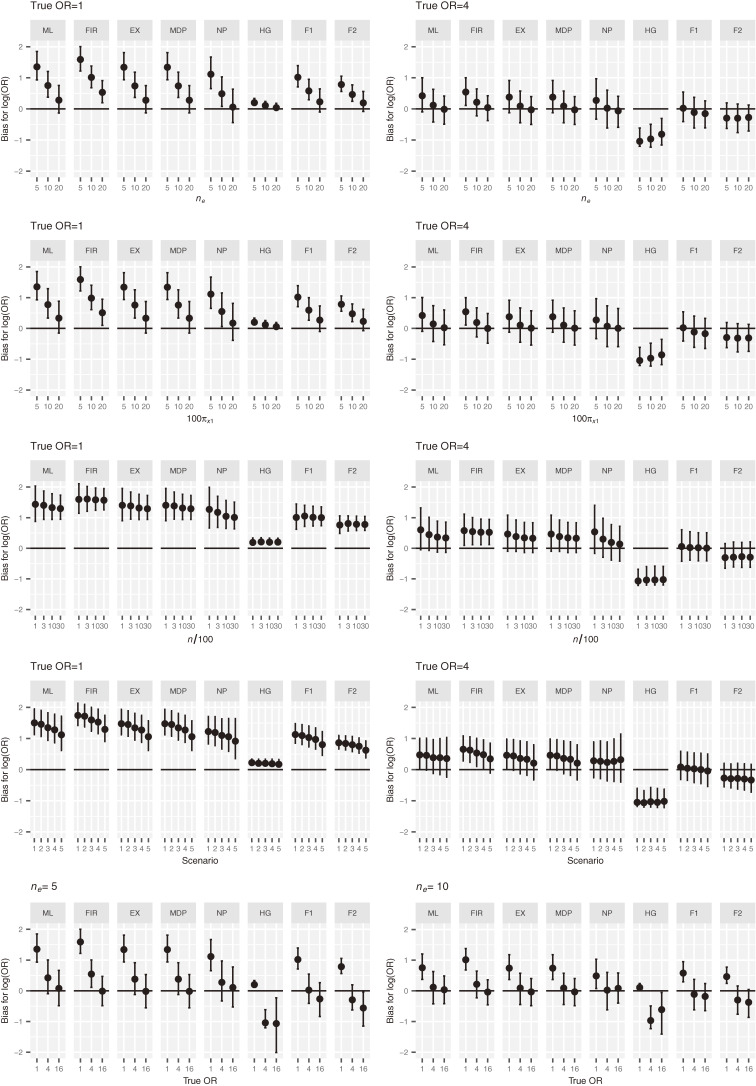
Distribution of bias for log OR under the various conditions. The square represents the median and the error bar represents quartiles 1 and 3. EX, exact method; F1, Bayesian data augmentation with log *F*(1, 1); F2, Bayesian data augmentation with log *F*(2, 2); FIR, Firth’s method; HG, Bayesian method with hyper-*ɡ* prior; MDP, mid *P*-type exact method; ML, ML method; NP, Bayesian method with *N*(0, 100) prior.

Figure 4 presents the distribution of the bias of the log OR when changing the number of events *n_e_*, the imbalance in the levels $\pi_{x_{1}}$ , the total sample size *n*, the scenario, and the true OR value. The number of covariates for scenarios 1, 2, 3, 4, and 5 is 1, 2, 2, 4, and 8, respectively. There is no correlation between the two covariates in scenario 2, and there is a correlation between the two covariates in scenario 3. The magnitude of biases for ML, FIR, EX, and NP decreases as the number of events increases and as the imbalance in the levels of covariate *x*_1_ reduces. The biases are not strongly dependent on the total sample size for all the methods. Contrary to expectations, the magnitude of biases for all the methods, except for HG, decreases as the number of covariates increases under the null hypothesis. This phenomenon would be dependent on the distributions of other covariates as well as the exposure *x*_1_ of interest. The bias of ML, FIR, and EX without correlation between the two covariates in scenario 2 was larger than the bias with correlation in scenario 3. The bias of all the methods, except HG, was dependent on the true OR value.

eFigure 31 through eFigure 45 in the supplementary materials present the CP of the 95% CIs. The CP for EX, HG, and F2 is generally larger than the nominal level under the null hypothesis. For example, when *n* = 1,000, $\pi_{x_{1}}=0.05$ , and *n_e_* = 5 in scenario 4, the CPs for EX, HG, and F2 are 97.9%, 99.5%, and 98.2%, respectively. By contrast, the CP for FIR is lower than the nominal level when $\pi_{x_{1}}=0.05$ and *n_e_* = 5 because of the bias in the OR for FIR. Under the alternative hypothesis, the CPs for the EX, F1, and F2 are larger than the nominal level. For example, when *n* = 1,000, $\pi_{x_{1}}=0.05$ , *n_e_* = 5, and true OR = 4 in scenario 4, the CPs for EX, F1, and F2 are 99.2%, 99.1%, and 99.6%, respectively. By contrast, the CP for the HG is lower than the nominal level because the range of the credible intervals is narrow. For example, when *n* = 1,000, $\pi_{x_{1}}=0.05$ , and *n_e_* = 20, the CP for HG is 80.7%.

In summary, our simulation indicates that the bias of the OR from the Bayesian methods is smaller than that estimated using the ML, FIR, and EX in the simulation settings we have set.

## APPLICATION

The example data came from a cohort study on obstetric care and neonatal death.^33^ The binary outcome variable is death, for which 14 covariates are collected. A total of 17 deaths are observed among 2,992 births. A logistic regression model with all 14 variables converges but produces extremely inflated estimates because of the sparseness of the data.^5^ For example, the adjusted OR for hydramnios, which is one of the covariates, is 60, and only 1 of the 10 participants with hydramnios died. The unadjusted OR for hydramnios is 21.0, calculated (1/9)/(16/2,966).

The outcome is analyzed using a multiple logistic regression model including eight categorical covariates (hydramnios [2 levels], non-white [2 levels], placental or cord anomaly [2 levels], twins or triplets [2 levels], and malpresentation [2 levels], gestational age [3 levels], isoimmunization [3 levels], and labor progress [4 levels]) with a high association with death (OR ≤1/1.9 or ≥1.9 from Table 1 in Sullivan & Greenland (2013)^5^) to stabilize the model. All the covariates are included in the model as quantitative variables based on Sullivan & Greenland (2013).^5^

The estimated ORs for hydramnios from the single and multiple logistic models are shown in Table 2. In the multivariate logistic analysis, the ML and Firth’s estimates of the adjusted OR would be suitably inflated. Although the estimated ORs from the exact method and the Bayesian approach with *N*(0, 100) prior tended to 1, the estimated ORs may remain upward biased. These results are supported by our simulation under scenario 6. In conclusion, we recommend the Bayesian methods using log *F*-type priors or the hyper-*ɡ* prior for this case study, although we do not know the true magnitude of the OR for the real data. The supplementary materials contain the data set and SAS code used to implement these analyses.

**Table 2.** Unadjusted and adjusted odds ratios and their 95% confidence or credible intervals for hydramnios

Method	Unadjusted OR (95% CI)	Adjusted OR (95% CI)
ML method	20.6 (1.1–119.6)	50.9 (2.4–376.7)
Firth’s method	28.4 (2.9–134.8)	61.8 (5.6–368.0)
Exact method	20.5 (0.4–164.2)	44.9 (0.8–454.7)
Mid *P*-type exact method	20.5 (0.9–136.1)	44.9 (1.7–363.0)
Bayesian method with *N*(0, 100) prior	14.7 (0.5–105.4)	34.6 (0.8–315.7)
Bayesian method with hyper-*ɡ* prior	9.6 (0.5–69.8)	5.5 (0.4–54.6)
Bayesian data augmentation with log *F*(1, 1)	11.2 (0.5–83.1)	21.9 (0.7–209.6)
Bayesian data augmentation with log *F*(2, 2)	5.6 (0.4–53.6)	7.8 (0.4–107.9)

ML, maximum likelihood; OR, odds ratio.

## DISCUSSION

We reviewed several methods to handle small-sample and sparse data issues within logistic regression analyses and evaluated the bias of the OR from each method using simulation studies. We also listed the properties for each method in Table 3. When the number of events is relatively small, and the levels of the covariate of interest are considerably unbalanced, the ML, Firth’s, and exact methods are not recommended. For example, when there are fewer than 10 events and the proportion of study participants with an exposure of interest is less than 0.1, an OR estimated using the above three methods should be interpreted with caution, even in a univariate logistic regression analysis. A well-known conventional rule of thumb for sample size is to ensure at least 10 events per explanatory variable (EPV) in the context of logistic regression modeling.^34^ However, van Smeden et al (2019)^35^ and Riley et al (2019)^36^ have expressed concerns about the EPV ≥10 rule, and proposed alternative methods to determine the sample size for logistic prediction models. Further, the EPV rule is too strict and oversimplified; the sample size should be determined using not only EPV but also other quantities, such as the strength of effects or the correlation structure of exploratory variables.^37^^,^^38^ Our simulation indicates that the imbalance in the level of covariates as well as the number of events strongly affect the magnitude of the bias of the OR, emphasizing the significance of the imbalance of covariates when determining the sample size and selecting the estimation methods.

**Table 3.** Properties of methods for logistic regression analysis

Method	Bias for OR	Property and recommendation
ML method	Severe	Should not be used when *π_x_*_1_ ≤ 0.1 and *n_e_* ≤ 10.
Firth’s method	Severe	Should not be used when *π_x_*_1_ ≤ 0.1 and *n_e_* ≤ 10.
Exact method	Moderate	Confidence interval is too wide. Computation is intensive when large sample sizes or many covariates.
Mid *P*-type exact method	Moderate	Confidence interval is narrower than for exact method. Computation is intensive for large sample sizes or many covariates.
Bayesian method with *N*(0, 100) prior	Moderate	Relatively better than ML method.
Bayesian method with hyper-*ɡ* prior	Mild	Bias is the smallest among eight methods under null hypothesis; it is, however, unignorable under alternative hypothesis.
Bayesian data augmentation with log *F*(1, 1)	Mild	Correspond to 95% prior intervals for OR of 1/648 to 648. Setting prior mean of 0 underestimates OR under alternative hypothesis. Computation is easy.
Bayesian data augmentation with log *F*(2, 2)	Mild	Correspond to 95% prior intervals for OR of 1/39 to 39. The magnitude of shrinkage to 1 of OR is greater than log *F*(1, 1). Setting prior mean of 0 underestimates OR under alternative hypothesis. Computation is easy.

ML, maximum likelihood; *n_e_*, expected number of events; *π_x_*_1_, proportion of study participants with exposure *x*_1_ of interest; OR, odds ratio.

This study focused on sparse data with categorical covariates, but perfect separation can occur even when there are quantitative covariates. Our simulation results can be generalized for cases where there are quantitative covariates. In our simulation, scenarios 5 and 6 set categorical covariates with three levels and with four levels; these covariates are modeled as quantitative variables based on Sullivan & Greenland (2013).^5^ The results of scenarios 5 and 6 do not differ from those of other scenarios with only binominal covariates.

Greenland et al (2016) stated that the sparse data bias of OR could be more serious than the bias removed by covariate adjustment.^2^ However, to the best of our knowledge, there is no insightful study that has simultaneously evaluated the “sparse data bias” and “covariate omission bias.” To construct the best possible logistic model in practice, the covariate omission bias should be considered and studied in future research.

The Bayesian methods using the log *F*-type priors and hyper-*ɡ* prior are preferable in our simulation. All the estimated log ORs from the Bayesian methods are shrunk to zero and the estimates are close to the null hypothesis (log OR = 0) because we set the prior mean parameter ν=0 of β . Furthermore, the hyper-*ɡ* prior is preferable when the null hypothesis is true, and the log *F*-type priors are preferable when the alternative hypothesis is true. This is due to the fact that the magnitude of shrinkage with the hyper-*ɡ* prior is relatively larger than that with log *F*-type priors, because the prior variance of β for the hyper-*ɡ* is frequently smaller than that for log *F*(1, 1) and *F*(2, 2) priors in our settings. The probabilities that the prior variance for the hyper-*ɡ* is smaller than that for log *F* priors, are 95.2% for the log *F*(1, 1) prior and 86.8% for the log *F*(2, 2) prior.^39^ In addition, the posterior median of parameter *ɡ* is distributed with a median of 1.40 (interquartile range, 0.75–3.55) in our simulation, and the posterior variance of β for the hyper-*ɡ* tends to be smaller than that for log *F*(1, 1) and *F*(2, 2) priors.

The choice of priors would be controversial in the Bayesian analyses because of its arbitrariness in specifying its distribution. In general, epidemiological researchers would expect the alternative hypothesis to be true because they are interested in finding some effect (difference or association) in the study. In this context, some of them might be tempted to use the log *F*-type priors (especially, log *F*(1,1) prior) rather than the hyper-*ɡ* prior because they want to find statistical significance, even though the significance is not scientifically meaningful. In confirmatory clinical trials for drug development, establishing a treatment effect based on a primary analysis that is clearly conservative represents evidence of efficacy from a regulatory perspective.^40^ Conversely, Greenland & Mansournia (2015) mentioned that log *F*(1,1) is a better default prior in terms of simplicity and interpretation.^12^ It is important to evaluate the robustness of the analysis results and primary conclusions of the study because bias can occur in subtle or unknown ways, and its effect is not measurable in actual data directly.^41^ This robustness is evaluated through a sensitivity analysis. Thus, sensitivity analyses should be conducted using several priors in the study.

Although the Bayesian methods based on the hyper-*ɡ* prior seem objective, arbitrariness will arise when setting the prior mean ν of β . Setting the prior mean ν=0 can avoid arbitrary choices. Unless there is sufficient evidence to specify a larger value than zero for the prior mean, ν=0 is reasonable. However, this choice tends to excessively shrink the estimated log OR to zero under the alternative hypothesis. The estimated OR would be overcorrected and underestimated. In future studies, we intend to develop a method for objectively determining prior means of regression coefficients based on collected data to avoid arbitrariness and overcorrection. In addition, the OR from the hyper-*ɡ* prior may depend on the specification of the prior variance and parameter *a*. In our study, the Bayesian analysis based on the hyper-*ɡ* prior was set to the prior covariance matrix $\Sigma =\text{diag}(\frac{1}{2})$ and *a* = 4 based on Held & Sauter (2017),^27^ as mentioned in the methods section. However, it would be difficult to specify the prior variance and the parameter *a* without arbitrariness if there were no existing study, such as Held & Sauter (2017).^27^

Small-sample bias can also arise within conditional logistic regression analysis for matched pairs data. The conditional ML estimate of the OR can be biased when the sample size for the number of matched pairs is small. In a matched-pair case-control study, Jewell (1984) pointed out that the bias of the OR is serious even when the sample size is 50, and the bias increases as the true OR increases.^42^ Greenland et al (2000) provided expansive insight to small-sample bias in conditional logistic regression analysis for matched pairs data and introduced the exact and Bayesian methods for reducing the bias of the OR.^43^ Further, they applied the ML-bias corrected method provided by Schaefer (1983),^1^ the Haldane correction, which adds 1/2 to each cell in the contingency table (here, pair count), and the Laplace correction, which adds 1 to each cell. Firth’s method, described in the methods section above, has been modified to conditional logistic regression analysis for matched case-control data. Sun et al (2010) provided a modified conditional score function to eliminate the first-order bias of the conditional ML estimator based on Firth’s approach.^44^ The data augmentation methods introduced above can additionally be applied to conditional and unconditional logistic regression analysis.^45^

In conclusion, Firth’s method and the exact method do not reduce the sparse data bias sufficiently. The Bayesian methods using log *F*-type priors and the hyper-*ɡ* prior are the preferred choices when fitting logistic models to sparse data sets.

## SUPPLEMENTARY MATERIAL

eTable 1. Simulation condition

eTable 2. Simulation list

eFigure 1. Distribution of simulated OR under true OR = 1 in scenario 1 (top, *n* = 100; bottom, *n* = 300). The square represents the median and the error bar represents quartiles 1 and 3. The solid horizontal line is the true OR value. EX: exact method; F1: Bayesian data augmentation with log *F*(1, 1); F2: Bayesian data augmentation with log *F*(2, 2); FIR: Firth’s method; HG: Bayesian method with hyper-*ɡ* prior; MDP: mid *P*-type exact method; ML: ML method; NP: Bayesian method with *N*(0, 100) prior.

eFigure 2. Distribution of simulated OR under true OR = 1 in scenario 1 (top, *n* = 1000; bottom, *n* = 3000). The square represents the median and the error bar represents quartiles 1 and 3. The solid horizontal line is the true OR value. EX: exact method; F1: Bayesian data augmentation with log *F*(1, 1); F2: Bayesian data augmentation with log *F*(2, 2); FIR: Firth’s method; HG: Bayesian method with hyper-*ɡ* prior; MDP: mid *P*-type exact method; ML: ML method; NP: Bayesian method with *N*(0, 100) prior.

eFigure 3. Distribution of simulated OR under true OR = 4 in scenario 1 (top, *n* = 100; bottom, *n* = 300). The square represents the median and the error bar represents quartiles 1 and 3. The solid horizontal line is the true OR value. EX: exact method; F1: Bayesian data augmentation with log *F*(1, 1); F2: Bayesian data augmentation with log *F*(2, 2); FIR: Firth’s method; HG: Bayesian method with hyper-*ɡ* prior; MDP: mid *P*-type exact method; ML: ML method; NP: Bayesian method with *N*(0, 100) prior.

eFigure 4. Distribution of simulated OR under true OR = 4 in scenario 1 (top, *n* = 1000; bottom, *n* = 3000). The square represents the median and the error bar represents quartiles 1 and 3. The solid horizontal line is the true OR value. EX: exact method; F1: Bayesian data augmentation with log *F*(1, 1); F2: Bayesian data augmentation with log *F*(2, 2); FIR: Firth’s method; HG: Bayesian method with hyper-*ɡ* prior; MDP: mid *P*-type exact method; ML: ML method; NP: Bayesian method with *N*(0, 100) prior.

eFigure 5. Distribution of simulated OR under true OR = 16 in scenario 1 (top, *n* = 100; bottom, *n* = 300). The square represents the median and the error bar represents quartiles 1 and 3. The solid horizontal line is the true OR value. EX: exact method; F1: Bayesian data augmentation with log *F*(1, 1); F2: Bayesian data augmentation with log *F*(2, 2); FIR: Firth’s method; HG: Bayesian method with hyper-*ɡ* prior; MDP: mid *P*-type exact method; ML: ML method; NP: Bayesian method with *N*(0, 100) prior.

eFigure 6. Distribution of simulated OR under true OR = 16 in scenario 1 (top, *n* = 1000; bottom, *n* = 3000). The square represents the median and the error bar represents quartiles 1 and 3. The solid horizontal line is the true OR value. EX: exact method; F1: Bayesian data augmentation with log *F*(1, 1); F2: Bayesian data augmentation with log *F*(2, 2); FIR: Firth’s method; HG: Bayesian method with hyper-*ɡ* prior; MDP: mid *P*-type exact method; ML: ML method; NP: Bayesian method with *N*(0, 100) prior.

eFigure 7. Distribution of simulated OR under true OR = 1 in scenario 2 (top, *n* = 100; bottom, *n* = 300). The square represents the median and the error bar represents quartiles 1 and 3. The solid horizontal line is the true OR value. EX: exact method; F1: Bayesian data augmentation with log *F*(1, 1); F2: Bayesian data augmentation with log *F*(2, 2); FIR: Firth’s method; HG: Bayesian method with hyper-*ɡ* prior; MDP: mid *P*-type exact method; ML: ML method; NP: Bayesian method with *N*(0, 100) prior.

eFigure 8. Distribution of simulated OR under true OR = 1 in scenario 2 (top, *n* = 1000; bottom, *n* = 3000). The square represents the median and the error bar represents quartiles 1 and 3. The solid horizontal line is the true OR value. EX: exact method; F1: Bayesian data augmentation with log *F*(1, 1); F2: Bayesian data augmentation with log *F*(2, 2); FIR: Firth’s method; HG: Bayesian method with hyper-*ɡ* prior; MDP: mid *P*-type exact method; ML: ML method; NP: Bayesian method with *N*(0, 100) prior.

eFigure 9. Distribution of simulated OR under true OR = 4 in scenario 2 (top, *n* = 100; bottom, *n* = 300). The square represents the median and the error bar represents quartiles 1 and 3. The solid horizontal line is the true OR value. EX: exact method; F1: Bayesian data augmentation with log *F*(1, 1); F2: Bayesian data augmentation with log *F*(2, 2); FIR: Firth’s method; HG: Bayesian method with hyper-*ɡ* prior; MDP: mid *P*-type exact method; ML: ML method; NP: Bayesian method with *N*(0, 100) prior.

eFigure 10. Distribution of simulated OR under true OR = 4 in scenario 2 (top, *n* = 1000; bottom, *n* = 3000). The square represents the median and the error bar represents quartiles 1 and 3. The solid horizontal line is the true OR value. EX: exact method; F1: Bayesian data augmentation with log *F*(1, 1); F2: Bayesian data augmentation with log *F*(2, 2); FIR: Firth’s method; HG: Bayesian method with hyper-*ɡ* prior; MDP: mid *P*-type exact method; ML: ML method; NP: Bayesian method with *N*(0, 100) prior.

eFigure 11. Distribution of simulated OR under true OR = 16 in scenario 2 (top, *n* = 100; bottom, *n* = 300). The square represents the median and the error bar represents quartiles 1 and 3. The solid horizontal line is the true OR value. EX: exact method; F1: Bayesian data augmentation with log *F*(1, 1); F2: Bayesian data augmentation with log *F*(2, 2); FIR: Firth’s method; HG: Bayesian method with hyper-*ɡ* prior; MDP: mid *P*-type exact method; ML: ML method; NP: Bayesian method with *N*(0, 100) prior.

eFigure 12. Distribution of simulated OR under true OR = 16 in scenario 2 (top, *n* = 1000; bottom, *n* = 3000). The square represents the median and the error bar represents quartiles 1 and 3. The solid horizontal line is the true OR value. EX: exact method; F1: Bayesian data augmentation with log *F*(1, 1); F2: Bayesian data augmentation with log *F*(2, 2); FIR: Firth’s method; HG: Bayesian method with hyper-*ɡ* prior; MDP: mid *P*-type exact method; ML: ML method; NP: Bayesian method with *N*(0, 100) prior.

eFigure 13. Distribution of simulated OR under true OR = 1 in scenario 3 (top, *n* = 100; bottom, *n* = 300). The square represents the median and the error bar represents quartiles 1 and 3. The solid horizontal line is the true OR value. EX: exact method; F1: Bayesian data augmentation with log *F*(1, 1); F2: Bayesian data augmentation with log *F*(2, 2); FIR: Firth’s method; HG: Bayesian method with hyper-*ɡ* prior; MDP: mid *P*-type exact method; ML: ML method; NP: Bayesian method with *N*(0, 100) prior.

eFigure 14. Distribution of simulated OR under true OR = 1 in scenario 3 (top, *n* = 1000; bottom, *n* = 3000). The square represents the median and the error bar represents quartiles 1 and 3. The solid horizontal line is the true OR value. EX: exact method; F1: Bayesian data augmentation with log *F*(1, 1); F2: Bayesian data augmentation with log *F*(2, 2); FIR: Firth’s method; HG: Bayesian method with hyper-*ɡ* prior; MDP: mid *P*-type exact method; ML: ML method; NP: Bayesian method with *N*(0, 100) prior.

eFigure 15. Distribution of simulated OR under true OR = 4 in scenario 3 (top, *n* = 100; bottom, *n* = 300). The square represents the median and the error bar represents quartiles 1 and 3. The solid horizontal line is the true OR value. EX: exact method; F1: Bayesian data augmentation with log *F*(1, 1); F2: Bayesian data augmentation with log *F*(2, 2); FIR: Firth’s method; HG: Bayesian method with hyper-*ɡ* prior; MDP: mid *P*-type exact method; ML: ML method; NP: Bayesian method with *N*(0, 100) prior.

eFigure 16. Distribution of simulated OR under true OR = 4 in scenario 3 (top, *n* = 1000; bottom, *n* = 3000). The square represents the median and the error bar represents quartiles 1 and 3. The solid horizontal line is the true OR value. EX: exact method; F1: Bayesian data augmentation with log *F*(1, 1); F2: Bayesian data augmentation with log *F*(2, 2); FIR: Firth’s method; HG: Bayesian method with hyper-*ɡ* prior; MDP: mid *P*-type exact method; ML: ML method; NP: Bayesian method with *N*(0, 100) prior.

eFigure 17. Distribution of simulated OR under true OR = 16 in scenario 3 (top, *n* = 100; bottom, *n* = 300). The square represents the median and the error bar represents quartiles 1 and 3. The solid horizontal line is the true OR value. EX: exact method; F1: Bayesian data augmentation with log *F*(1, 1); F2: Bayesian data augmentation with log *F*(2, 2); FIR: Firth’s method; HG: Bayesian method with hyper-*ɡ* prior; MDP: mid *P*-type exact method; ML: ML method; NP: Bayesian method with *N*(0, 100) prior.

eFigure 18. Distribution of simulated OR under true OR = 16 in scenario 3 (top, *n* = 1000; bottom, *n* = 3000). The square represents the median and the error bar represents quartiles 1 and 3. The solid horizontal line is the true OR value. EX: exact method; F1: Bayesian data augmentation with log *F*(1, 1); F2: Bayesian data augmentation with log *F*(2, 2); FIR: Firth’s method; HG: Bayesian method with hyper-*ɡ* prior; MDP: mid *P*-type exact method; ML: ML method; NP: Bayesian method with *N*(0, 100) prior.

eFigure 19. Distribution of simulated OR under true OR = 1 in scenario 4 (top, *n* = 100; bottom, *n* = 300). The square represents the median and the error bar represents quartiles 1 and 3. The solid horizontal line is the true OR value. EX: exact method; F1: Bayesian data augmentation with log *F*(1, 1); F2: Bayesian data augmentation with log *F*(2, 2); FIR: Firth’s method; HG: Bayesian method with hyper-*ɡ* prior; MDP: mid *P*-type exact method; ML: ML method; NP: Bayesian method with *N*(0, 100) prior.

eFigure 20. Distribution of simulated OR under true OR = 1 in scenario 4 (top, *n* = 1000; bottom, *n* = 3000). The square represents the median and the error bar represents quartiles 1 and 3. The solid horizontal line is the true OR value. EX: exact method; F1: Bayesian data augmentation with log *F*(1, 1); F2: Bayesian data augmentation with log *F*(2, 2); FIR: Firth’s method; HG: Bayesian method with hyper-*ɡ* prior; MDP: mid *P*-type exact method; ML: ML method; NP: Bayesian method with *N*(0, 100) prior.

eFigure 21. Distribution of simulated OR under true OR = 4 in scenario 4 (top, *n* = 100; bottom, *n* = 300). The square represents the median and the error bar represents quartiles 1 and 3. The solid horizontal line is the true OR value. EX: exact method; F1: Bayesian data augmentation with log *F*(1, 1); F2: Bayesian data augmentation with log *F*(2, 2); FIR: Firth’s method; HG: Bayesian method with hyper-*ɡ* prior; MDP: mid *P*-type exact method; ML: ML method; NP: Bayesian method with *N*(0, 100) prior.

eFigure 22. Distribution of simulated OR under true OR = 4 in scenario 4 (top, *n* = 1000; bottom, *n* = 3000). The square represents the median and the error bar represents quartiles 1 and 3. The solid horizontal line is the true OR value. EX: exact method; F1: Bayesian data augmentation with log *F*(1, 1); F2: Bayesian data augmentation with log *F*(2, 2); FIR: Firth’s method; HG: Bayesian method with hyper-*ɡ* prior; MDP: mid *P*-type exact method; ML: ML method; NP: Bayesian method with *N*(0, 100) prior.

eFigure 23. Distribution of simulated OR under true OR = 16 in scenario 4 (top, *n* = 100; bottom, *n* = 300). The square represents the median and the error bar represents quartiles 1 and 3. The solid horizontal line is the true OR value. EX: exact method; F1: Bayesian data augmentation with log *F*(1, 1); F2: Bayesian data augmentation with log *F*(2, 2); FIR: Firth’s method; HG: Bayesian method with hyper-*ɡ* prior; MDP: mid *P*-type exact method; ML: ML method; NP: Bayesian method with *N*(0, 100) prior.

eFigure 24. Distribution of simulated OR under true OR = 16 in scenario 4 (top, *n* = 1000; bottom, *n* = 3000). The square represents the median and the error bar represents quartiles 1 and 3. The solid horizontal line is the true OR value. EX: exact method; F1: Bayesian data augmentation with log *F*(1, 1); F2: Bayesian data augmentation with log *F*(2, 2); FIR: Firth’s method; HG: Bayesian method with hyper-*ɡ* prior; MDP: mid *P*-type exact method; ML: ML method; NP: Bayesian method with *N*(0, 100) prior.

eFigure 25. Distribution of simulated OR under true OR = 1 in scenario 5 (top, *n* = 100; bottom, *n* = 300). The square represents the median and the error bar represents quartiles 1 and 3. The solid horizontal line is the true OR value. EX: exact method; F1: Bayesian data augmentation with log *F*(1, 1); F2: Bayesian data augmentation with log *F*(2, 2); FIR: Firth’s method; HG: Bayesian method with hyper-*ɡ* prior; MDP: mid *P*-type exact method; ML: ML method; NP: Bayesian method with *N*(0, 100) prior.

eFigure 26. Distribution of simulated OR under true OR = 1 in scenario 5 (top, *n* = 1000; bottom, *n* = 3000). The square represents the median and the error bar represents quartiles 1 and 3. The solid horizontal line is the true OR value. EX: exact method; F1: Bayesian data augmentation with log *F*(1, 1); F2: Bayesian data augmentation with log *F*(2, 2); FIR: Firth’s method; HG: Bayesian method with hyper-*ɡ* prior; MDP: mid *P*-type exact method; ML: ML method; NP: Bayesian method with *N*(0, 100) prior.

eFigure 27. Distribution of simulated OR under true OR = 4 in scenario 5 (top, *n* = 100; bottom, *n* = 300). The square represents the median and the error bar represents quartiles 1 and 3. The solid horizontal line is the true OR value. EX: exact method; F1: Bayesian data augmentation with log *F*(1, 1); F2: Bayesian data augmentation with log *F*(2, 2); FIR: Firth’s method; HG: Bayesian method with hyper-*ɡ* prior; MDP: mid *P*-type exact method; ML: ML method; NP: Bayesian method with *N*(0, 100) prior.

eFigure 28. Distribution of simulated OR under true OR = 4 in scenario 5 (top, *n* = 1000; bottom, *n* = 3000). The square represents the median and the error bar represents quartiles 1 and 3. The solid horizontal line is the true OR value. EX: exact method; F1: Bayesian data augmentation with log *F*(1, 1); F2: Bayesian data augmentation with log *F*(2, 2); FIR: Firth’s method; HG: Bayesian method with hyper-*ɡ* prior; MDP: mid *P*-type exact method; ML: ML method; NP: Bayesian method with *N*(0, 100) prior.

eFigure 29. Distribution of simulated OR under true OR = 16 in scenario 5 (top, *n* = 100; bottom, *n* = 300). The square represents the median and the error bar represents quartiles 1 and 3. The solid horizontal line is the true OR value. EX: exact method; F1: Bayesian data augmentation with log *F*(1, 1); F2: Bayesian data augmentation with log *F*(2, 2); FIR: Firth’s method; HG: Bayesian method with hyper-*ɡ* prior; MDP: mid *P*-type exact method; ML: ML method; NP: Bayesian method with *N*(0, 100) prior.

eFigure 30. Distribution of simulated OR under true OR = 16 in scenario 5 (top, *n* = 1000; bottom, *n* = 3000). The square represents the median and the error bar represents quartiles 1 and 3. The solid horizontal line is the true OR value. EX: exact method; F1: Bayesian data augmentation with log *F*(1, 1); F2: Bayesian data augmentation with log *F*(2, 2); FIR: Firth’s method; HG: Bayesian method with hyper-*ɡ* prior; MDP: mid *P*-type exact method; ML: ML method; NP: Bayesian method with *N*(0, 100) prior.

eFigure 31. 95% CI coverage probability under true OR = 1 in scenario 1 (top, *n* = 100; bottom, *n* = 1000). EX: exact method; F1: Bayesian data augmentation with log *F*(1, 1); F2: Bayesian data augmentation with log *F*(2, 2); FIR: Firth’s method; HG: Bayesian method with hyper-*ɡ* prior; MDP: mid *P*-type exact method; ML: ML method; NP: Bayesian method with *N*(0, 100) prior.

eFigure 32. 95% CI coverage probability under true OR = 4 in scenario 1 (top, *n* = 100; bottom, *n* = 1000). EX: exact method; F1: Bayesian data augmentation with log *F*(1, 1); F2: Bayesian data augmentation with log *F*(2, 2); FIR: Firth’s method; HG: Bayesian method with hyper-*ɡ* prior; MDP: mid *P*-type exact method; ML: ML method; NP: Bayesian method with *N*(0, 100) prior.

eFigure 33. 95% CI coverage probability under true OR = 16 in scenario 1 (top, *n* = 100; bottom, *n* = 1000). EX: exact method; F1: Bayesian data augmentation with log *F*(1, 1); F2: Bayesian data augmentation with log *F*(2, 2); FIR: Firth’s method; HG: Bayesian method with hyper-*ɡ* prior; MDP: mid *P*-type exact method; ML: ML method; NP: Bayesian method with *N*(0, 100) prior.

eFigure 34. 95% CI coverage probability under true OR = 1 in scenario 2 (top, *n* = 100; bottom, *n* = 1000). EX: exact method; F1: Bayesian data augmentation with log *F*(1, 1); F2: Bayesian data augmentation with log *F*(2, 2); FIR: Firth’s method; HG: Bayesian method with hyper-*ɡ* prior; MDP: mid *P*-type exact method; ML: ML method; NP: Bayesian method with *N*(0, 100) prior.

eFigure 35. 95% CI coverage probability under true OR = 4 in scenario 2 (top, *n* = 100; bottom, *n* = 1000). EX: exact method; F1: Bayesian data augmentation with log *F*(1, 1); F2: Bayesian data augmentation with log *F*(2, 2); FIR: Firth’s method; HG: Bayesian method with hyper-*ɡ* prior; MDP: mid *P*-type exact method; ML: ML method; NP: Bayesian method with *N*(0, 100) prior.

eFigure 36. 95% CI coverage probability under true OR = 16 in scenario 2 (top, *n* = 100; bottom, *n* = 1000). EX: exact method; F1: Bayesian data augmentation with log *F*(1, 1); F2: Bayesian data augmentation with log *F*(2, 2); FIR: Firth’s method; HG: Bayesian method with hyper-*ɡ* prior; MDP: mid *P*-type exact method; ML: ML method; NP: Bayesian method with *N*(0, 100) prior.

eFigure 37. 95% CI coverage probability under true OR = 1 in scenario 3 (top, *n* = 100; bottom, *n* = 1000). EX: exact method; F1: Bayesian data augmentation with log *F*(1, 1); F2: Bayesian data augmentation with log *F*(2, 2); FIR: Firth’s method; HG: Bayesian method with hyper-*ɡ* prior; MDP: mid *P*-type exact method; ML: ML method; NP: Bayesian method with *N*(0, 100) prior.

eFigure 38. 95% CI coverage probability under true OR = 4 in scenario 3 (top, *n* = 100; bottom, *n* = 1000). EX: exact method; F1: Bayesian data augmentation with log *F*(1, 1); F2: Bayesian data augmentation with log *F*(2, 2); FIR: Firth’s method; HG: Bayesian method with hyper-*ɡ* prior; MDP: mid *P*-type exact method; ML: ML method; NP: Bayesian method with *N*(0, 100) prior.

eFigure 39. 95% CI coverage probability under true OR = 16 in scenario 3 (top, *n* = 100; bottom, *n* = 1000). EX: exact method; F1: Bayesian data augmentation with log *F*(1, 1); F2: Bayesian data augmentation with log *F*(2, 2); FIR: Firth’s method; HG: Bayesian method with hyper-*ɡ* prior; MDP: mid *P*-type exact method; ML: ML method; NP: Bayesian method with *N*(0, 100) prior.

eFigure 40. 95% CI coverage probability under true OR = 1 in scenario 4 (top, *n* = 100; bottom, *n* = 1000). EX: exact method; F1: Bayesian data augmentation with log *F*(1, 1); F2: Bayesian data augmentation with log *F*(2, 2); FIR: Firth’s method; HG: Bayesian method with hyper-*ɡ* prior; MDP: mid *P*-type exact method; ML: ML method; NP: Bayesian method with *N*(0, 100) prior.

eFigure 41. 95% CI coverage probability under true OR = 4 in scenario 4 (top, *n* = 100; bottom, *n* = 1000). EX: exact method; F1: Bayesian data augmentation with log *F*(1, 1); F2: Bayesian data augmentation with log *F*(2, 2); FIR: Firth’s method; HG: Bayesian method with hyper-*ɡ* prior; MDP: mid *P*-type exact method; ML: ML method; NP: Bayesian method with *N*(0, 100) prior.

eFigure 42. 95% CI coverage probability under true OR = 16 in scenario 4 (top, *n* = 100; bottom, *n* = 1000). EX: exact method; F1: Bayesian data augmentation with log *F*(1, 1); F2: Bayesian data augmentation with log *F*(2, 2); FIR: Firth’s method; HG: Bayesian method with hyper-*ɡ* prior; MDP: mid *P*-type exact method; ML: ML method; NP: Bayesian method with *N*(0, 100) prior.

eFigure 43. 95% CI coverage probability under true OR = 1 in scenario 5 (top, *n* = 100; bottom, *n* = 1000). EX: exact method; F1: Bayesian data augmentation with log *F*(1, 1); F2: Bayesian data augmentation with log *F*(2, 2); FIR: Firth’s method; HG: Bayesian method with hyper-*ɡ* prior; MDP: mid *P*-type exact method; ML: ML method; NP: Bayesian method with *N*(0, 100) prior.

eFigure 44. 95% CI coverage probability under true OR = 4 in scenario 5 (top, *n* = 100; bottom, *n* = 1000). EX: exact method; F1: Bayesian data augmentation with log *F*(1, 1); F2: Bayesian data augmentation with log *F*(2, 2); FIR: Firth’s method; HG: Bayesian method with hyper-*ɡ* prior; MDP: mid *P*-type exact method; ML: ML method; NP: Bayesian method with *N*(0, 100) prior.

eFigure 45. 95% CI coverage probability under true OR = 16 in scenario 5 (top, *n* = 100; bottom, *n* = 1000). EX: exact method; F1: Bayesian data augmentation with log *F*(1, 1); F2: Bayesian data augmentation with log *F*(2, 2); FIR: Firth’s method; HG: Bayesian method with hyper-*ɡ* prior; MDP: mid *P*-type exact method; ML: ML method; NP: Bayesian method with *N*(0, 100) prior.

eFigure 46. Distribution of simulated OR in scenario 6. The square represents the median and the error bar represents quartiles 1 and 3. The solid horizontal line is the true OR value. EX: exact method; F1: Bayesian data augmentation with log *F*(1, 1); F2: Bayesian data augmentation with log *F*(2, 2); FIR: Firth’s method; HG: Bayesian method with hyper-*ɡ* prior; MDP: mid *P*-type exact method; ML: ML method; NP: Bayesian method with *N*(0, 100) prior.

eFigure 47. 95% CI coverage probability (CP) in scenario 6. When true OR = 1, the CPs for ML, FIR, MDP, and NP are 43.3, 13.3, 59.5, and 67.4%, respectively. The CP for FIR is 61.0% when true OR = 4. EX: exact method; F1: Bayesian data augmentation with log *F*(1, 1); F2: Bayesian data augmentation with log *F*(2, 2); FIR: Firth’s method; HG: Bayesian method with hyper-*ɡ* prior; MDP: mid *P*-type exact method; ML: ML method; NP: Bayesian method with *N*(0, 100) prior.

eFigure 48: Distribution of simulated OR under true OR = 1 in scenario 1 (top, *n* = 100; bottom, *n* = 1000). The square represents the median and the error bar represents quartiles 1 and 3. The solid horizontal line is the true OR value. EX: exact method; F1: Bayesian data augmentation with log *F*(1, 1); F2: Bayesian data augmentation with log *F*(2, 2); FIR: Firth’s method; HG: Bayesian method with hyper-*ɡ* prior; MDP: mid *P*-type exact method; NP: Bayesian method with *N*(0, 100) prior.

eFigure 49: Distribution of simulated OR under true OR = 4 in scenario 1 (top, *n* = 100; bottom, *n* = 1000). The square represents the median and the error bar represents quartiles 1 and 3. The solid horizontal line is the true OR value. EX: exact method; F1: Bayesian data augmentation with log *F*(1, 1); F2: Bayesian data augmentation with log *F*(2, 2); FIR: Firth’s method; HG: Bayesian method with hyper-*ɡ* prior; MDP: mid *P*-type exact method; NP: Bayesian method with *N*(0, 100) prior.

eFigure 50: Distribution of simulated OR under true OR = 16 in scenario 1 (top, *n* = 100; bottom, *n* = 1000). The square represents the median and the error bar represents quartiles 1 and 3. The solid horizontal line is the true OR value. EX: exact method; F1: Bayesian data augmentation with log *F*(1, 1); F2: Bayesian data augmentation with log *F*(2, 2); FIR: Firth’s method; HG: Bayesian method with hyper-*ɡ* prior; MDP: mid *P*-type exact method; NP: Bayesian method with *N*(0, 100) prior.

eFigure 51. Distribution of simulated OR under true OR = 1 in scenario 2 (top, *n* = 100; bottom, *n* = 1000). The square represents the median and the error bar represents quartiles 1 and 3. The solid horizontal line is the true OR value. EX: exact method; F1: Bayesian data augmentation with log *F*(1, 1); F2: Bayesian data augmentation with log *F*(2, 2); FIR: Firth’s method; HG: Bayesian method with hyper-*ɡ* prior; MDP: mid *P*-type exact method; NP: Bayesian method with *N*(0, 100) prior.

eFigure 52. Distribution of simulated OR under true OR = 4 in scenario 2 (top, *n* = 100; bottom, *n* = 1000). The square represents the median and the error bar represents quartiles 1 and 3. The solid horizontal line is the true OR value. EX: exact method; F1: Bayesian data augmentation with log *F*(1, 1); F2: Bayesian data augmentation with log *F*(2, 2); FIR: Firth’s method; HG: Bayesian method with hyper-*ɡ* prior; MDP: mid *P*-type exact method; NP: Bayesian method with *N*(0, 100) prior.

eFigure 53. Distribution of simulated OR under true OR = 16 in scenario 2 (top, *n* = 100; bottom, *n* = 1000). The square represents the median and the error bar represents quartiles 1 and 3. The solid horizontal line is the true OR value. EX: exact method; F1: Bayesian data augmentation with log *F*(1, 1); F2: Bayesian data augmentation with log *F*(2, 2); FIR: Firth’s method; HG: Bayesian method with hyper-*ɡ* prior; MDP: mid *P*-type exact method; NP: Bayesian method with *N*(0, 100) prior.

eFigure 54. Distribution of simulated OR under true OR = 1 in scenario 3 (top, *n* = 100; bottom, *n* = 1000). The square represents the median and the error bar represents quartiles 1 and 3. The solid horizontal line is the true OR value. EX: exact method; F1: Bayesian data augmentation with log *F*(1, 1); F2: Bayesian data augmentation with log *F*(2, 2); FIR: Firth’s method; HG: Bayesian method with hyper-*ɡ* prior; MDP: mid *P*-type exact method; NP: Bayesian method with *N*(0, 100) prior.

eFigure 55. Distribution of simulated OR under true OR = 4 in scenario 3 (top, *n* = 100; bottom, *n* = 1000). The square represents the median and the error bar represents quartiles 1 and 3. The solid horizontal line is the true OR value. EX: exact method; F1: Bayesian data augmentation with log *F*(1, 1); F2: Bayesian data augmentation with log *F*(2, 2); FIR: Firth’s method; HG: Bayesian method with hyper-*ɡ* prior; MDP: mid *P*-type exact method; NP: Bayesian method with *N*(0, 100) prior.

eFigure 56. Distribution of simulated OR under true OR = 16 in scenario 3 (top, *n* = 100; bottom, *n* = 1000). The square represents the median and the error bar represents quartiles 1 and 3. The solid horizontal line is the true OR value. EX: exact method; F1: Bayesian data augmentation with log *F*(1, 1); F2: Bayesian data augmentation with log *F*(2, 2); FIR: Firth’s method; HG: Bayesian method with hyper-*ɡ* prior; MDP: mid *P*-type exact method; NP: Bayesian method with *N*(0, 100) prior.

eFigure 57. Distribution of simulated OR under true OR = 1 in scenario 4 (top, *n* = 100; bottom, *n* = 1000). The square represents the median and the error bar represents quartiles 1 and 3. The solid horizontal line is the true OR value. EX: exact method; F1: Bayesian data augmentation with log *F*(1, 1); F2: Bayesian data augmentation with log *F*(2, 2); FIR: Firth’s method; HG: Bayesian method with hyper-*ɡ* prior; MDP: mid *P*-type exact method; NP: Bayesian method with *N*(0, 100) prior.

eFigure 58. Distribution of simulated OR under true OR = 4 in scenario 4 (top, *n* = 100; bottom, *n* = 1000). The square represents the median and the error bar represents quartiles 1 and 3. The solid horizontal line is the true OR value. EX: exact method; F1: Bayesian data augmentation with log *F*(1, 1); F2: Bayesian data augmentation with log *F*(2, 2); FIR: Firth’s method; HG: Bayesian method with hyper-*ɡ* prior; MDP: mid *P*-type exact method; NP: Bayesian method with *N*(0, 100) prior.

eFigure 59. Distribution of simulated OR under true OR = 16 in scenario 4 (top, *n* = 100; bottom, *n* = 1000). The square represents the median and the error bar represents quartiles 1 and 3. The solid horizontal line is the true OR value. EX: exact method; F1: Bayesian data augmentation with log *F*(1, 1); F2: Bayesian data augmentation with log *F*(2, 2); FIR: Firth’s method; HG: Bayesian method with hyper-*ɡ* prior; MDP: mid *P*-type exact method; NP: Bayesian method with *N*(0, 100) prior.

eFigure 60. Distribution of simulated OR under true OR = 1 in scenario 5 (top, *n* = 100; bottom, *n* = 1000). The square represents the median and the error bar represents quartiles 1 and 3. The solid horizontal line is the true OR value. EX: exact method; F1: Bayesian data augmentation with log *F*(1, 1); F2: Bayesian data augmentation with log *F*(2, 2); FIR: Firth’s method; HG: Bayesian method with hyper-*ɡ* prior; MDP: mid *P*-type exact method; NP: Bayesian method with *N*(0, 100) prior.

eFigure 61. Distribution of simulated OR under true OR = 4 in scenario 5 (top, *n* = 100; bottom, *n* = 1000). The square represents the median and the error bar represents quartiles 1 and 3. The solid horizontal line is the true OR value. EX: exact method; F1: Bayesian data augmentation with log *F*(1, 1); F2: Bayesian data augmentation with log *F*(2, 2); FIR: Firth’s method; HG: Bayesian method with hyper-*ɡ* prior; MDP: mid *P*-type exact method; NP: Bayesian method with *N*(0, 100) prior.

eFigure 62. Distribution of simulated OR under true OR = 16 in scenario 5 (top, *n* = 100; bottom, *n* = 1000). The square represents the median and the error bar represents quartiles 1 and 3. The solid horizontal line is the true OR value. EX: exact method; F1: Bayesian data augmentation with log *F*(1, 1); F2: Bayesian data augmentation with log *F*(2, 2); FIR: Firth’s method; HG: Bayesian method with hyper-*ɡ* prior; MDP: mid *P*-type exact method; NP: Bayesian method with *N*(0, 100) prior.

eFigure 63. Distribution of simulated OR in scenario 6. The square represents the median and the error bar represents quartiles 1 and 3. The solid horizontal line is the true OR value. EX: exact method; F1: Bayesian data augmentation with log *F*(1, 1); F2: Bayesian data augmentation with log *F*(2, 2); FIR: Firth’s method; HG: Bayesian method with hyper-*ɡ* prior; MDP: mid *P*-type exact method; NP: Bayesian method with *N*(0, 100) prior.

eFigure 64. 95% CI coverage probability (CP) in scenario 6. The CP is 49.2% for HG and is 71.2% for F2 when true OR = 16. EX: exact method; F1: Bayesian data augmentation with log *F*(1, 1); F2: Bayesian data augmentation with log *F*(2, 2); FIR: Firth’s method; HG: Bayesian method with hyper-*ɡ* prior; MDP: mid *P*-type exact method; NP: Bayesian method with *N*(0, 100) prior.

## References

[1] Schaefer RL. Bias correction in maximum likelihood logistic regression. Stat Med. 1983;2:71–78. 10.1002/sim.4780020108 6648121

[2] Greenland S, Mansournia MA, Altman DG. Sparse data bias: a problem hiding in plain sight. BMJ. 2016;352:i1981. 10.1136/bmj.i1981 27121591

[3] Masuda K, Ishizu T, Niwa K, . Increased risk of thromboembolic events in adult congenital heart disease patients with atrial tachyarrhythmias. Int J Cardiol. 2017;234:69–75. 10.1016/j.ijcard.2017.02.004 28209388

[4] Ayubi E, Safiri S, Mansournia MA. Increased risk of thromboembolic events in adult congenital heart disease patients with atrial tachyarrhythmias: bias due to the data sparsity. Int J Cardiol. 2017;239:20. 10.1016/j.ijcard.2017.02.133 28560967

[5] Sullivan SG, Greenland S. Bayesian regression in SAS software. Int J Epidemiol. 2013;42:308–317. 10.1093/ije/dys213 23230299

[6] Firth D. Bias reduction of maximum likelihood estimates. Biometrika. 1993;80:27–38. 10.1093/biomet/80.1.27

[7] Hirji KF, Mehta CR, Patel NR. Exact inference for matched case-control studies. Biometrics. 1988;44:803–814. 10.2307/2531592 3203129

[8] Mehta CR, Patel NR. Exact logistic regression: theory and examples. Stat Med. 1995;14:2143–2160. 10.1002/sim.4780141908 8552893

[9] Greenland S. Prior data for non-normal priors. Stat Med. 2007;26:3578–3590. 10.1002/sim.2788 17216667

[10] Held L, Sabanés Bové D, Gravestock I. Approximate Bayesian model selection with the deviance statistic. Stat Sci. 2015;30:242–257. 10.1214/14-STS510

[11] Zellner A. On assessing prior distributions and Bayesian regression analysis with *ɡ*-prior distributions. *Bayesian Inference and Decision Techniques*. 1986;233–243.

[12] Greenland S, Mansournia MA. Penalization, bias reduction, and default priors in logistic and related categorical and survival regressions. Stat Med. 2015;34:3133–3143. 10.1002/sim.6537 26011599

[13] Mansournia MA, Geroldinger A, Greenland S, Heinze G. Separation in logistic regression: causes, consequences, and control. Am J Epidemiol. 2018;187:864–870. 10.1093/aje/kwx299 29020135

[14] Heinze G, Schemper M. A solution to the problem of separation in logistic regression. Stat Med. 2002;21:2409–2419. 10.1002/sim.1047 12210625

[15] Heinze G. A comparative investigation of methods for logistic regression with separated or nearly separated data. Stat Med. 2006;25:4216–4226. 10.1002/sim.2687 16955543

[16] Heinze G, Puhr R. Bias-reduced and separation-proof conditional logistic regression with small or sparse data sets. Stat Med. 2010;29:770–777. 10.1002/sim.3794 20213709

[17] Venzon DJ, Moolgavkar SH. A method for computing profile-likelihood-based confidence intervals. J Roy Stat Soc C. 1988;37:87–94. 10.2307/2347496

[18] Puhr R, Heinze G, Nold M, Lusa L, Geroldinger A. Firth’s logistic regression with rare events: accurate effect estimates and predictions? Stat Med. 2017;36:2302–2317. 10.1002/sim.7273 28295456

[19] Cordeiro GM, McCullagh P. Bias correction in generalized linear models. J R Stat Soc B. 1991;53:629–643. 10.1111/j.2517-6161.1991.tb01852.x

[20] Maiti T, Pradhan V. A comparative study of the bias corrected estimates in logistic regression. Stat Methods Med Res. 2008;17:621–634. 10.1177/0962280207084156 18375454

[21] Berry G, Armitage P. Mid-P confidence intervals: a brief review. J Roy Stat Soc D-STA. 1995;44:417–423.

[22] Lesaffre E, Lawson AB. *Bayesian biostatistics*. John Wiley & Sons; 2012.

[23] Bedrick EJ, Ronald C, Wesley J. A new perspective on priors for generalized linear models. J Am Stat Assoc. 1996;91:1450–1460. 10.1080/01621459.1996.10476713

[24] Chen MH, Ibrahim JG, Kim S. Properties and implementation of Jeffreys’s prior in binomial regression models. J Am Stat Assoc. 2008;103:1659–1664. 10.1198/016214508000000779 19436775 PMC2680313

[25] Gelman A, Jakulin A, Pittau MG, Su YS. A weakly informative default prior distribution for logistic and other regression models. Ann Appl Stat. 2008;2:1360–1383. 10.1214/08-AOAS191

[26] Hanson TE, Adam JB, Wesley OJ. Informative g-priors for logistic regression. Bayesian Anal. 2014;9:597–612. 10.1214/14-BA868

[27] Held L, Sauter R. Adaptive prior weighting in generalized regression. Biometrics. 2017;73:242–251. 10.1111/biom.12541 27192504

[28] Rahman MS, Sultana M. Performance of Firth- and log F-type penalized methods in risk prediction for small or sparse binary data. BMC Med Res Methodol. 2017;17:33. 10.1186/s12874-017-0313-9 28231767 PMC5324225

[29] Sabanés Bové D, Held L. Hyper-g priors for generalized linear models. Bayesian Anal. 2011;6:387–410. 10.1214/ba/1339616469

[30] Liang F, Paulo R, Molina G, Clyde MA, Berger JO. Mixtures of g priors for Bayesian variable selection. J Am Stat Assoc. 2008;103:410–423. 10.1198/016214507000001337

[31] Strawderman WE. Proper Bayes minimax estimators of the multivariate normal mean. Ann Math Stat. 1971;42:385–388. 10.1214/aoms/1177693528

[32] Cui W, George EI. Empirical Bayes vs. fully Bayes variable selection. J Stat Plan Inference. 2008;138:888–900. 10.1016/j.jspi.2007.02.011

[33] Neutra RR, Fienberg SE, Greenland S, Friedman EA. Effect of fetal monitoring on neonatal death rates. N Engl J Med. 1978;299:324–326. 10.1056/NEJM197808172990702 683265

[34] Peduzzi P, Concato J, Kemper E, Holford TR, Feinstein AR. A simulation study of the number of events per variable in logistic regression analysis. J Clin Epidemiol. 1996;49:1373–1379. 10.1016/S0895-4356(96)00236-3 8970487

[35] van Smeden M, Moons KG, de Groot JA, . Sample size for binary logistic prediction models: beyond events per variable criteria. Stat Methods Med Res. 2019;28:2455–2474. 10.1177/0962280218784726 29966490 PMC6710621

[36] Riley RD, Snell KI, Ensor J, . Minimum sample size for developing a multivariable prediction model: PART II-binary and time-to-event outcomes. Stat Med. 2019;38:1276–1296. 10.1002/sim.7992 30357870 PMC6519266

[37] Vittinghoff E, McCulloch CE. Relaxing the rule of ten events per variable in logistic and Cox regression. Am J Epidemiol. 2007;165:710–718. 10.1093/aje/kwk052 17182981

[38] Courvoisier DS, Combescure C, Agoritsas T, Gayet-Ageron A, Perneger TV. Performance of logistic regression modeling: Beyond the number of events per variable, the role of data structure. J Clin Epidemiol. 2011;64:993–1000. 10.1016/j.jclinepi.2010.11.012 21411281

[39] Brown BW, Spears FM, Levy LB. The log F: a distribution for all seasons. Comput Stat. 2002;17:47–58. 10.1007/s001800200098

[40] Committee for Medical Products for Human Use. *Guideline on missing data in confirmatory clinical trials*. London: European Medicines Evaluation Agency; 2010.

[41] ICH Guideline E9. Statistical principles for clinical trials. International Conference on Harmonisation, 1998.

[42] Jewell NP. Small-sample bias of point estimators of the odds ratio from matched sets. Biometrics. 1984;40:421–435. 10.2307/2531395 6487726

[43] Greenland S, Schwartzbaum JA, Finkle WD. Problems due to small samples and sparse data in conditional logistic regression analysis. Am J Epidemiol. 2000;151:531–539. 10.1093/oxfordjournals.aje.a010240 10707923

[44] Sun JX, Sinha S, Wang S, Maiti T. Bias reduction in conditional logistic regression. Stat Med. 2011;30:348–355. 10.1002/sim.4105 21225897

[45] Greenland S, Christensen R. Data augmentation priors for Bayesian and semi-Bayes analyses of conditional logistic and proportional hazards regression. Stat Med. 2001;20:2421–2428. 10.1002/sim.902 11512132

