# Publisher Correction: “Bias in odds ratios from logistic regression methods with sparse data sets”

**DOI:** 10.2188/jea.JE20220044

**Published:** 2023-06-05

**Authors:** Masahiko Gosho, Tomohiro Ohigashi, Kengo Nagashima, Yuri Ito, Kazushi Maruo

**Affiliations:** 1 Department of Biostatistics, Faculty of Medicine, University of Tsukuba, Ibaraki, Japan; 2 Graduate School of Comprehensive Human Sciences, University of Tsukuba, Ibaraki, Japan; 3 Department of Biostatistics, Tsukuba Clinical Research & Development Organization, University of Tsukuba, Ibaraki, Japan; 4 Research Center for Medical and Health Data Science, The Institute of Statistical Mathematics, Tokyo, Japan; 5 Department of Medical Statistics, Research & Development Center, Osaka Medical College, Osaka, Japan

In the accepted version of the above article [J Epidemiol, Accepted Article, doi:10.2188/jea.JE20210089],^1^ which was available online from September 25, 2021 to March 4, 2022, the article type was incorrectly written as “Original Article”. The correct article type is “Review Article”.

The correction has been made to the published article. We, the Journal of Epidemiology, cordially apologizes for this error.

Kota Katanoda
Editor in Chief
Journal of Epidemiology
