# Supplementary material for: Bias in Odds Ratios From Logistic Regression Methods With Sparse Data Sets
Source: J Epidemiol. 2023 Jun 5;33(6):265–75. doi: 10.2188/jea.JE20210089 (PMC10165217; doi:10.2188/jea.JE20210089)
Supplement: Supplementary file 1 [file je-33-265-s001.zip › JE20210089-eMaterial-accepted-33-4-clean.pdf]

Web Supplementary Materials for

"Bias in odds ratios from logistic regression methods with sparse data sets"

Simulation results

eFigure 1 through **eFigure 47** present the simulation results in scenarios 1 to 6 for the restricted cases where the maximum likelihood (ML) estimator exists. In scenario 5, the results are shown only when $n_e = 5$ and $\pi_{x_1} = 0.05$, $n_e = 10$ and $\pi_{x_1} = 0.05$, and $n_e = 10$ and $\pi_{x_1} = 0.1$ because the estimate for the exact method was not obtained when n_e and π_{x_1} are large. **eFigure 48** through **eFigure 64** present the simulation results in scenarios 1 to 6 for all cases without the restriction except for the ML method. In scenario 5, the results are shown only when $n_e = 5$ and $\pi_{x_1} = 0.05$, $n_e = 10$ and $\pi_{x_1} = 0.05$, and $n_e = 10$ and $\pi_{x_1} = 0.1$ because the estimate for the exact method was not obtained when n_e and π_{x_1} are large. The simulation condition and list are presented in **eTable 1** and **eTable 2**, respectively.

eTable 1. Simulation condition

Parameter	Level
Scenario	1 = the number of covariates is 1<br>2 = the number of covariates is 2 (independent)<br>3 = the number of covariates is 2 (correlated)<br>4 = the number of covariates is 4<br>5 = the number of covariates is 8<br>6 = the number of covariates is 8 based on the actual data
True OR for x_1	1, 4, and 16
Total sample size, n	100, 300, 1000, and 3000
Expected number of events, n_e	5, 10, and 20
Binominal probability for x_1, π_{x_1}	0.05, 0.1, and 0.2

eTable 2. Simulation list

eFigure number	Outcome	Scenario	True OR	Condition
Restricted cases where the maximum likelihood estimator exists				
1	OR	1	1	n = 100, n = 300
2	OR	1	1	n = 1000, n = 3000
3	OR	1	4	n = 100, n = 300
4	OR	1	4	n = 1000, n = 3000
5	OR	1	16	n = 100, n = 300
6	OR	1	16	n = 1000, n = 3000
7	OR	2	1	n = 100, n = 300

8	OR	2	1	n = 1000, n = 3000
9	OR	2	4	n = 100, n = 300
10	OR	2	4	n = 1000, n = 3000
11	OR	2	16	n = 100, n = 300
12	OR	2	16	n = 1000, n = 3000
13	OR	3	1	n = 100, n = 300
14	OR	3	1	n = 1000, n = 3000
15	OR	3	4	n = 100, n = 300
16	OR	3	4	n = 1000, n = 3000
17	OR	3	16	n = 100, n = 300
18	OR	3	16	n = 1000, n = 3000
19	OR	4	1	n = 100, n = 300
20	OR	4	1	n = 1000, n = 3000
21	OR	4	4	n = 100, n = 300
22	OR	4	4	n = 1000, n = 3000
23	OR	4	16	n = 100, n = 300
24	OR	4	16	n = 1000, n = 3000
25	OR	5	1	n = 100, n = 300
26	OR	5	1	n = 1000, n = 3000
27	OR	5	4	n = 100, n = 300
28	OR	5	4	n = 1000, n = 3000
29	OR	5	16	n = 100, n = 300
30	OR	5	16	n = 1000, n = 3000
31	CP of 95%CI	1	1	n = 100, n = 1000
32	CP of 95%CI	1	4	n = 100, n = 1000
33	CP of 95%CI	1	16	n = 100, n = 1000
34	CP of 95%CI	2	1	n = 100, n = 1000
35	CP of 95%CI	2	4	n = 100, n = 1000
36	CP of 95%CI	2	16	n = 100, n = 1000
37	CP of 95%CI	3	1	n = 100, n = 1000
38	CP of 95%CI	3	4	n = 100, n = 1000
39	CP of 95%CI	3	16	n = 100, n = 1000
40	CP of 95%CI	4	1	n = 100, n = 1000
41	CP of 95%CI	4	4	n = 100, n = 1000
42	CP of 95%CI	4	16	n = 100, n = 1000
43	CP of 95%CI	5	1	n = 100, n = 1000
44	CP of 95%CI	5	4	n = 100, n = 1000

45	CP of 95%CI	5	16	n = 100, n = 1000
46	OR	6	1, 4, 16	n = 2992
47	CP of 95%CI	6	1, 4, 16	n = 2992
All cases without the restriction except for the maximum likelihood method				
48	OR	1	1	n = 100, n = 1000
49	OR	1	4	n = 100, n = 1000
50	OR	1	16	n = 100, n = 1000
51	OR	2	1	n = 100, n = 1000
52	OR	2	4	n = 100, n = 1000
53	OR	2	16	n = 100, n = 1000
54	OR	3	1	n = 100, n = 1000
55	OR	3	4	n = 100, n = 1000
56	OR	3	16	n = 100, n = 1000
57	OR	4	1	n = 100, n = 1000
58	OR	4	4	n = 100, n = 1000
59	OR	4	16	n = 100, n = 1000
60	OR	5	1	n = 100, n = 1000
61	OR	5	4	n = 100, n = 1000
62	OR	5	16	n = 100, n = 1000
63	OR	6	1, 4, 16	n = 2992
64	CP of 95%CI	6	1, 4, 16	n = 2992

CI, confidence interval; CP, coverage probability; OR, odds ratio.

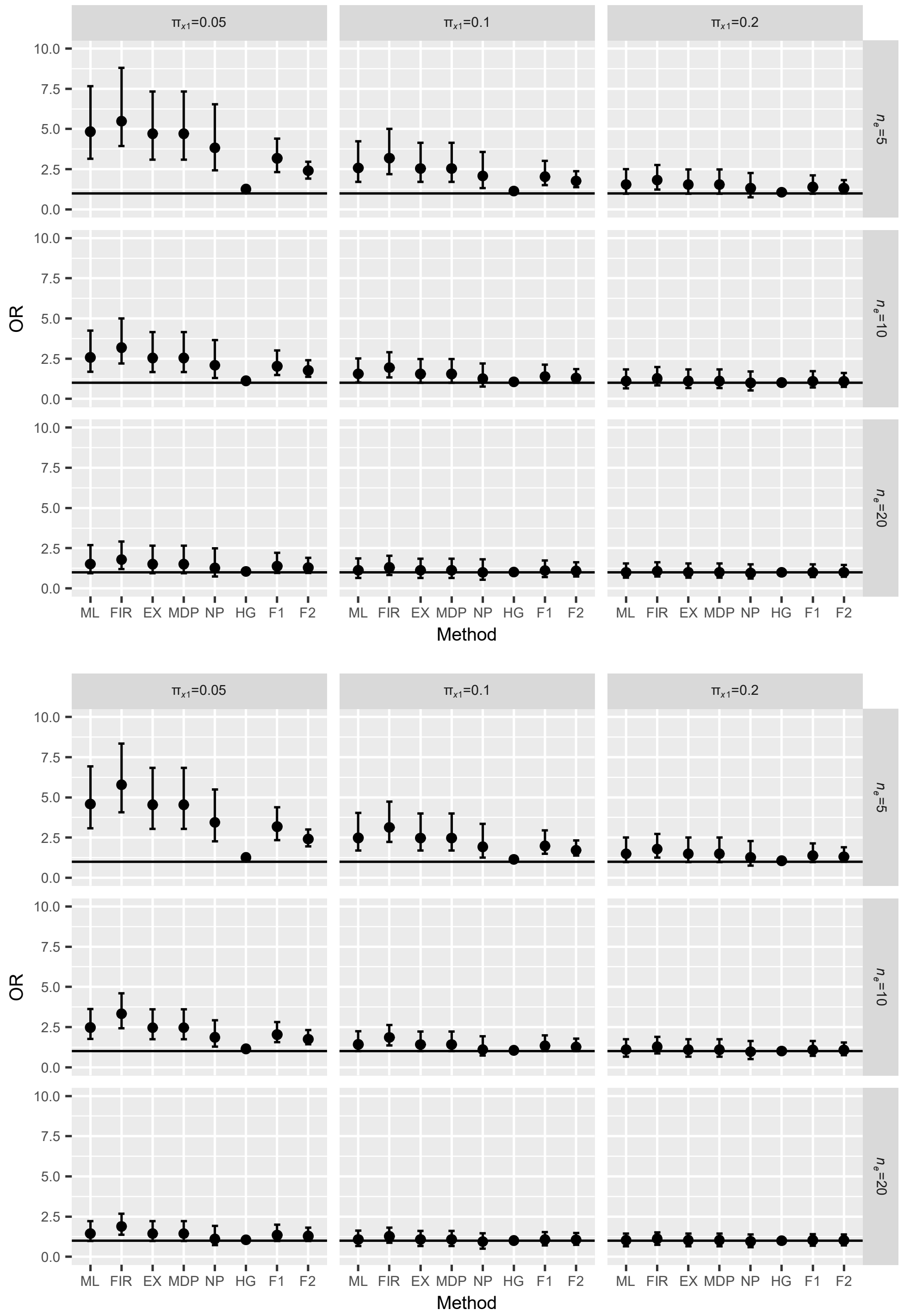

eFigure 1. Distribution of simulated OR under true OR = 1 in scenario 1 (top, n = 100; bottom, n = 300). The square represents the median and the error bar represents quartiles 1 and 3. The solid horizontal line is the true OR value. EX: exact method; F1: Bayesian data augmentation with log $F(1, 1)$; F2: Bayesian data augmentation with log $F(2, 2)$; FIR: Firth's method; HG: Bayesian method with hyper-g prior; MDP: mid P-type exact method; ML: ML method; NP: Bayesian method with $N(0, 100)$ prior.

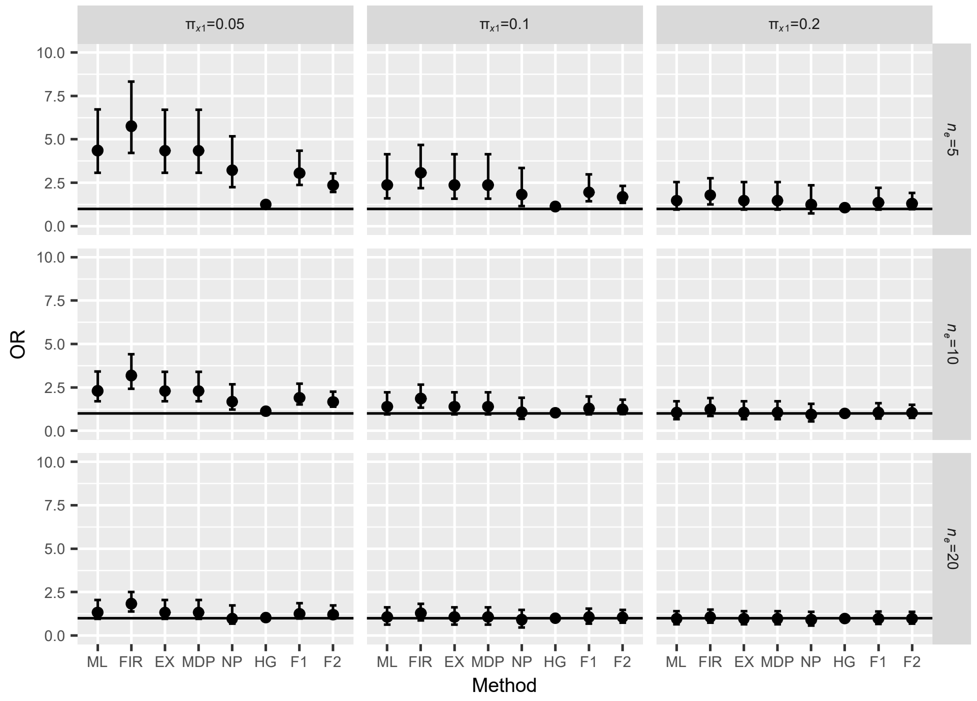

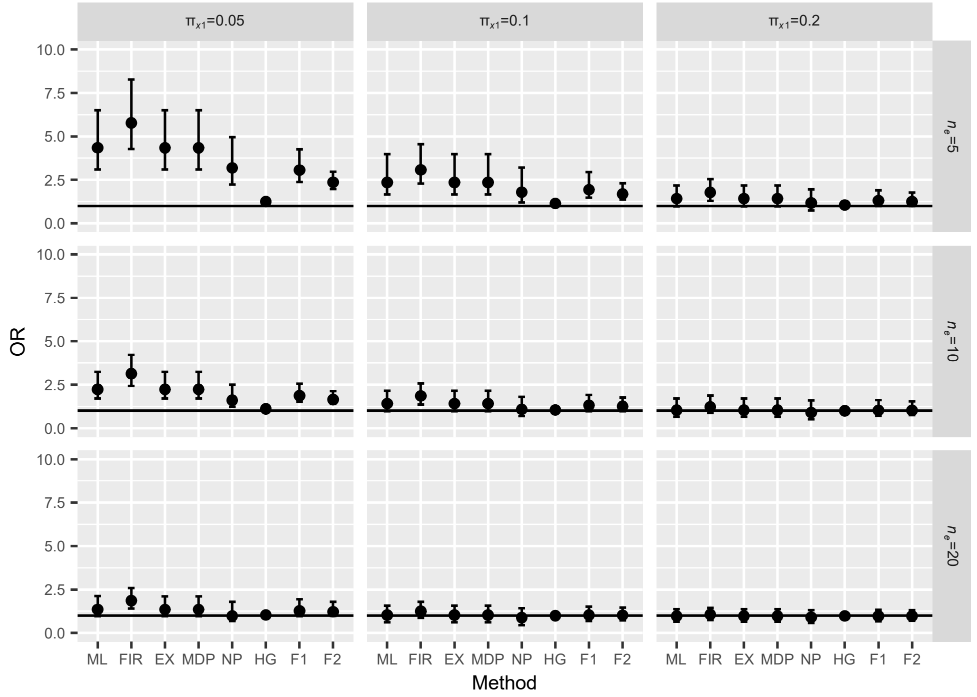

eFigure 2. Distribution of simulated OR under true OR = 1 in scenario 1 (top, n = 1000; bottom, n = 3000). The square represents the median and the error bar represents quartiles 1 and 3. The solid horizontal line is the true OR value. EX: exact method; F1: Bayesian data augmentation with log $F(1, 1)$; F2: Bayesian data augmentation with log $F(2, 2)$; FIR: Firth's method; HG: Bayesian method with hyper-g prior; MDP: mid P-type exact method; ML: ML method; NP: Bayesian method with $N(0, 100)$ prior.

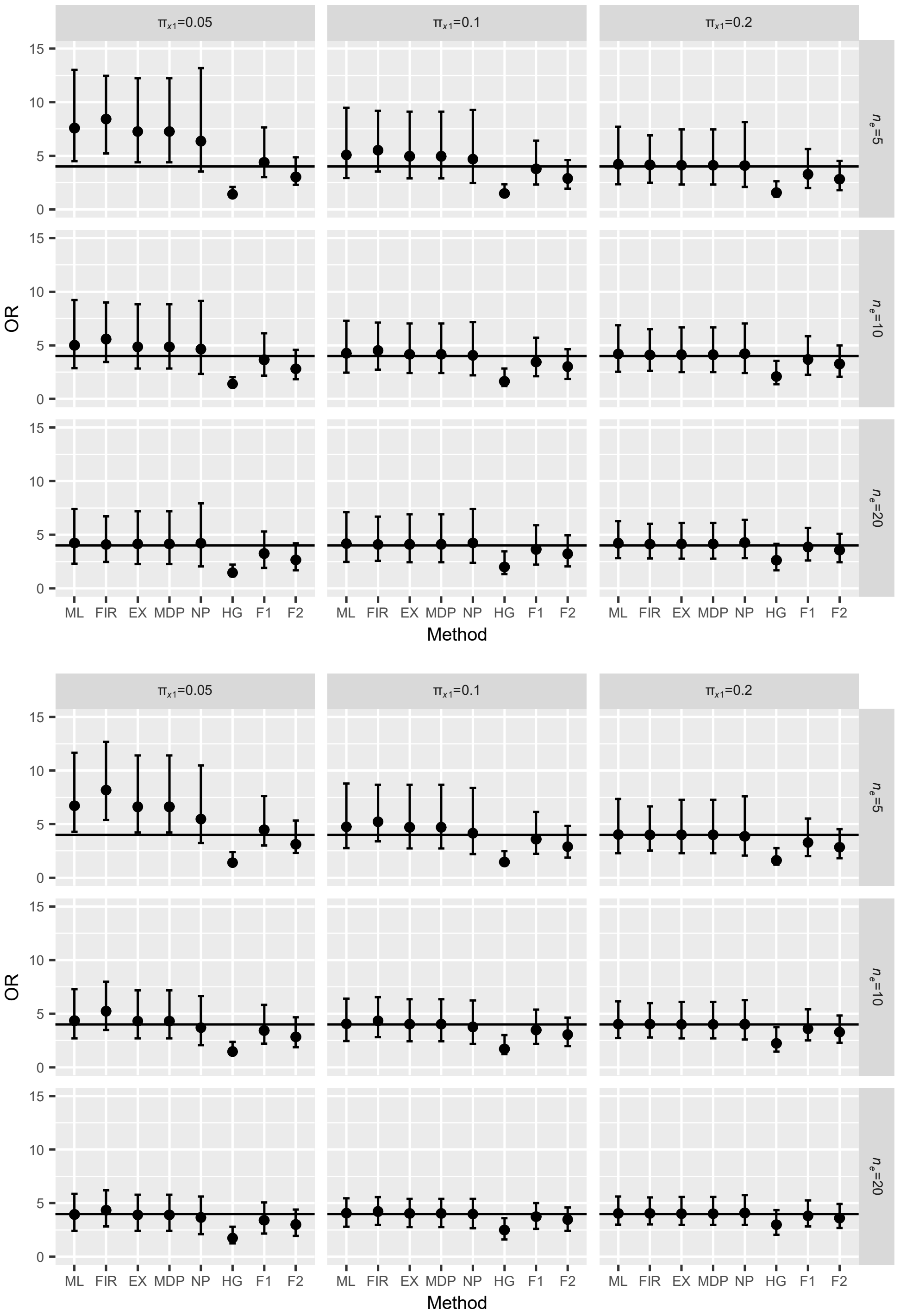

eFigure 3. Distribution of simulated OR under true OR = 4 in scenario 1 (top, n = 100; bottom, n = 300). The square represents the median and the error bar represents quartiles 1 and 3. The solid horizontal line is the true OR value. EX: exact method; F1: Bayesian data augmentation with log F(1, 1); F2: Bayesian data augmentation with log F(2, 2); FIR: Firth's method; HG: Bayesian method with hyper-g prior; MDP: mid P-type exact method; ML: ML method; NP: Bayesian method with N(0, 100) prior.

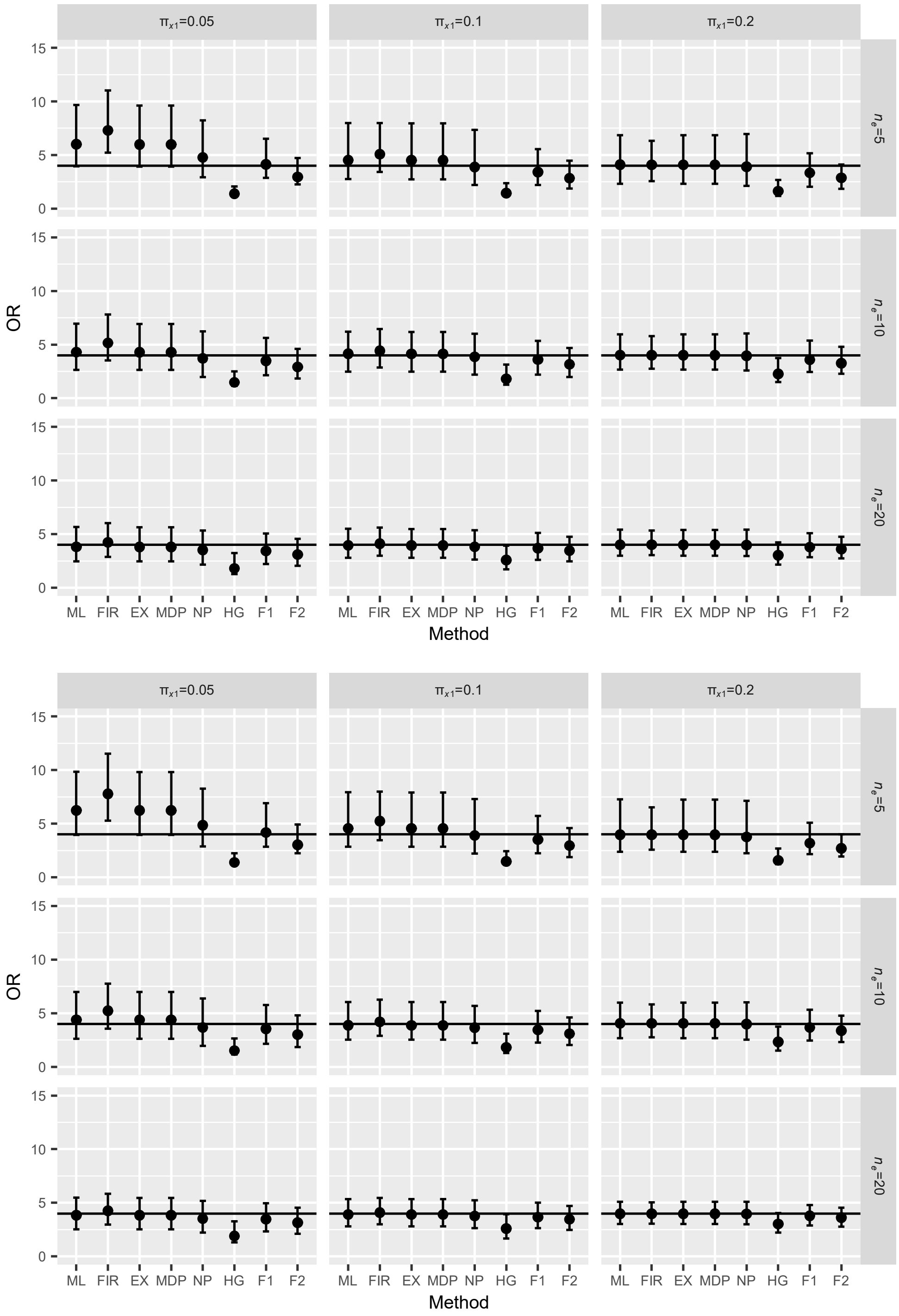

eFigure 4. Distribution of simulated OR under true OR = 4 in scenario 1 (top, n = 1000; bottom, n = 3000). The square represents the median and the error bar represents quartiles 1 and 3. The solid horizontal line is the true OR value. EX: exact method; F1: Bayesian data augmentation with log $F(1, 1)$; F2: Bayesian data augmentation with log $F(2, 2)$; FIR: Firth's method; HG: Bayesian method with hyper-g prior; MDP: mid P-type exact method; ML: ML method; NP: Bayesian method with $N(0, 100)$ prior.

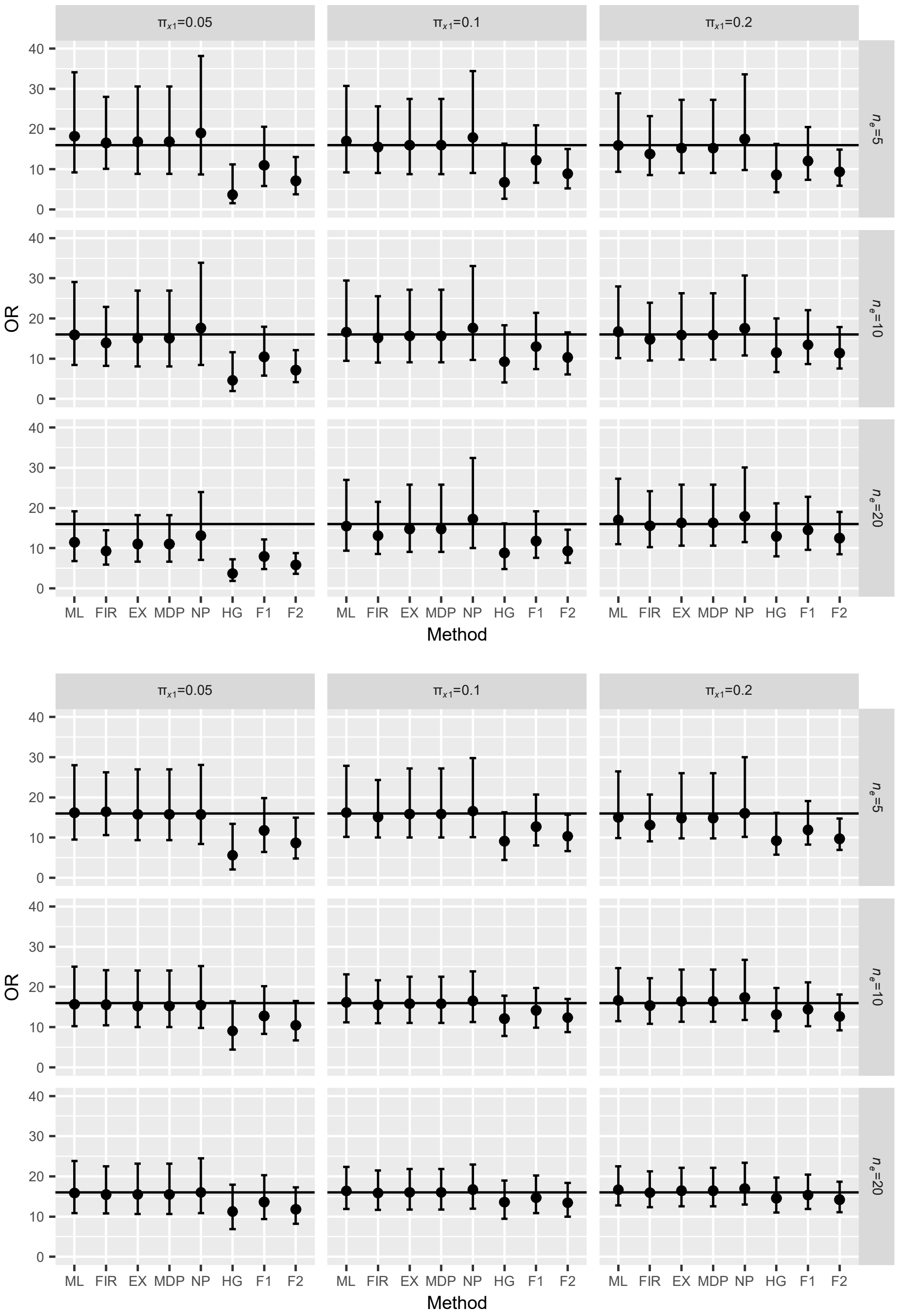

eFigure 5. Distribution of simulated OR under true OR = 16 in scenario 1 (top, n = 100; bottom, n = 300). The square represents the median and the error bar represents quartiles 1 and 3. The solid horizontal line is the true OR value. EX: exact method; F1: Bayesian data augmentation with log F(1, 1); F2: Bayesian data augmentation with log F(2, 2); FIR: Firth's method; HG: Bayesian method with hyper-g prior; MDP: mid P-type exact method; ML: ML method; NP: Bayesian method with N(0, 100) prior.

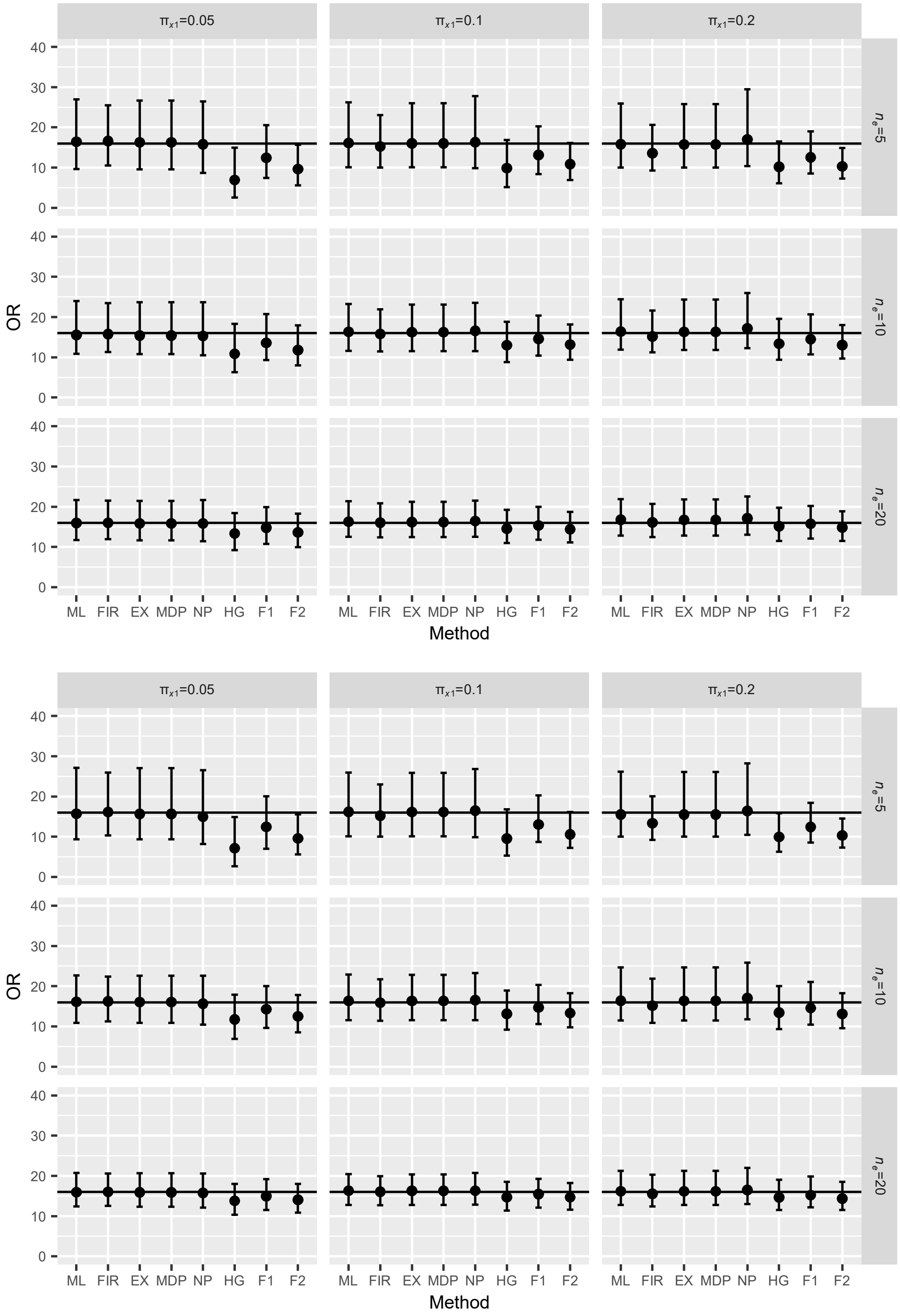

eFigure 6. Distribution of simulated OR under true OR = 16 in scenario 1 (top, n = 1000; bottom, n = 3000). The square represents the median and the error bar represents quartiles 1 and 3. The solid horizontal line is the true OR value. EX: exact method; F1: Bayesian data augmentation with log $F(1, 1)$; F2: Bayesian data augmentation with log $F(2, 2)$; FIR: Firth's method; HG: Bayesian method with hyper-g prior; MDP: mid P-type exact method; ML: ML method; NP: Bayesian method with $N(0, 100)$ prior.

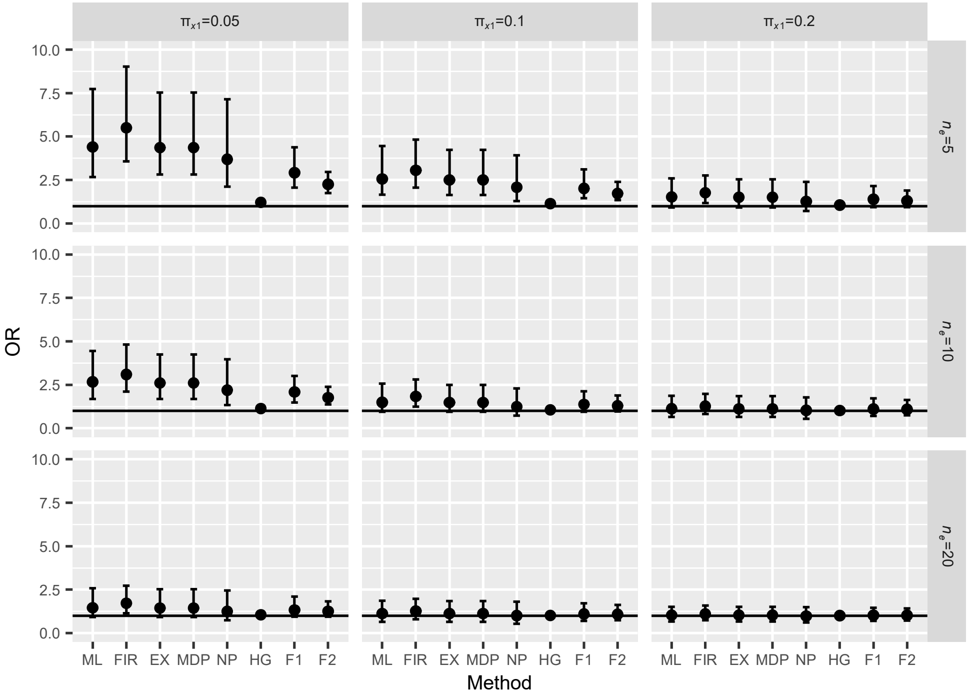

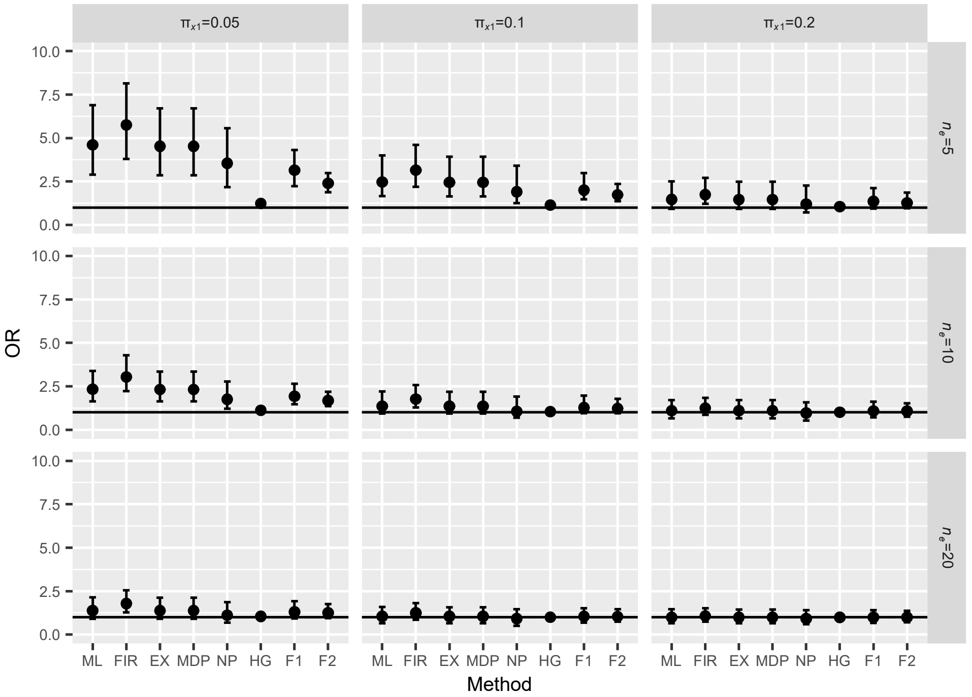

eFigure 7. Distribution of simulated OR under true OR = 1 in scenario 2 (top, n = 100; bottom, n = 300). The square represents the median and the error bar represents quartiles 1 and 3. The solid horizontal line is the true OR value. EX: exact method; F1: Bayesian data augmentation with log F(1, 1); F2: Bayesian data augmentation with log F(2, 2); FIR: Firth's method; HG: Bayesian method with hyper-g prior; MDP: mid P-type exact method; ML: ML method; NP: Bayesian method with N(0, 100) prior.

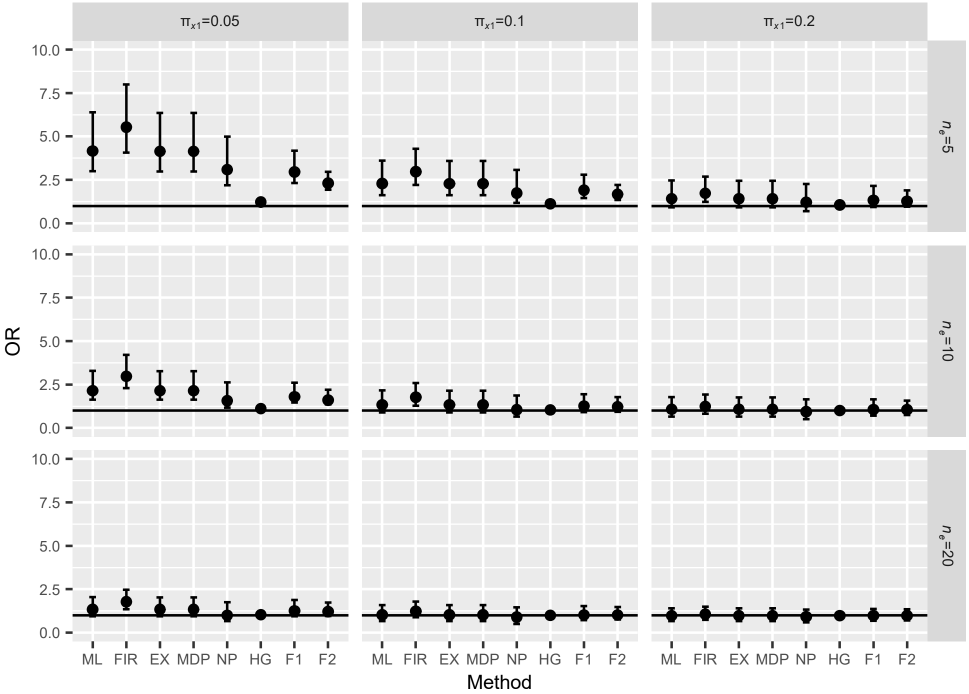

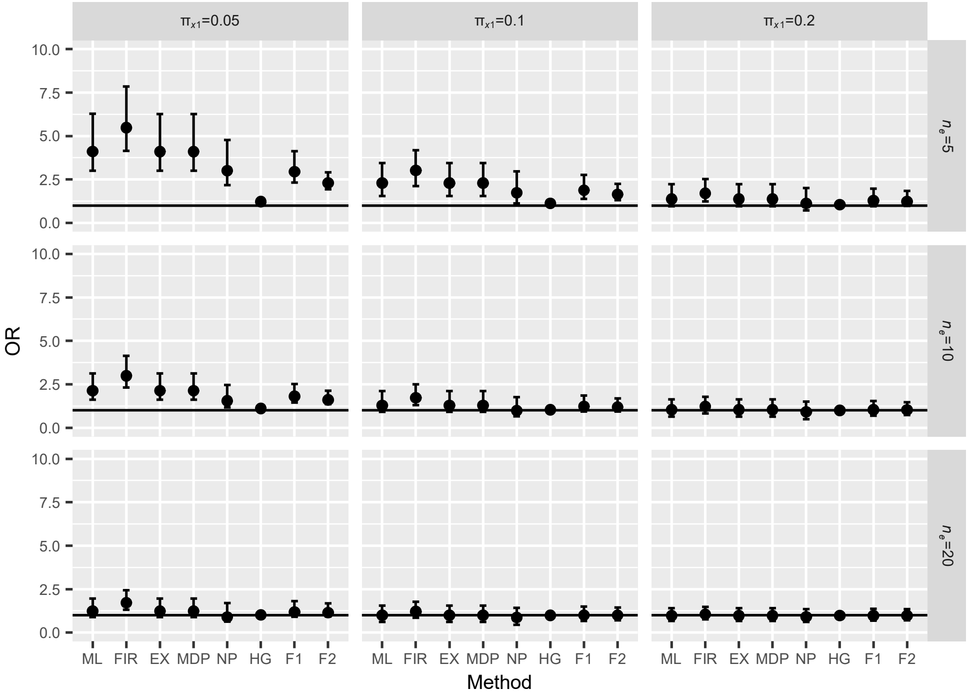

eFigure 8. Distribution of simulated OR under true OR = 1 in scenario 2 (top, n = 1000; bottom, n = 3000). The square represents the median and the error bar represents quartiles 1 and 3. The solid horizontal line is the true OR value. EX: exact method; F1: Bayesian data augmentation with log $F(1, 1)$; F2: Bayesian data augmentation with log $F(2, 2)$; FIR: Firth's method; HG: Bayesian method with hyper-g prior; MDP: mid P-type exact method; ML: ML method; NP: Bayesian method with $N(0, 100)$ prior.

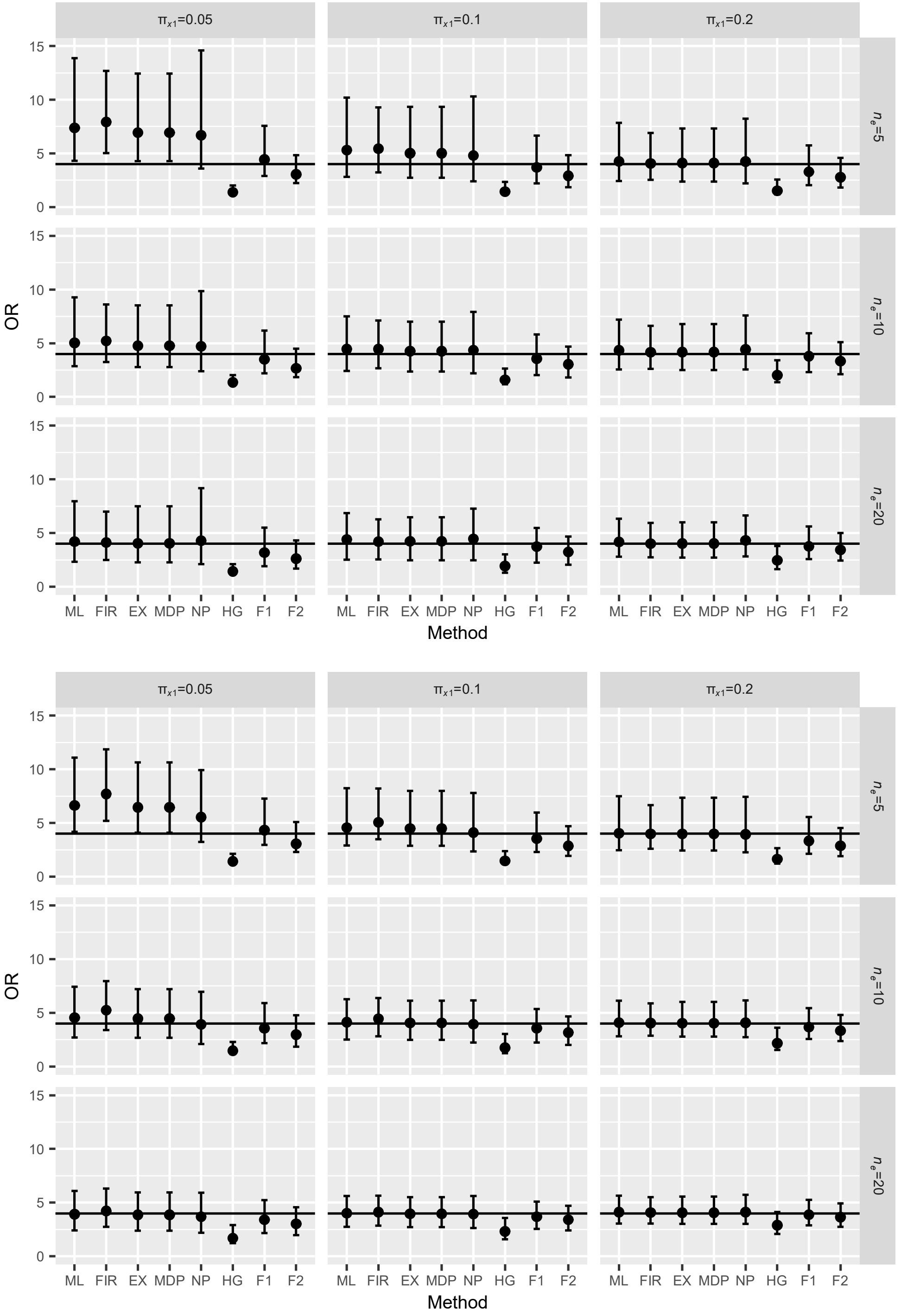

eFigure 9. Distribution of simulated OR under true OR = 4 in scenario 2 (top, n = 100; bottom, n = 300). The square represents the median and the error bar represents quartiles 1 and 3. The solid horizontal line is the true OR value. EX: exact method; F1: Bayesian data augmentation with log F(1, 1); F2: Bayesian data augmentation with log F(2, 2); FIR: Firth's method; HG: Bayesian method with hyper-g prior; MDP: mid P-type exact method; ML: ML method; NP: Bayesian method with N(0, 100) prior.

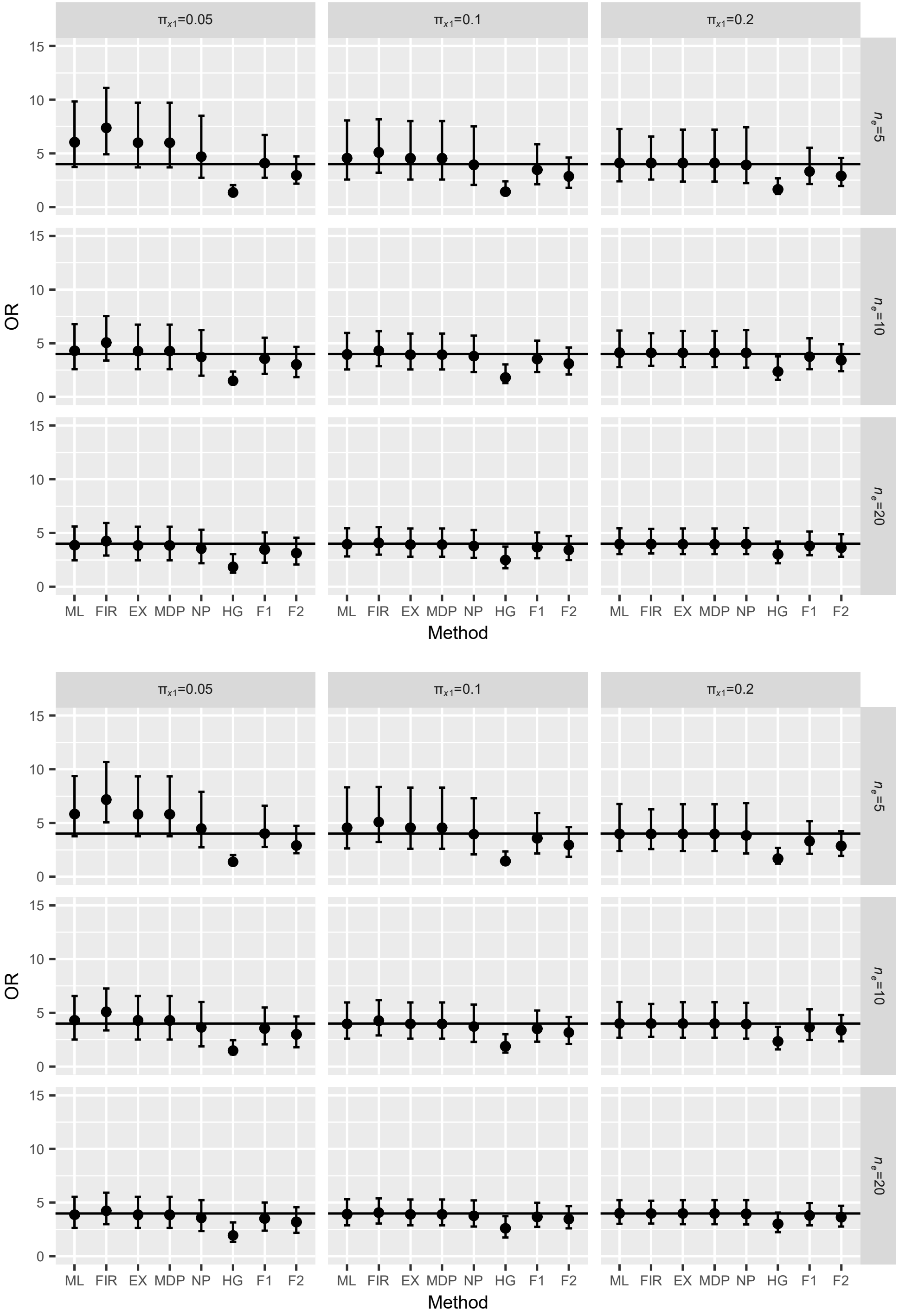

eFigure 10. Distribution of simulated OR under true OR = 4 in scenario 2 (top, n = 1000; bottom, n = 3000). The square represents the median and the error bar represents quartiles 1 and 3. The solid horizontal line is the true OR value. EX: exact method; F1: Bayesian data augmentation with log $F(1, 1)$; F2: Bayesian data augmentation with log $F(2, 2)$; FIR: Firth's method; HG: Bayesian method with hyper-g prior; MDP: mid P-type exact method; ML: ML method; NP: Bayesian method with $N(0, 100)$ prior.

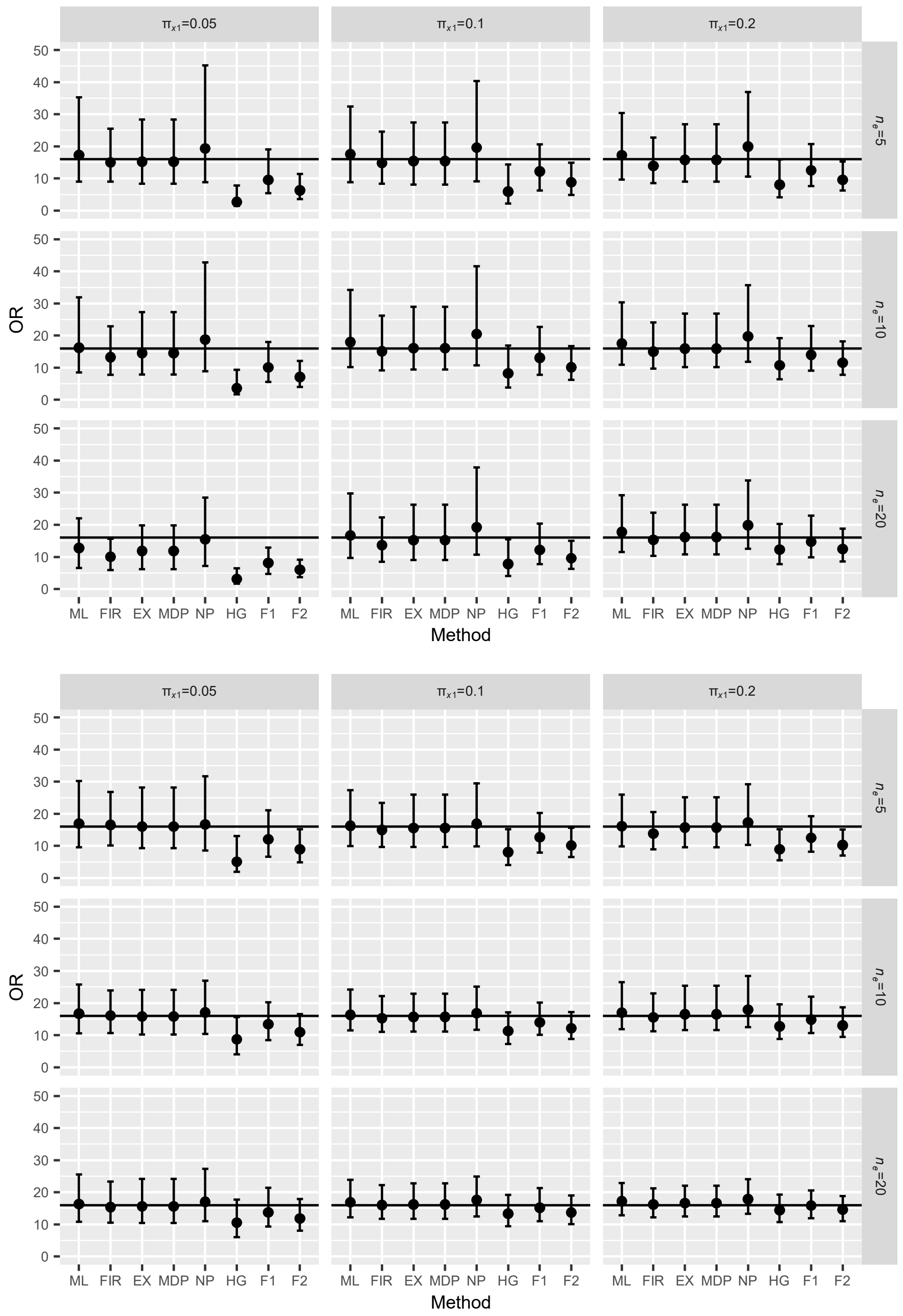

eFigure 11. Distribution of simulated OR under true OR = 16 in scenario 2 (top, n = 100; bottom, n = 300). The square represents the median and the error bar represents quartiles 1 and 3. The solid horizontal line is the true OR value. EX: exact method; F1: Bayesian data augmentation with log $F(1, 1)$; F2: Bayesian data augmentation with log $F(2, 2)$; FIR: Firth's method; HG: Bayesian method with hyper-g prior; MDP: mid P-type exact method; ML: ML method; NP: Bayesian method with $N(0, 100)$ prior.

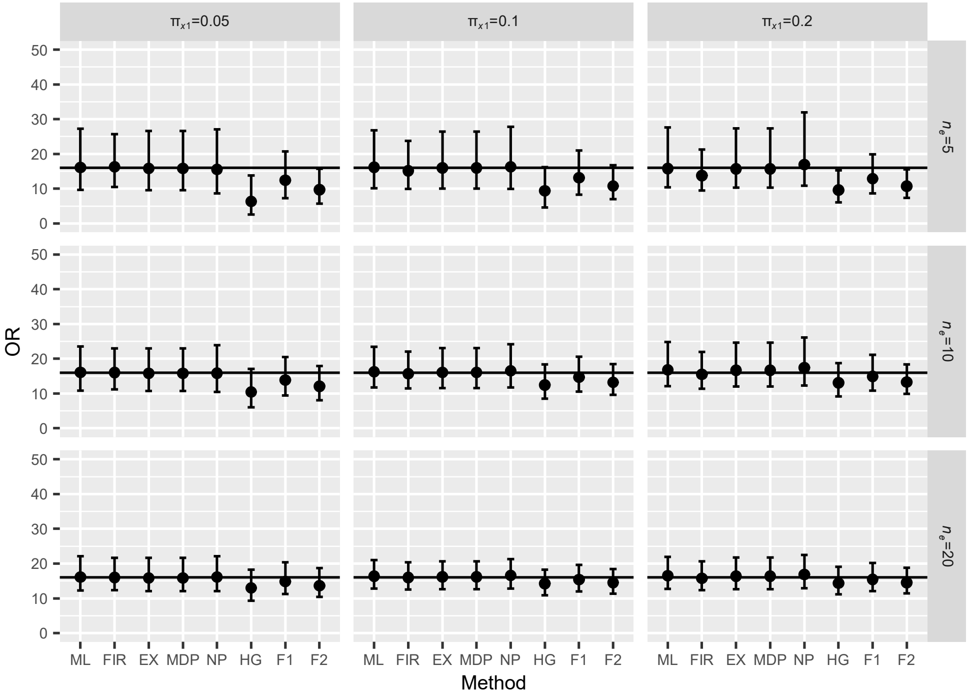

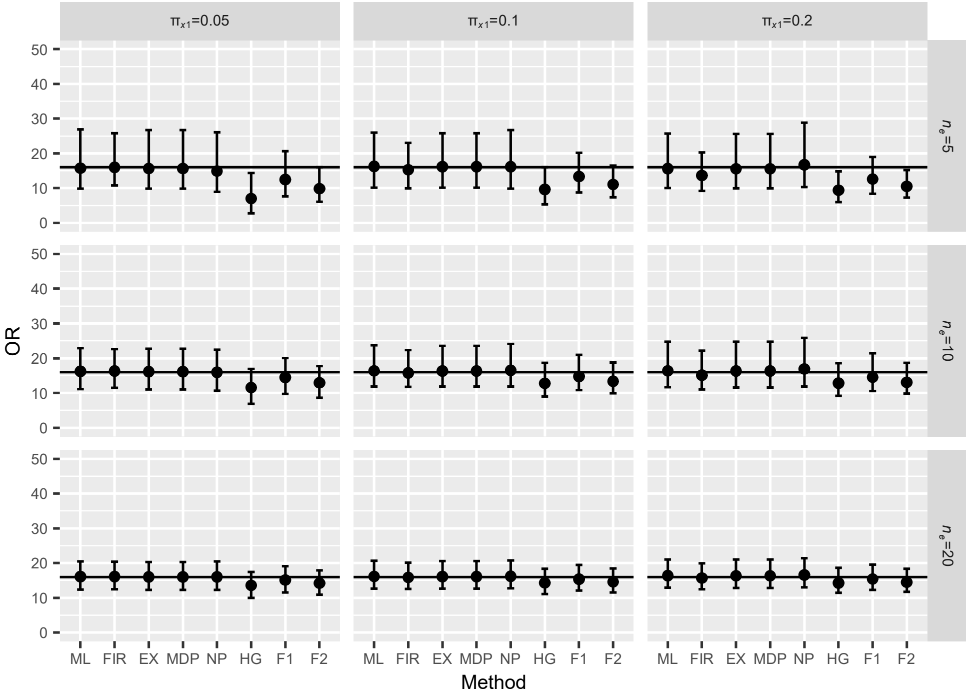

eFigure 12. Distribution of simulated OR under true OR = 16 in scenario 2 (top, n = 1000; bottom, n = 3000). The square represents the median and the error bar represents quartiles 1 and 3. The solid horizontal line is the true OR value. EX: exact method; F1: Bayesian data augmentation with log $F(1, 1)$; F2: Bayesian data augmentation with log $F(2, 2)$; FIR: Firth's method; HG: Bayesian method with hyper-g prior; MDP: mid P-type exact method; ML: ML method; NP: Bayesian method with $N(0, 100)$ prior.

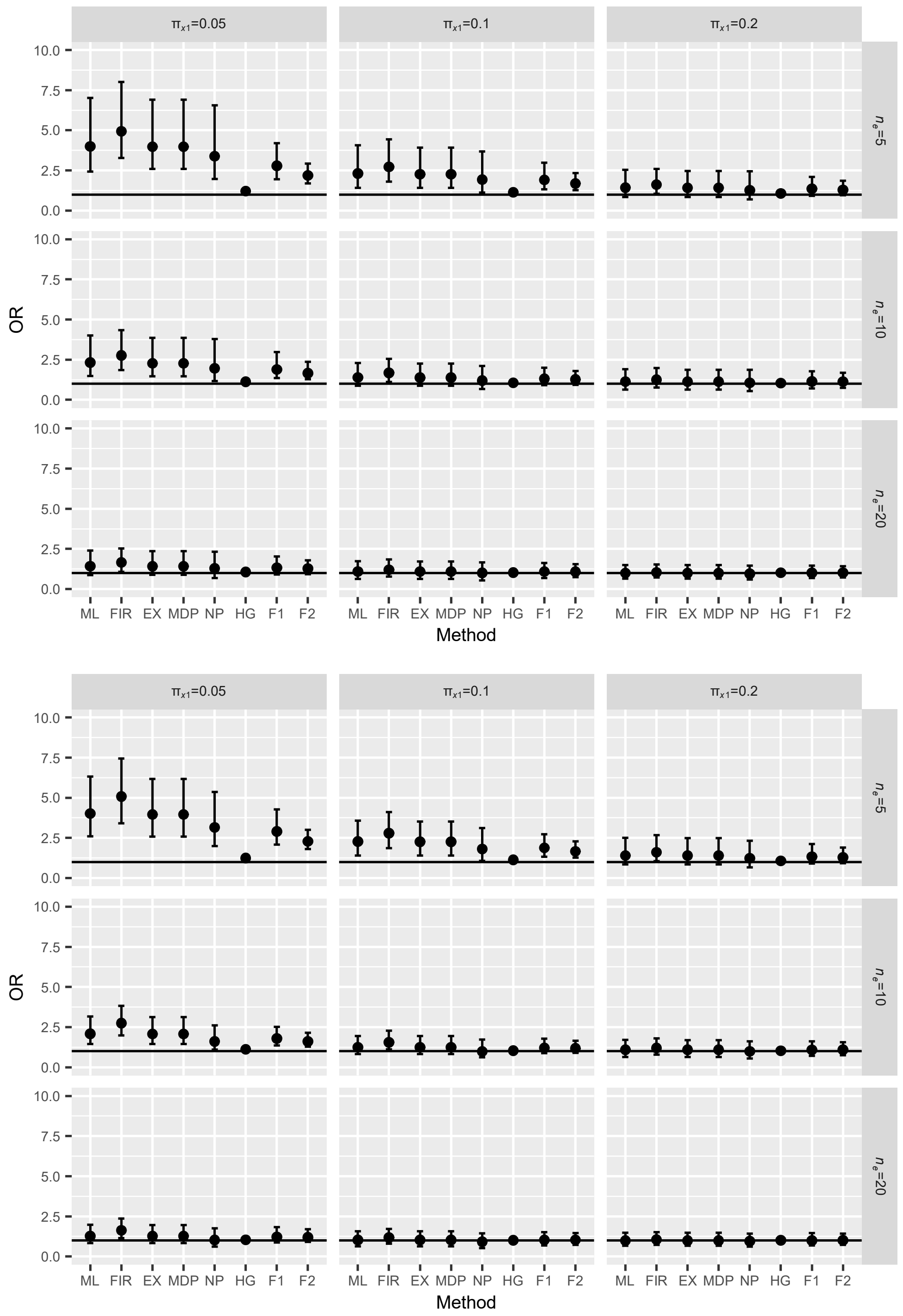

eFigure 13. Distribution of simulated OR under true OR = 1 in scenario 3 (top, n = 100; bottom, n = 300). The square represents the median and the error bar represents quartiles 1 and 3. The solid horizontal line is the true OR value. EX: exact method; F1: Bayesian data augmentation with log $F(1, 1)$; F2: Bayesian data augmentation with log $F(2, 2)$; FIR: Firth's method; HG: Bayesian method with hyper-g prior; MDP: mid P-type exact method; ML: ML method; NP: Bayesian method with $N(0, 100)$ prior.

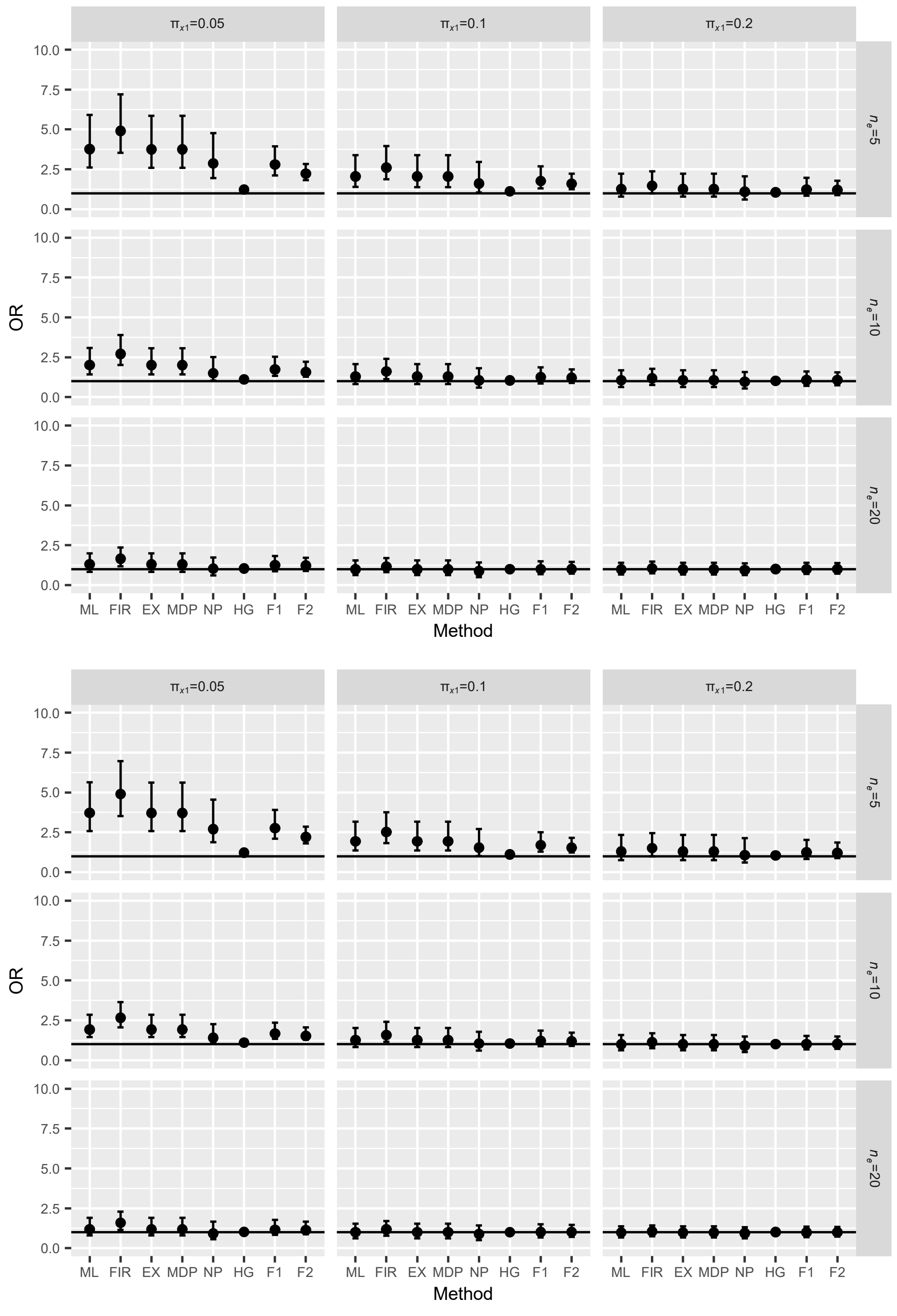

eFigure 14. Distribution of simulated OR under true OR = 1 in scenario 3 (top, n = 1000; bottom, n = 3000). The square represents the median and the error bar represents quartiles 1 and 3. The solid horizontal line is the true OR value. EX: exact method; F1: Bayesian data augmentation with log $F(1, 1)$; F2: Bayesian data augmentation with log $F(2, 2)$; FIR: Firth's method; HG: Bayesian method with hyper-g prior; MDP: mid P-type exact method; ML: ML method; NP: Bayesian method with $N(0, 100)$ prior.

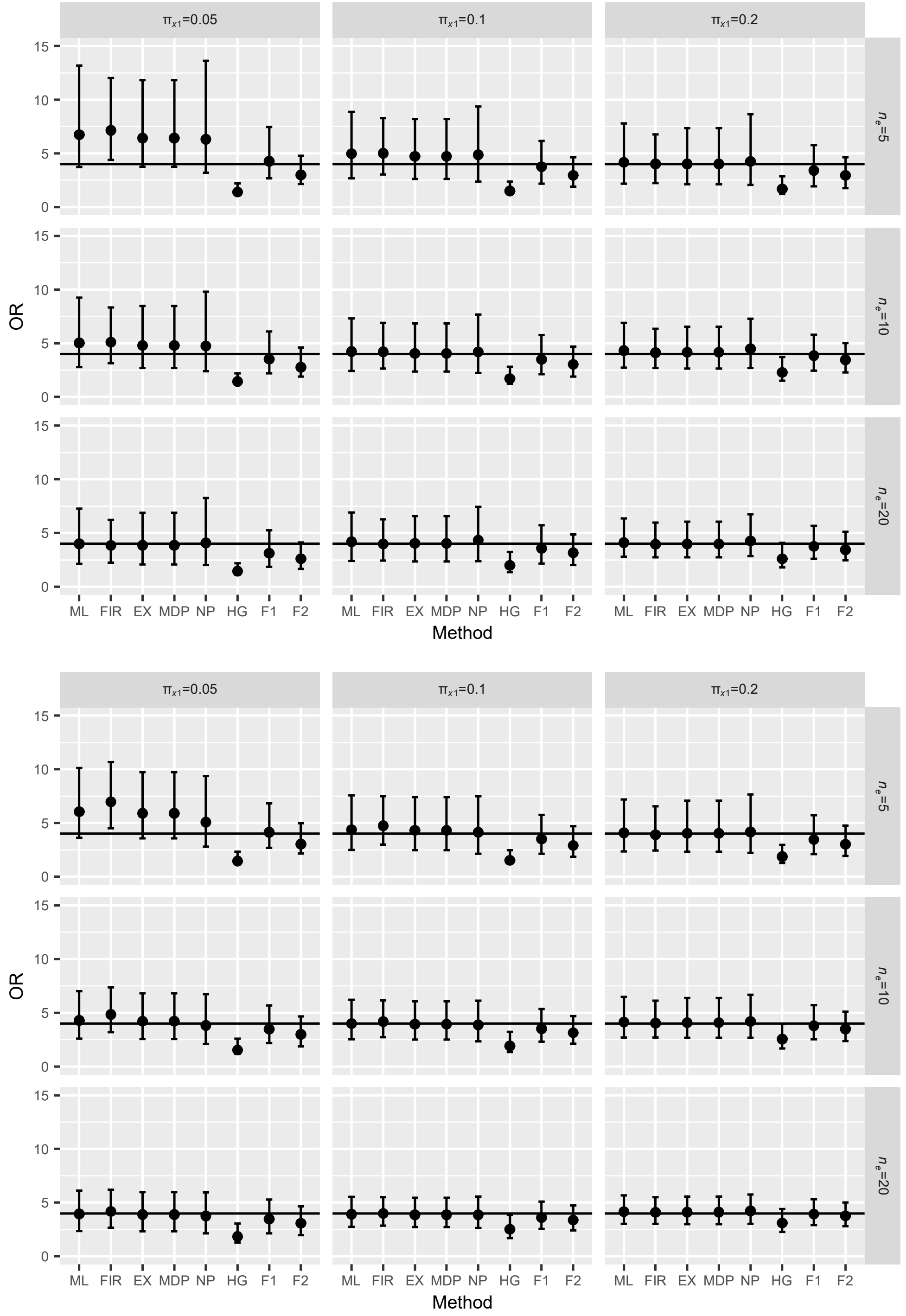

eFigure 15. Distribution of simulated OR under true OR = 4 in scenario 3 (top, n = 100; bottom, n = 300). The square represents the median and the error bar represents quartiles 1 and 3. The solid horizontal line is the true OR value. EX: exact method; F1: Bayesian data augmentation with log F(1, 1); F2: Bayesian data augmentation with log F(2, 2); FIR: Firth's method; HG: Bayesian method with hyper-g prior; MDP: mid P-type exact method; ML: ML method; NP: Bayesian method with N(0, 100) prior.

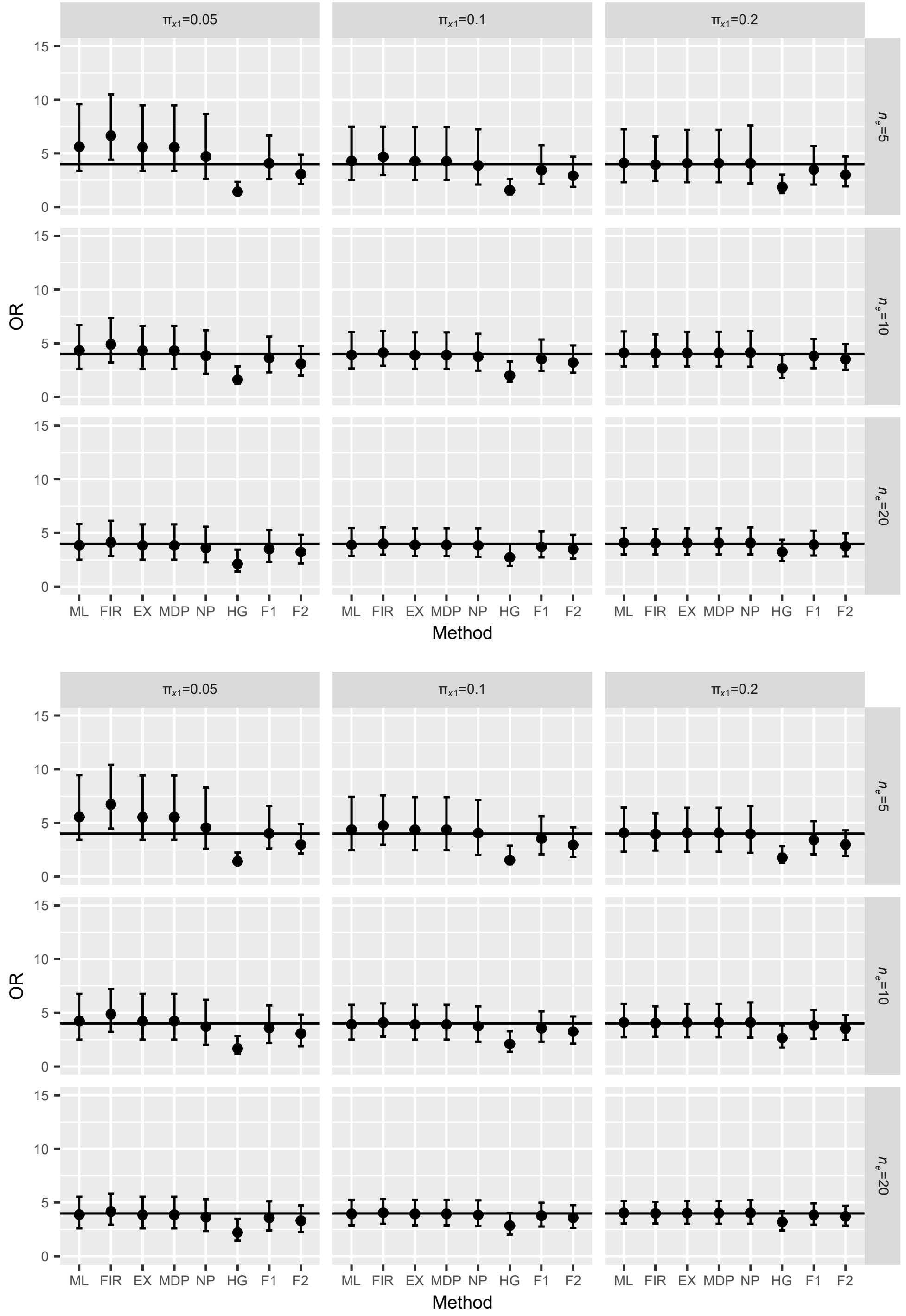

eFigure 16. Distribution of simulated OR under true OR = 4 in scenario 3 (top, n = 1000; bottom, n = 3000). The square represents the median and the error bar represents quartiles 1 and 3. The solid horizontal line is the true OR value. EX: exact method; F1: Bayesian data augmentation with log $F(1, 1)$; F2: Bayesian data augmentation with log $F(2, 2)$; FIR: Firth's method; HG: Bayesian method with hyper-g prior; MDP: mid P-type exact method; ML: ML method; NP: Bayesian method with $N(0, 100)$ prior.

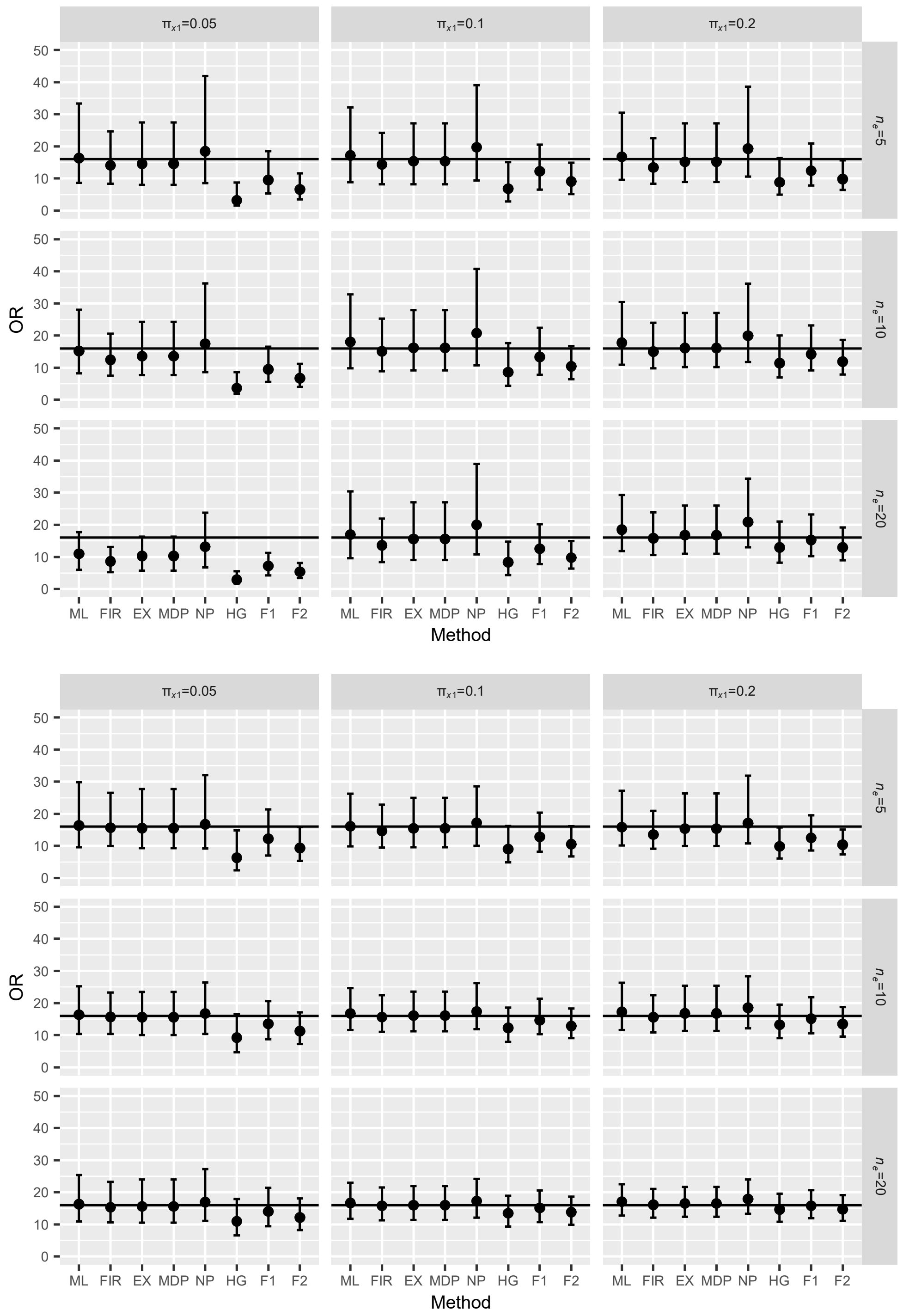

eFigure 17. Distribution of simulated OR under true OR = 16 in scenario 3 (top, n = 100; bottom, n = 300). The square represents the median and the error bar represents quartiles 1 and 3. The solid horizontal line is the true OR value. EX: exact method; F1: Bayesian data augmentation with log $F(1, 1)$; F2: Bayesian data augmentation with log $F(2, 2)$; FIR: Firth's method; HG: Bayesian method with hyper-g prior; MDP: mid P-type exact method; ML: ML method; NP: Bayesian method with $N(0, 100)$ prior.

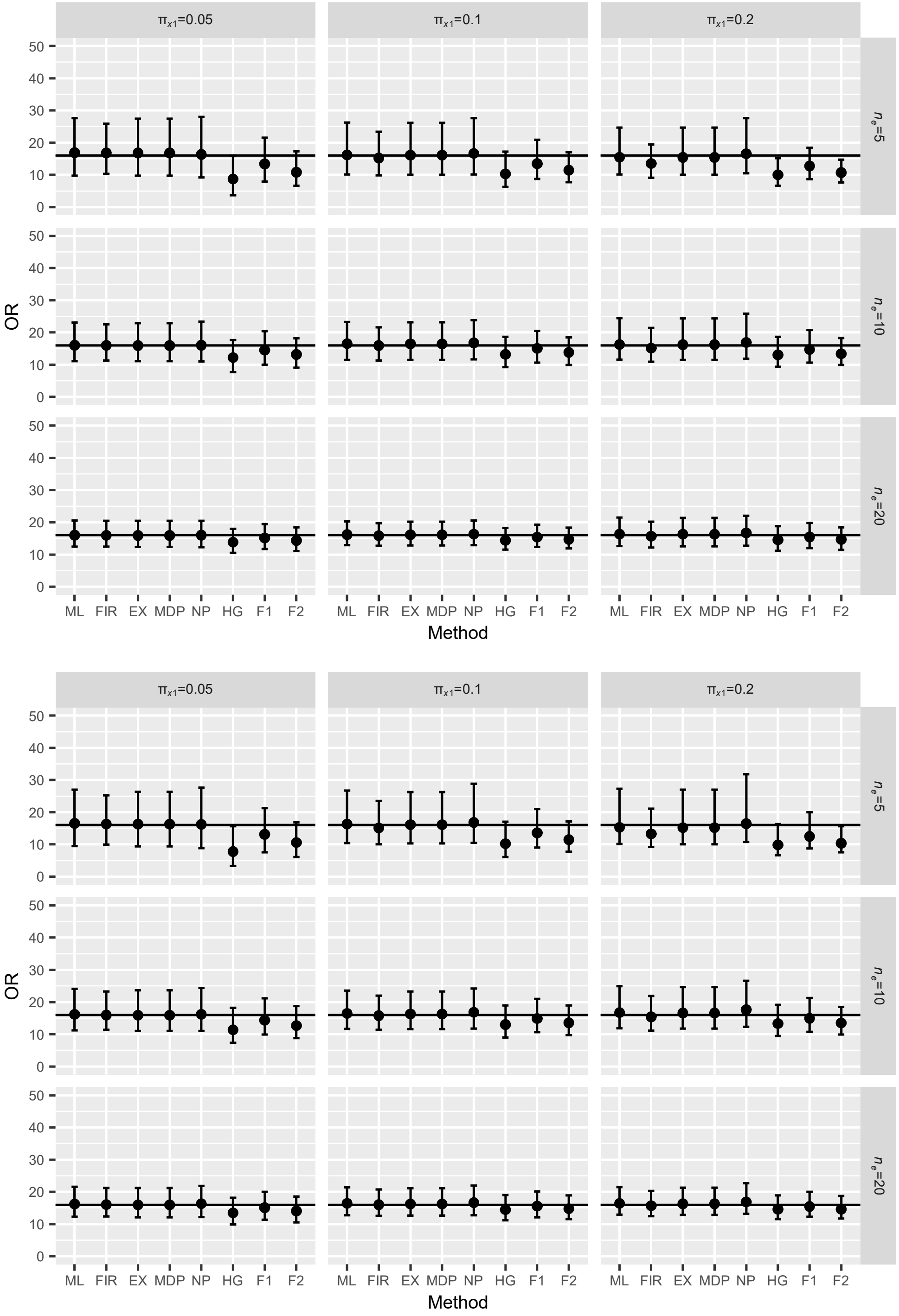

eFigure 18. Distribution of simulated OR under true OR = 16 in scenario 3 (top, n = 1000; bottom, n = 3000). The square represents the median and the error bar represents quartiles 1 and 3. The solid horizontal line is the true OR value. EX: exact method; F1: Bayesian data augmentation with log F(1, 1); F2: Bayesian data augmentation with log F(2, 2); FIR: Firth's method; HG: Bayesian method with hyper-g prior; MDP: mid P-type exact method; ML: ML method; NP: Bayesian method with N(0, 100) prior.

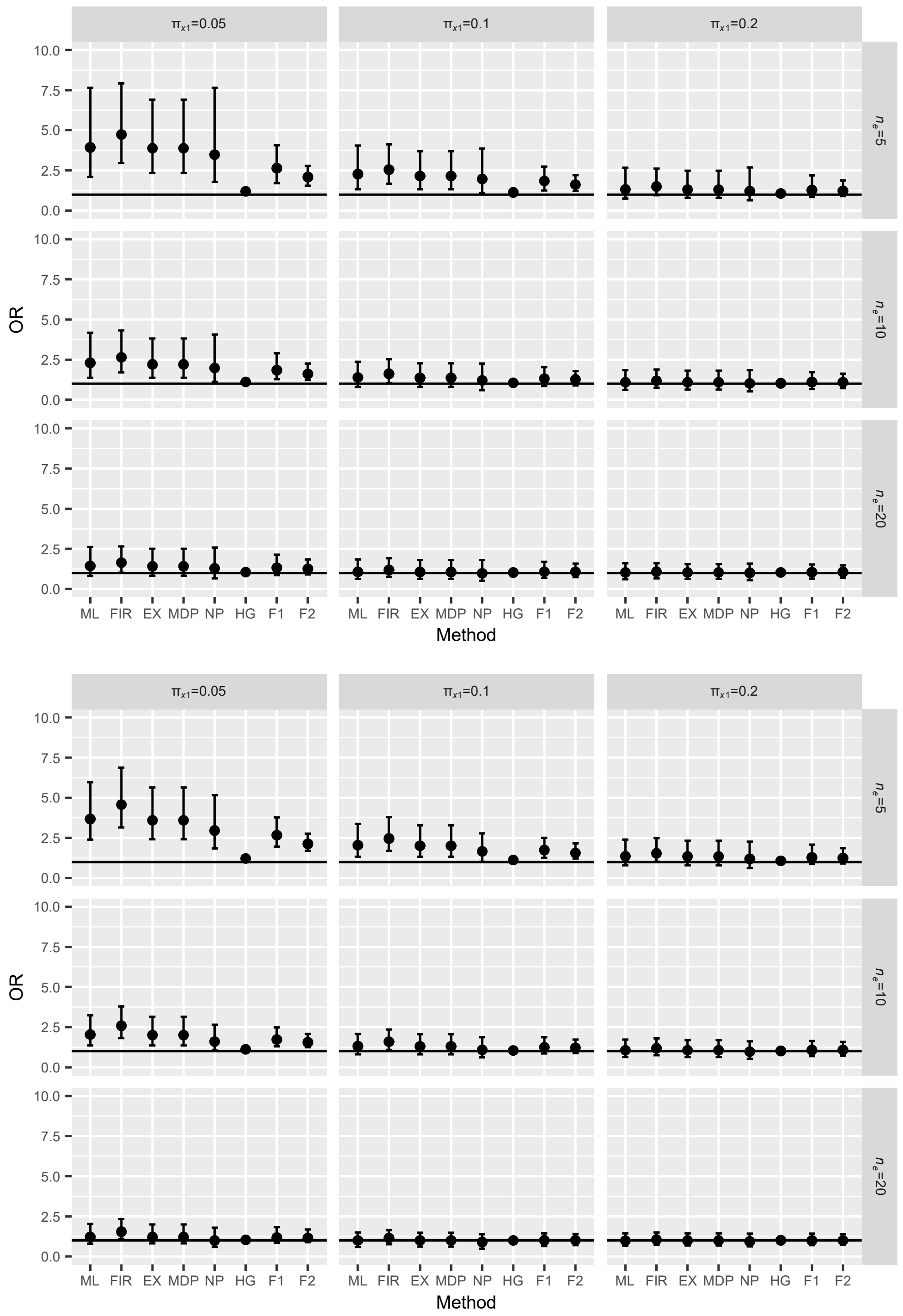

eFigure 19. Distribution of simulated OR under true OR = 1 in scenario 4 (top, n = 100; bottom, n = 300). The square represents the median and the error bar represents quartiles 1 and 3. The solid horizontal line is the true OR value. EX: exact method; F1: Bayesian data augmentation with log F(1, 1); F2: Bayesian data augmentation with log F(2, 2); FIR: Firth's method; HG: Bayesian method with hyper-g prior; MDP: mid P-type exact method; ML: ML method; NP: Bayesian method with N(0, 100) prior.

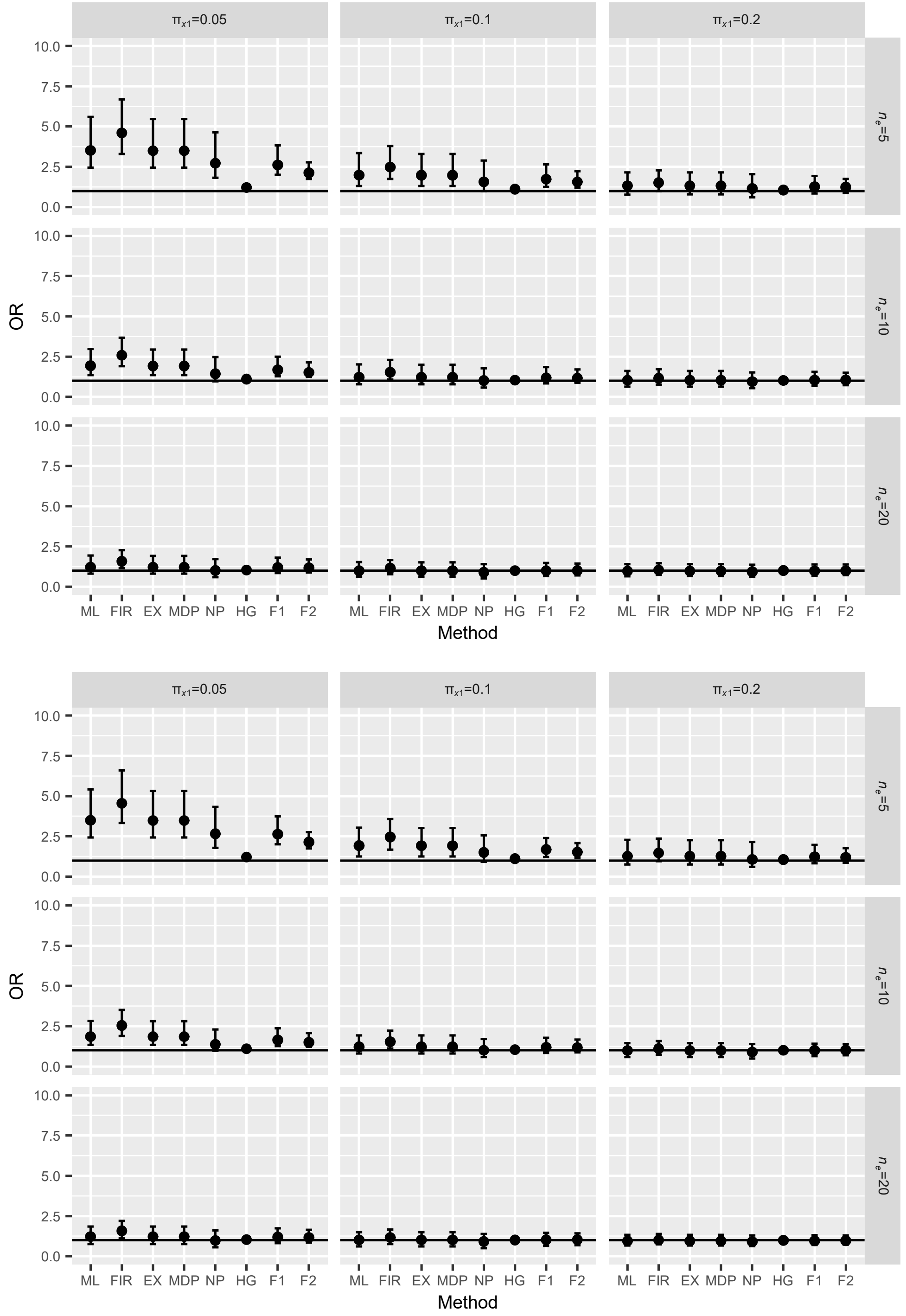

eFigure 20. Distribution of simulated OR under true OR = 1 in scenario 4 (top, n = 1000; bottom, n = 3000). The square represents the median and the error bar represents quartiles 1 and 3. The solid horizontal line is the true OR value. EX: exact method; F1: Bayesian data augmentation with log $F(1, 1)$; F2: Bayesian data augmentation with log $F(2, 2)$; FIR: Firth's method; HG: Bayesian method with hyper-g prior; MDP: mid P-type exact method; ML: ML method; NP: Bayesian method with $N(0, 100)$ prior.

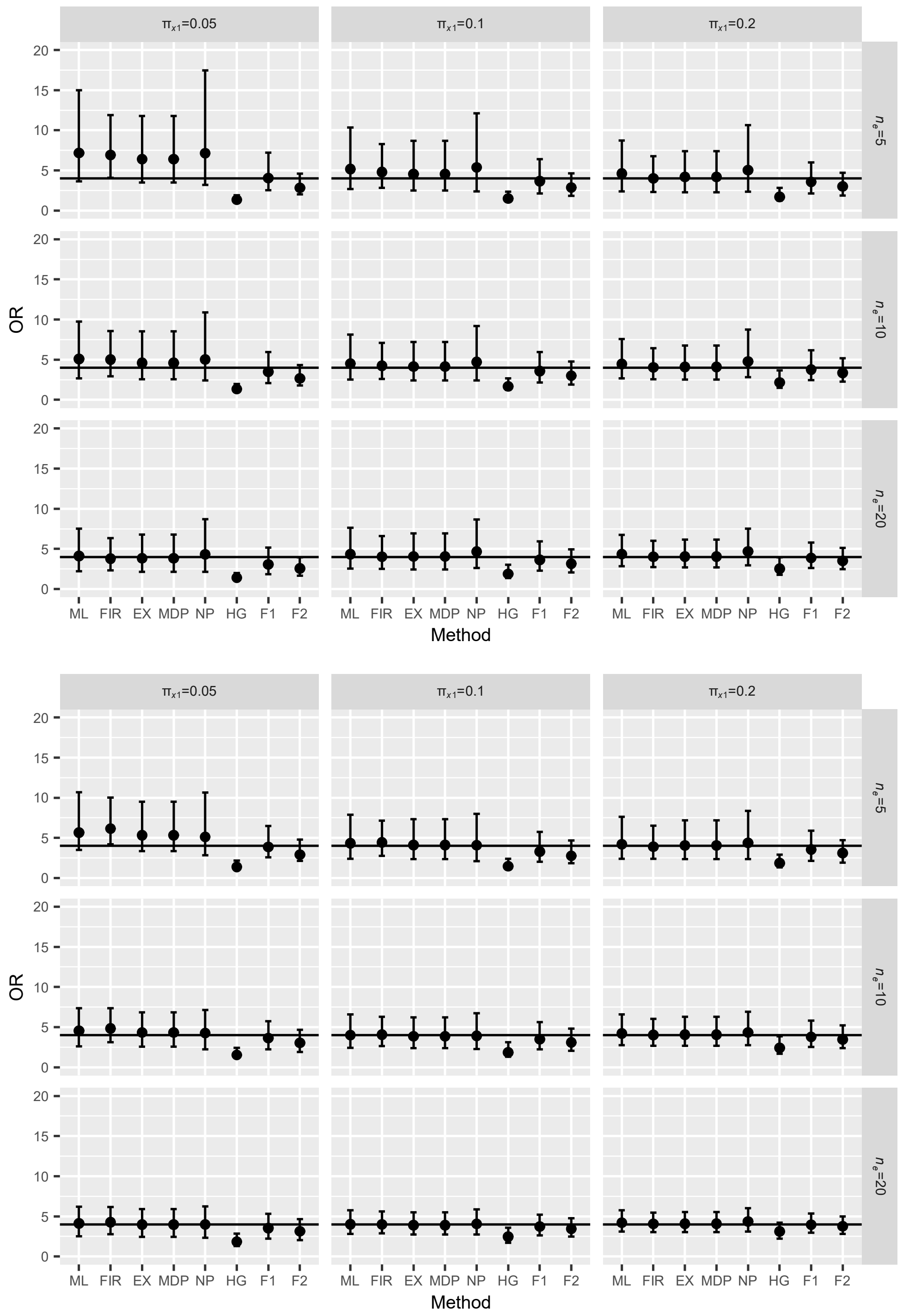

eFigure 21. Distribution of simulated OR under true OR = 4 in scenario 4 (top, n = 100; bottom, n = 300). The square represents the median and the error bar represents quartiles 1 and 3. The solid horizontal line is the true OR value. EX: exact method; F1: Bayesian data augmentation with log $F(1, 1)$; F2: Bayesian data augmentation with log $F(2, 2)$; FIR: Firth's method; HG: Bayesian method with hyper-g prior; MDP: mid P-type exact method; ML: ML method; NP: Bayesian method with $N(0, 100)$ prior.

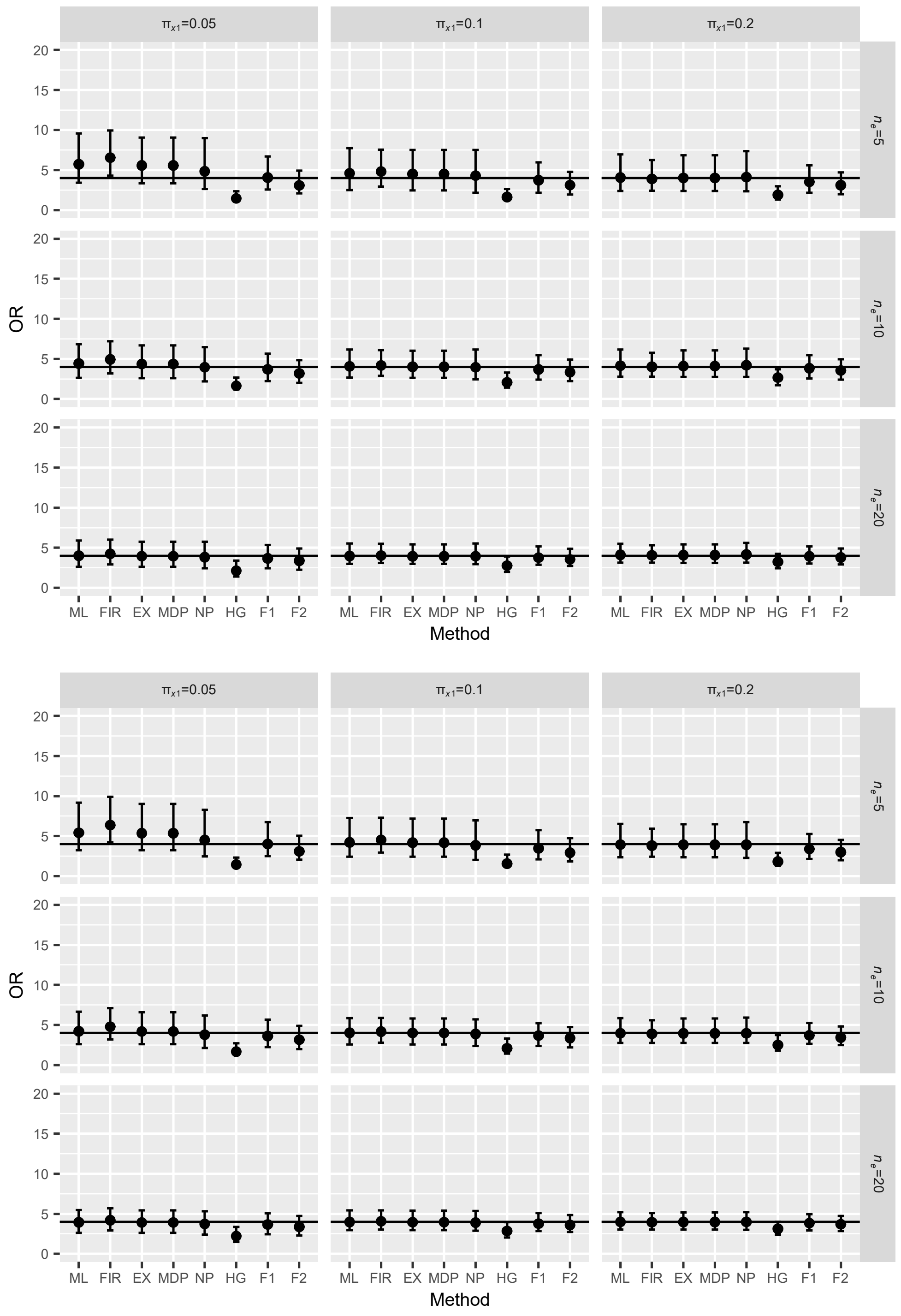

eFigure 22. Distribution of simulated OR under true OR = 4 in scenario 4 (top, n = 1000; bottom, n = 3000). The square represents the median and the error bar represents quartiles 1 and 3. The solid horizontal line is the true OR value. EX: exact method; F1: Bayesian data augmentation with log $F(1, 1)$; F2: Bayesian data augmentation with log $F(2, 2)$; FIR: Firth's method; HG: Bayesian method with hyper-g prior; MDP: mid P-type exact method; ML: ML method; NP: Bayesian method with $N(0, 100)$ prior.

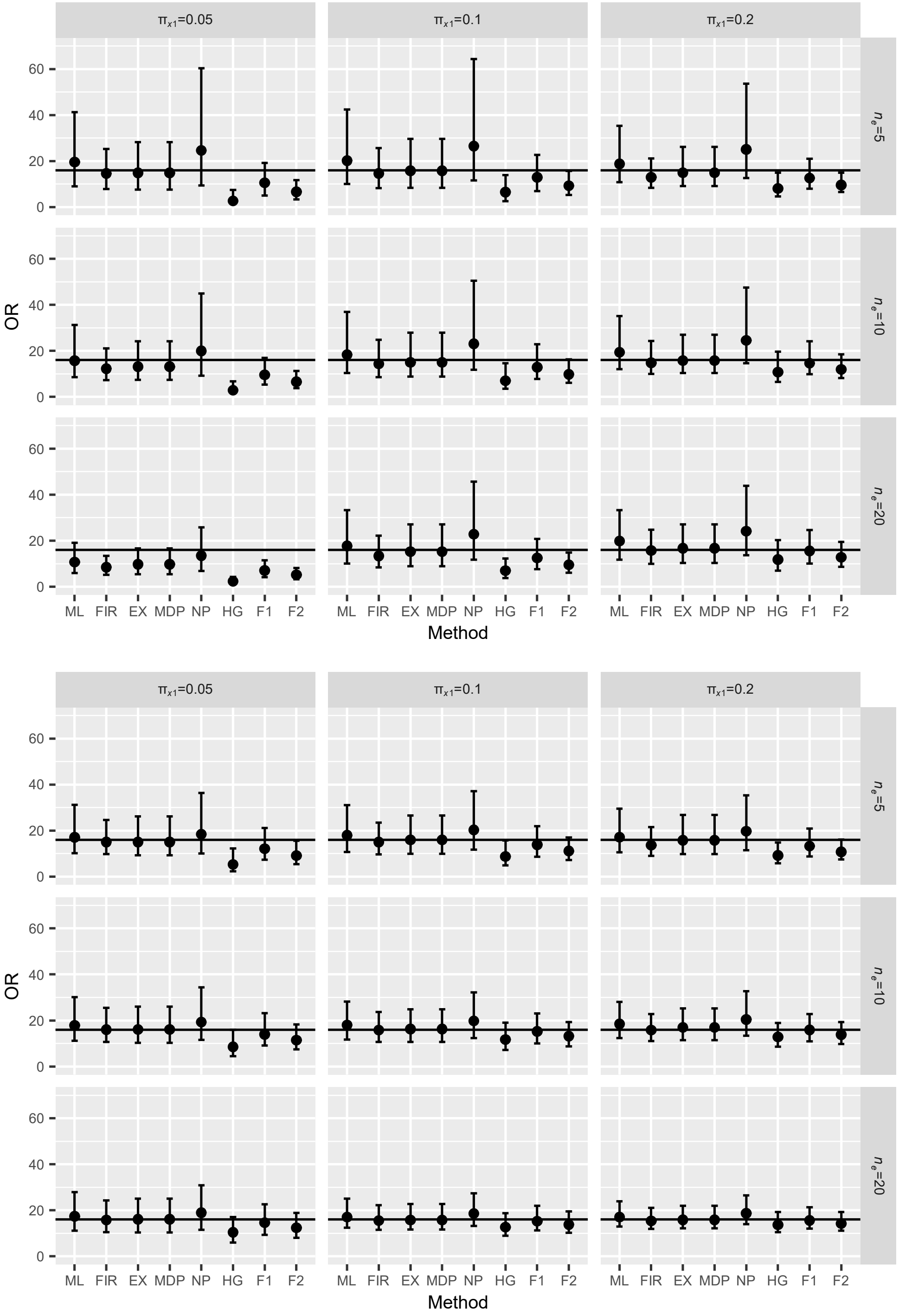

eFigure 23. Distribution of simulated OR under true OR = 16 in scenario 4 (top, n = 100; bottom, n = 300). The square represents the median and the error bar represents quartiles 1 and 3. The solid horizontal line is the true OR value. EX: exact method; F1: Bayesian data augmentation with log F(1, 1); F2: Bayesian data augmentation with log F(2, 2); FIR: Firth's method; HG: Bayesian method with hyper-g prior; MDP: mid P-type exact method; ML: ML method; NP: Bayesian method with N(0, 100) prior.

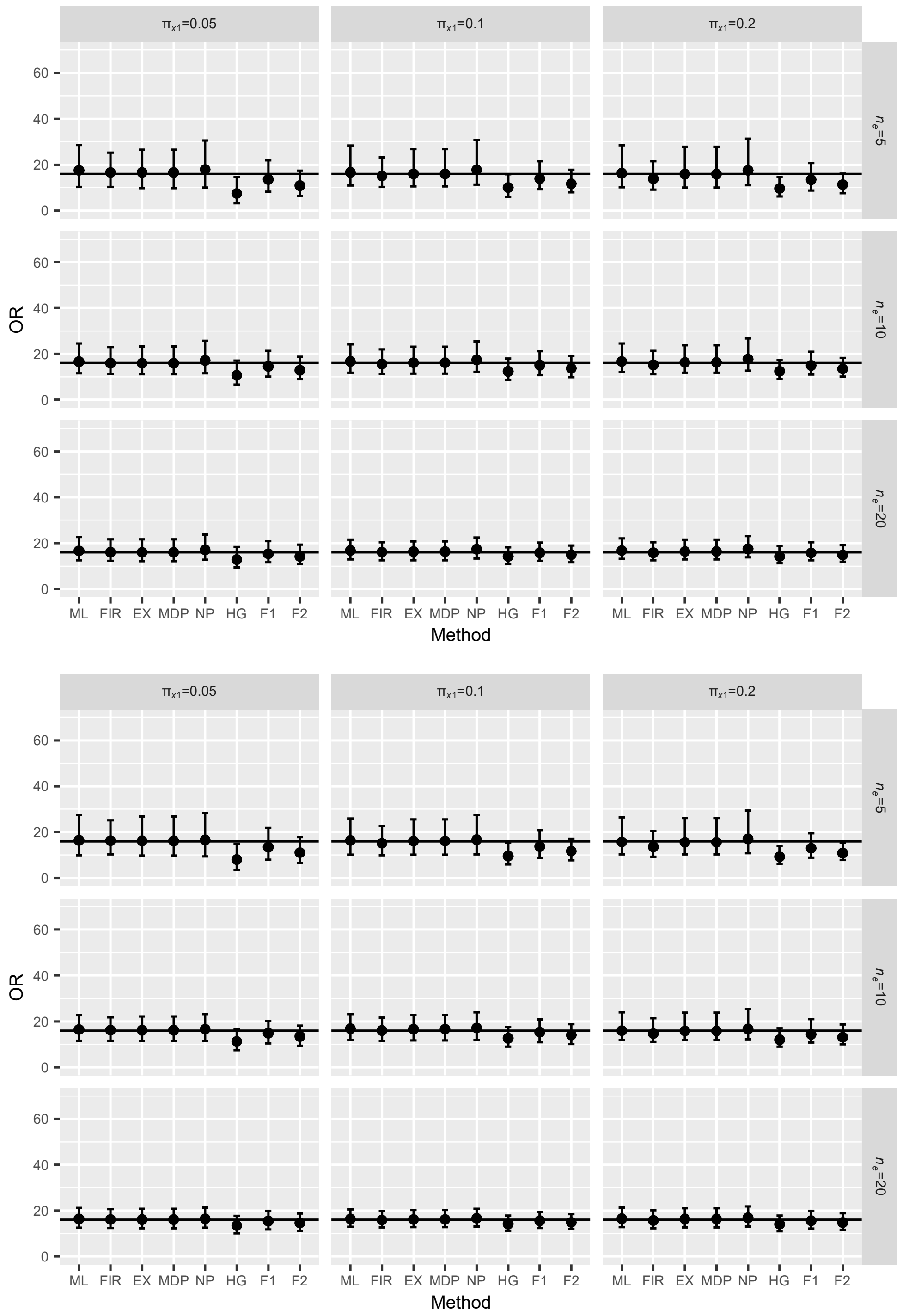

eFigure 24. Distribution of simulated OR under true OR = 16 in scenario 4 (top, n = 1000; bottom, n = 3000). The square represents the median and the error bar represents quartiles 1 and 3. The solid horizontal line is the true OR value. EX: exact method; F1: Bayesian data augmentation with log $F(1, 1)$; F2: Bayesian data augmentation with log $F(2, 2)$; FIR: Firth's method; HG: Bayesian method with hyper-g prior; MDP: mid P-type exact method; ML: ML method; NP: Bayesian method with $N(0, 100)$ prior.

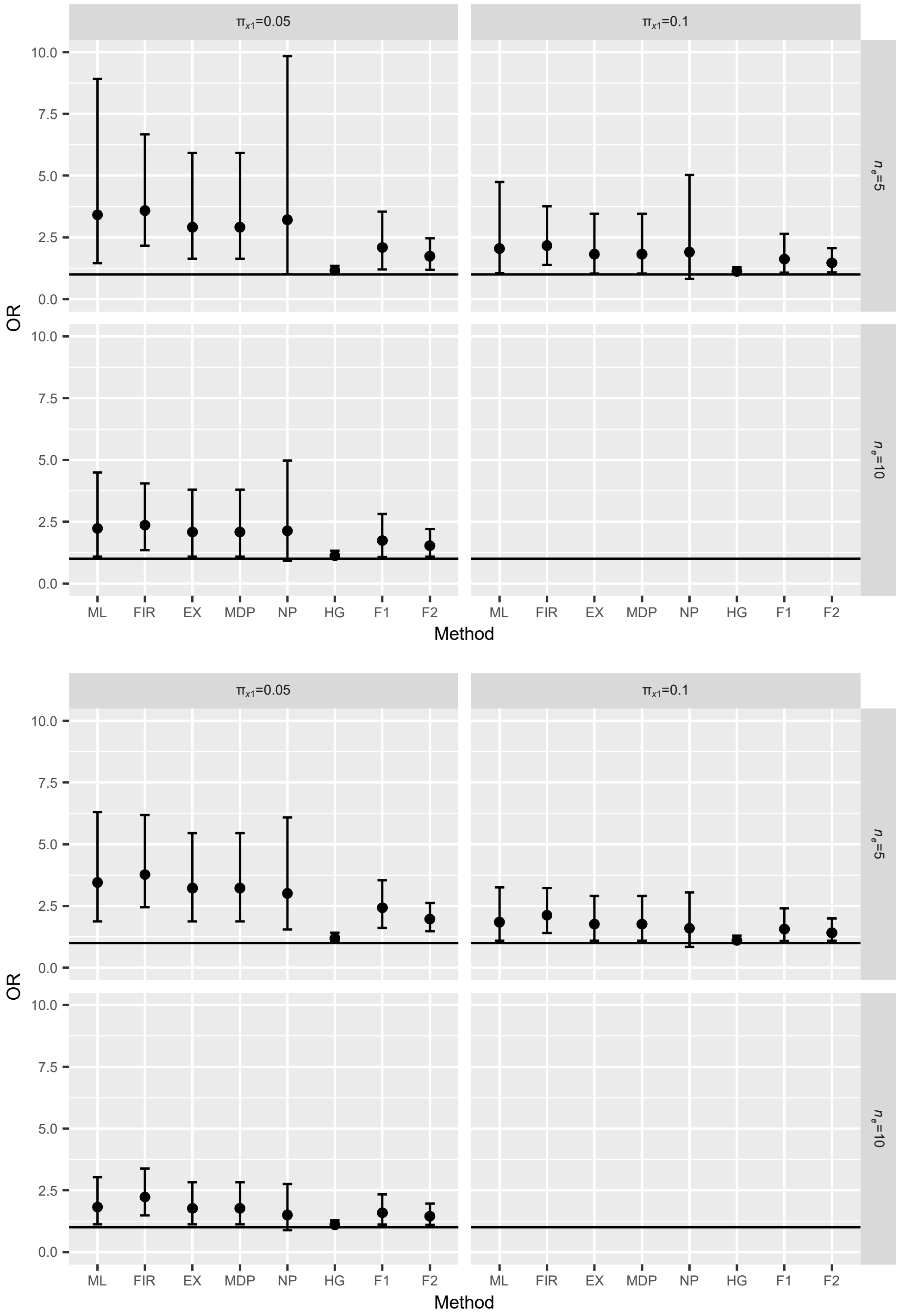

eFigure 25. Distribution of simulated OR under true OR = 1 in scenario 5 (top, n = 100; bottom, n = 300). The square represents the median and the error bar represents quartiles 1 and 3. The solid horizontal line is the true OR value. EX: exact method; F1: Bayesian data augmentation with log F(1, 1); F2: Bayesian data augmentation with log F(2, 2); FIR: Firth's method; HG: Bayesian method with hyper-g prior; MDP: mid P-type exact method; ML: ML method; NP: Bayesian method with N(0, 100) prior.

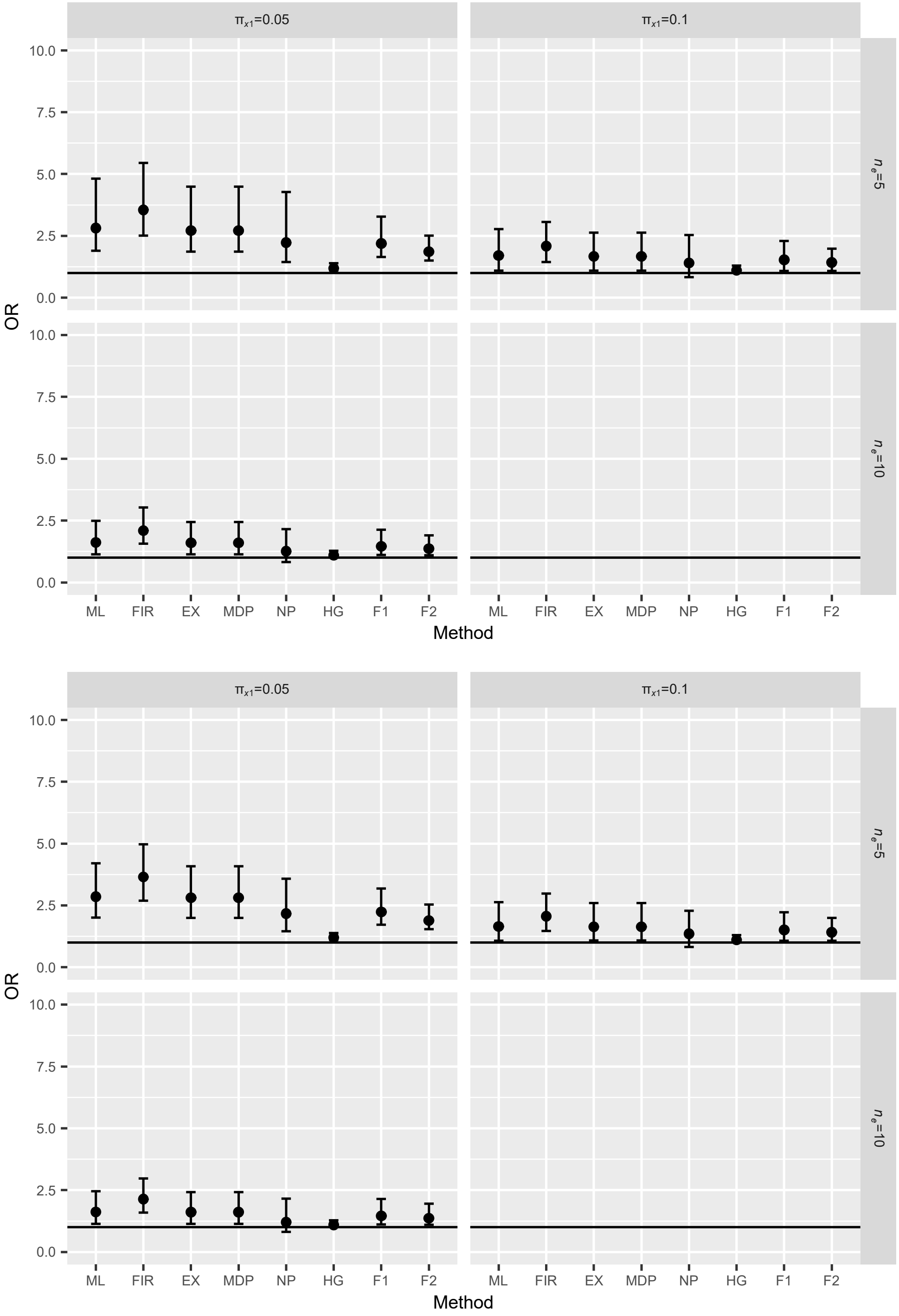

eFigure 26. Distribution of simulated OR under true OR = 1 in scenario 5 (top, n = 1000; bottom, n = 3000). The square represents the median and the error bar represents quartiles 1 and 3. The solid horizontal line is the true OR value. EX: exact method; F1: Bayesian data augmentation with log $F(1, 1)$; F2: Bayesian data augmentation with log $F(2, 2)$; FIR: Firth's method; HG: Bayesian method with hyper-g prior; MDP: mid P-type exact method; ML: ML method; NP: Bayesian method with $N(0, 100)$ prior.

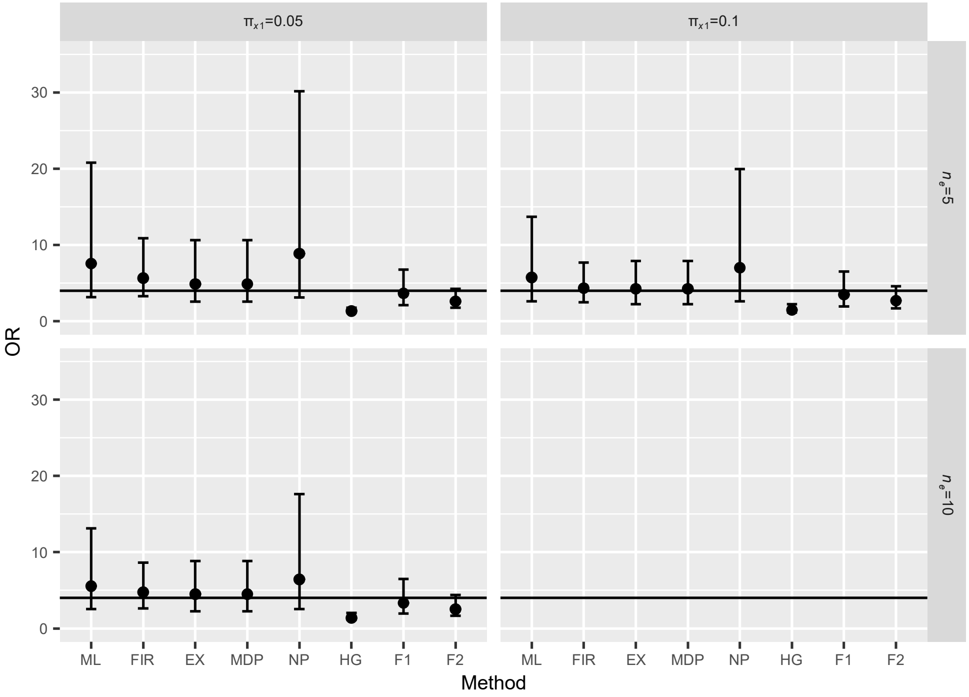

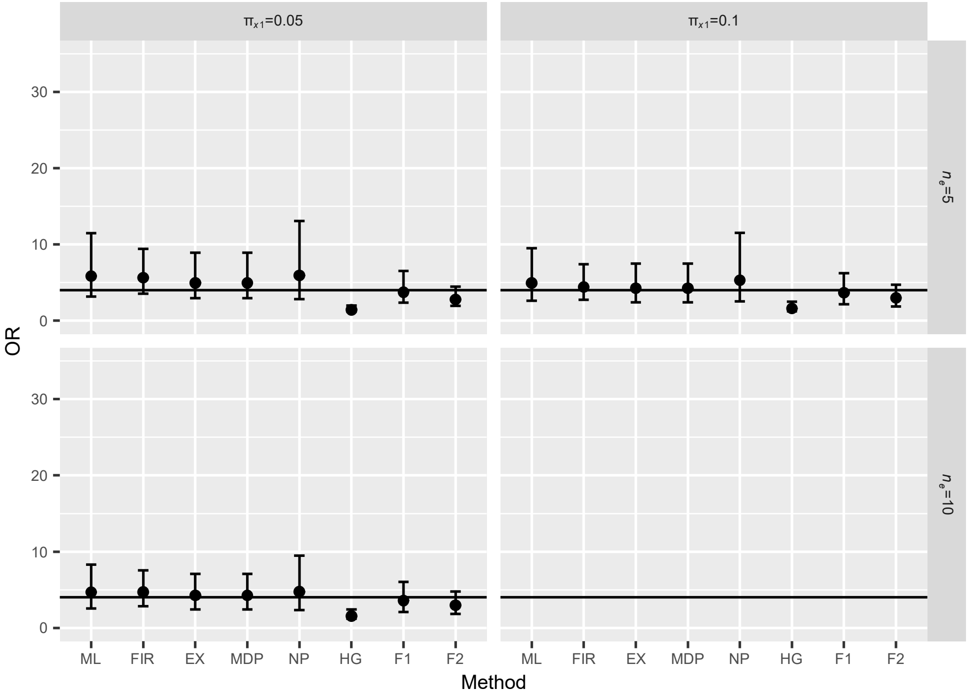

eFigure 27. Distribution of simulated OR under true OR = 4 in scenario 5 (top, n = 100; bottom, n = 300). The square represents the median and the error bar represents quartiles 1 and 3. The solid horizontal line is the true OR value. EX: exact method; F1: Bayesian data augmentation with log F(1, 1); F2: Bayesian data augmentation with log F(2, 2); FIR: Firth's method; HG: Bayesian method with hyper-g prior; MDP: mid P-type exact method; ML: ML method; NP: Bayesian method with N(0, 100) prior.

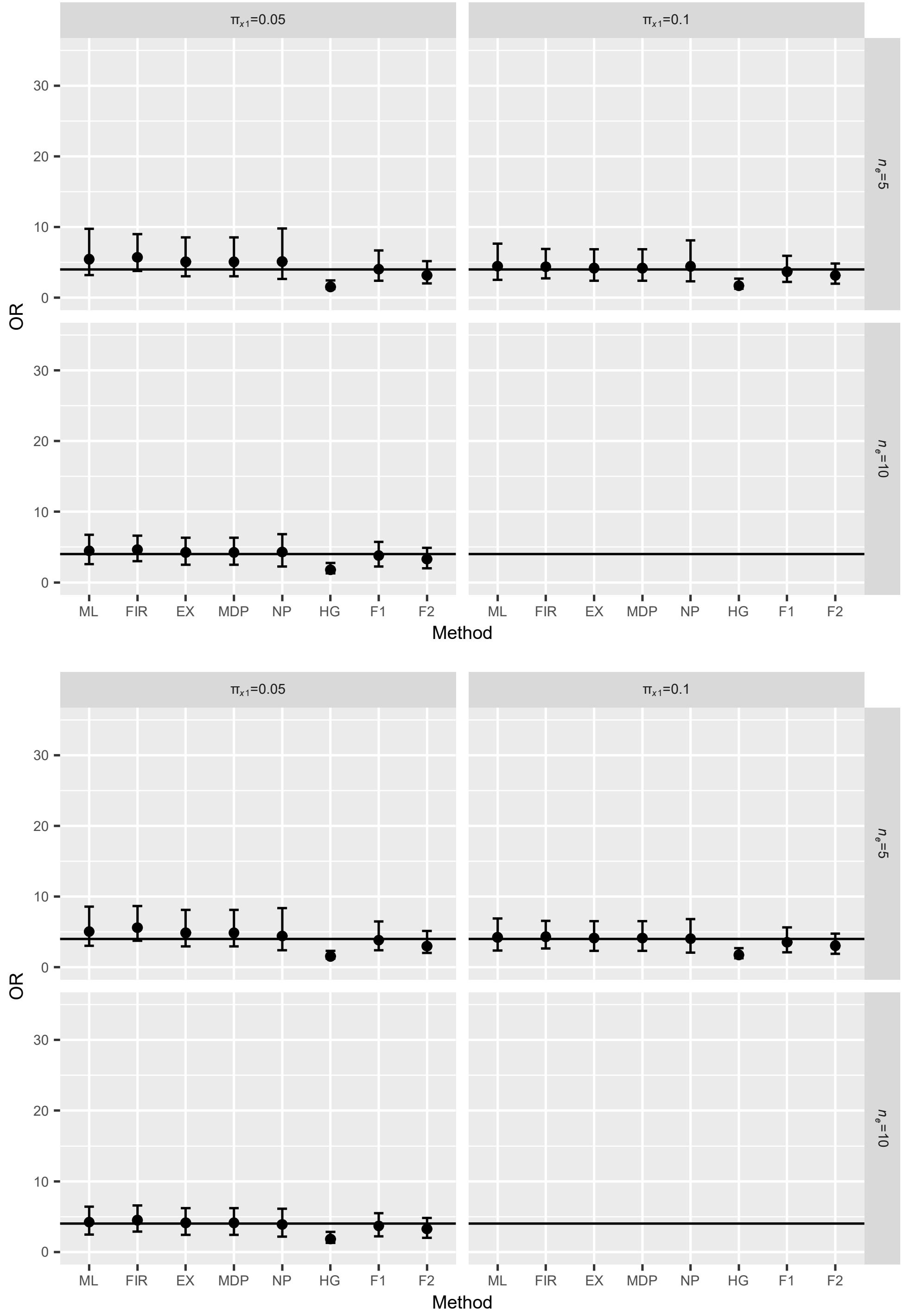

eFigure 28. Distribution of simulated OR under true OR = 4 in scenario 5 (top, n = 1000; bottom, n = 3000). The square represents the median and the error bar represents quartiles 1 and 3. The solid horizontal line is the true OR value. EX: exact method; F1: Bayesian data augmentation with log F(1, 1); F2: Bayesian data augmentation with log F(2, 2); FIR: Firth's method; HG: Bayesian method with hyper-g prior; MDP: mid P-type exact method; ML: ML method; NP: Bayesian method with N(0, 100) prior.

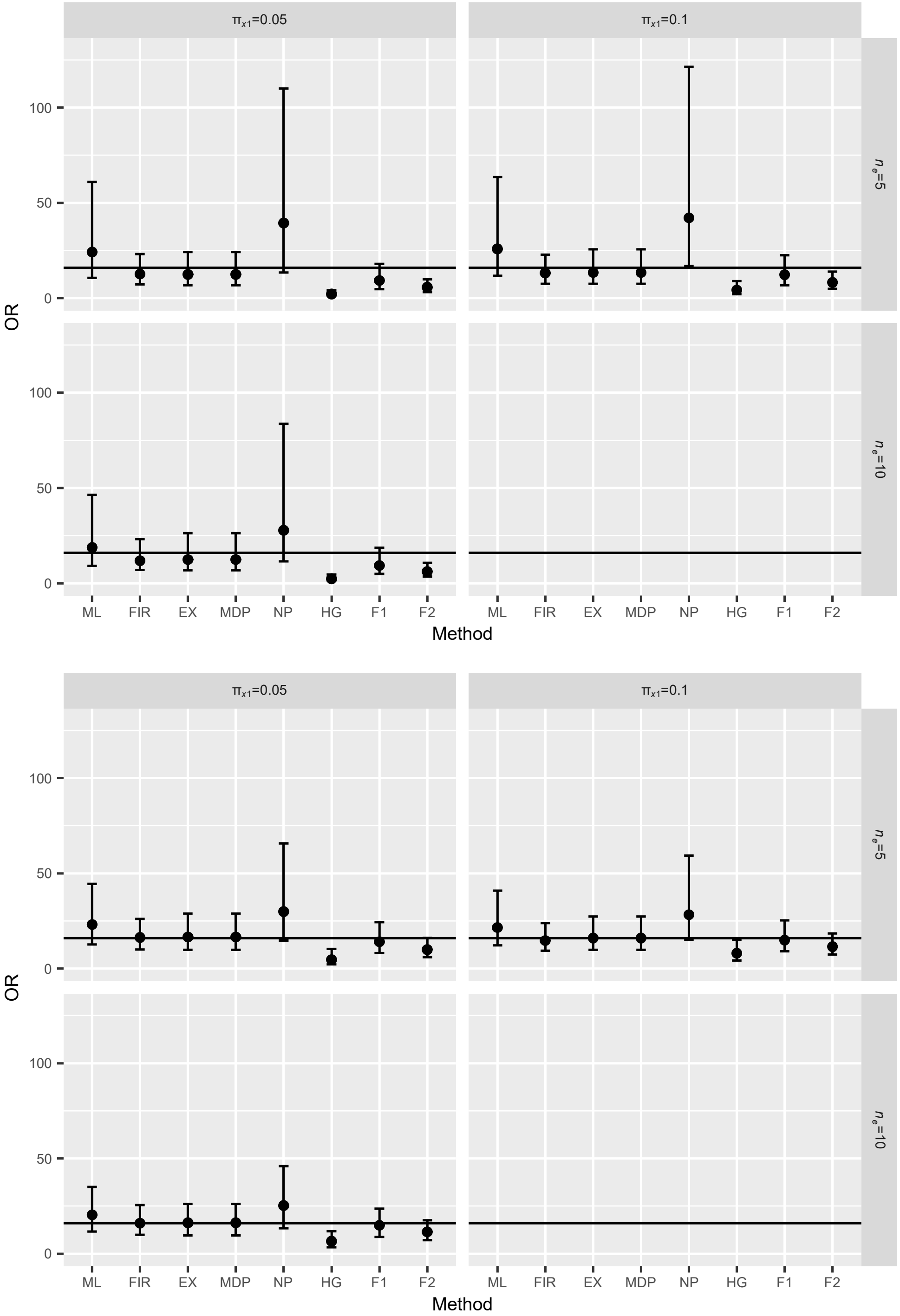

eFigure 29. Distribution of simulated OR under true OR = 16 in scenario 5 (top, n = 100; bottom, n = 300). The square represents the median and the error bar represents quartiles 1 and 3. The solid horizontal line is the true OR value. EX: exact method; F1: Bayesian data augmentation with log F(1, 1); F2: Bayesian data augmentation with log F(2, 2); FIR: Firth's method; HG: Bayesian method with hyper-g prior; MDP: mid P-type exact method; ML: ML method; NP: Bayesian method with N(0, 100) prior.

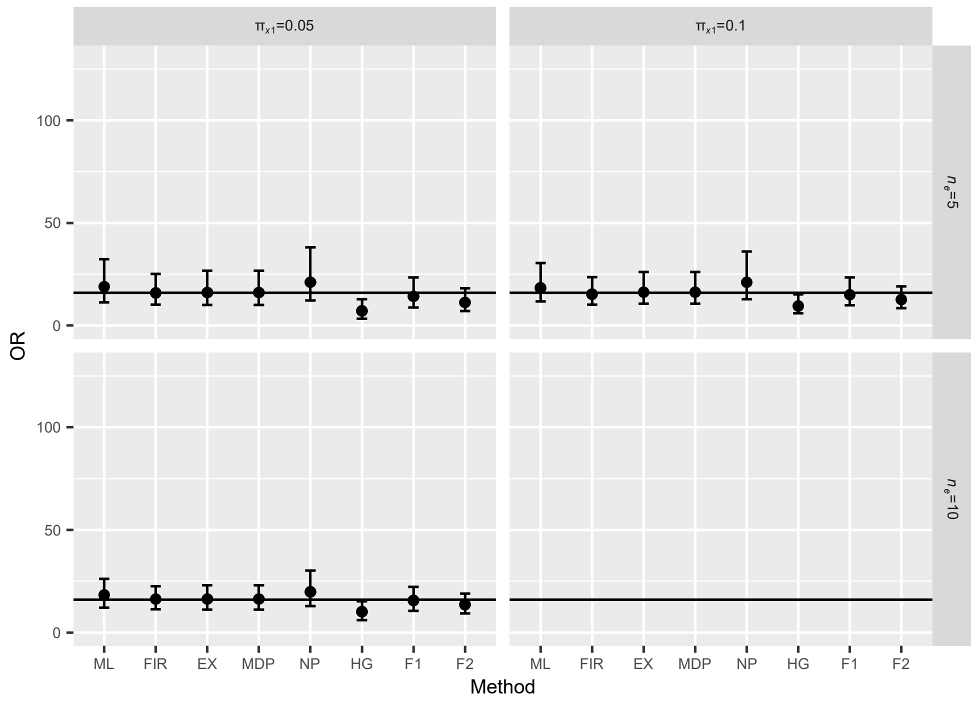

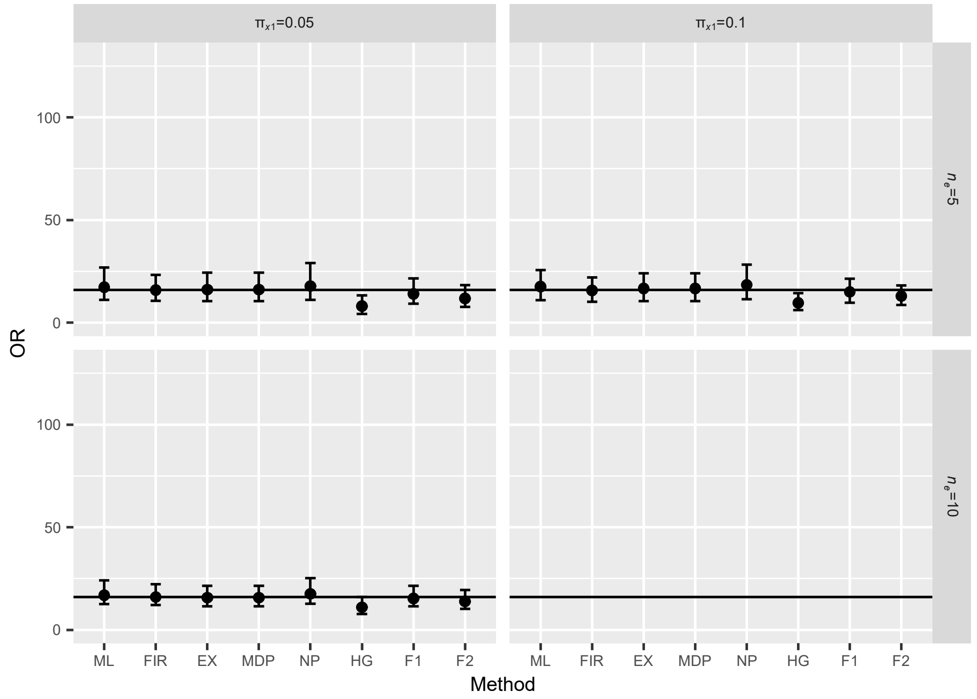

eFigure 30. Distribution of simulated OR under true OR = 16 in scenario 5 (top, n = 1000; bottom, n = 3000). The square represents the median and the error bar represents quartiles 1 and 3. The solid horizontal line is the true OR value. EX: exact method; F1: Bayesian data augmentation with log $F(1, 1)$; F2: Bayesian data augmentation with log $F(2, 2)$; FIR: Firth's method; HG: Bayesian method with hyper-g prior; MDP: mid P-type exact method; ML: ML method; NP: Bayesian method with $N(0, 100)$ prior.

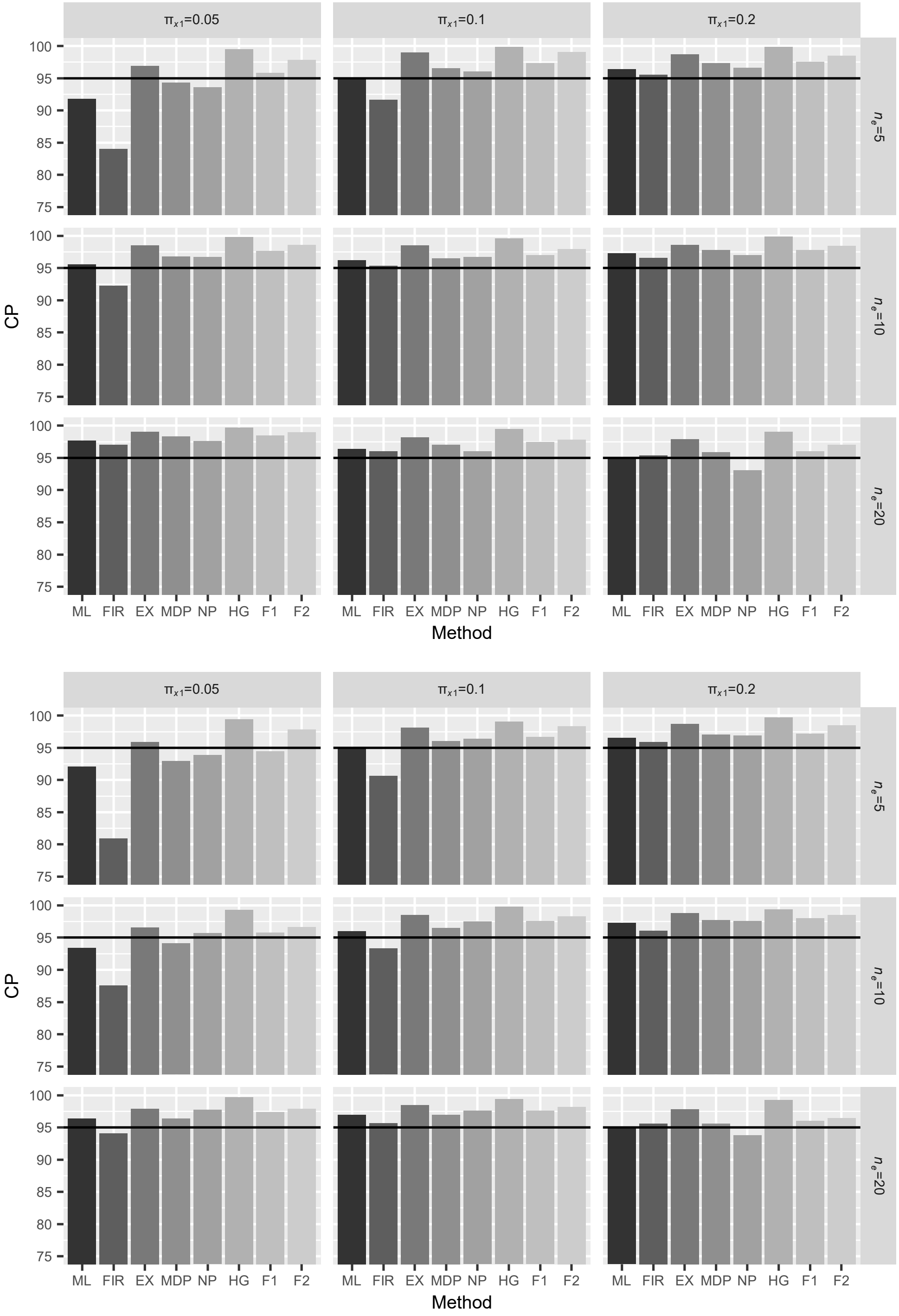

eFigure 31. 95% CI coverage probability under true OR = 1 in scenario 1 (top, n = 100; bottom, n = 1000). EX: exact method; F1: Bayesian data augmentation with log $F(1, 1)$; F2: Bayesian data augmentation with log $F(2, 2)$; FIR: Firth's method; HG: Bayesian method with hyper-g prior; MDP: mid P-type exact method; ML: ML method; NP: Bayesian method with $N(0, 100)$ prior.

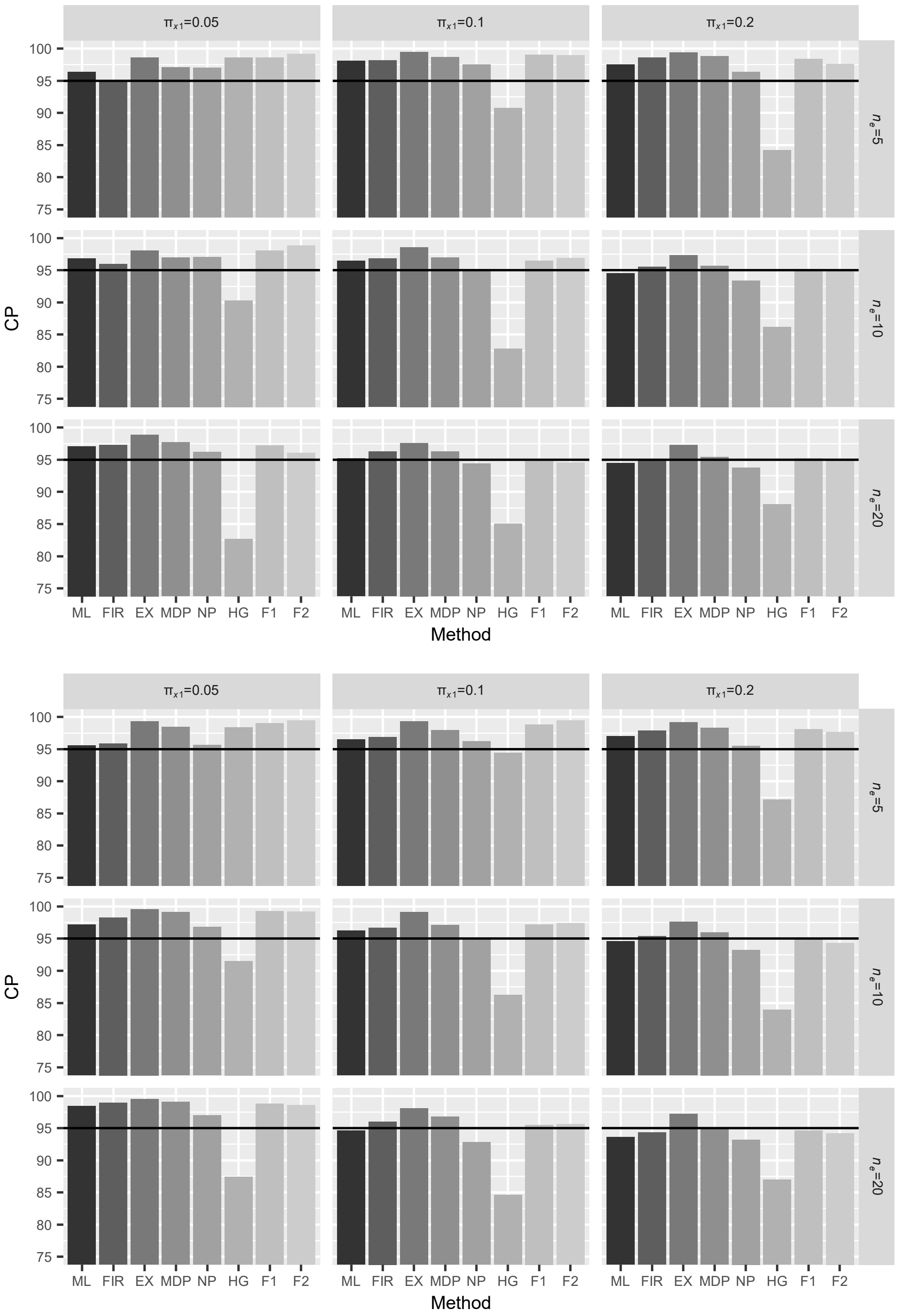

eFigure 32. 95% CI coverage probability under true OR = 4 in scenario 1 (top, n = 100; bottom, n = 1000). EX: exact method; F1: Bayesian data augmentation with log $F(1, 1)$; F2: Bayesian data augmentation with log $F(2, 2)$; FIR: Firth's method; HG: Bayesian method with hyper-g prior; MDP: mid P-type exact method; ML: ML method; NP: Bayesian method with $N(0, 100)$ prior.

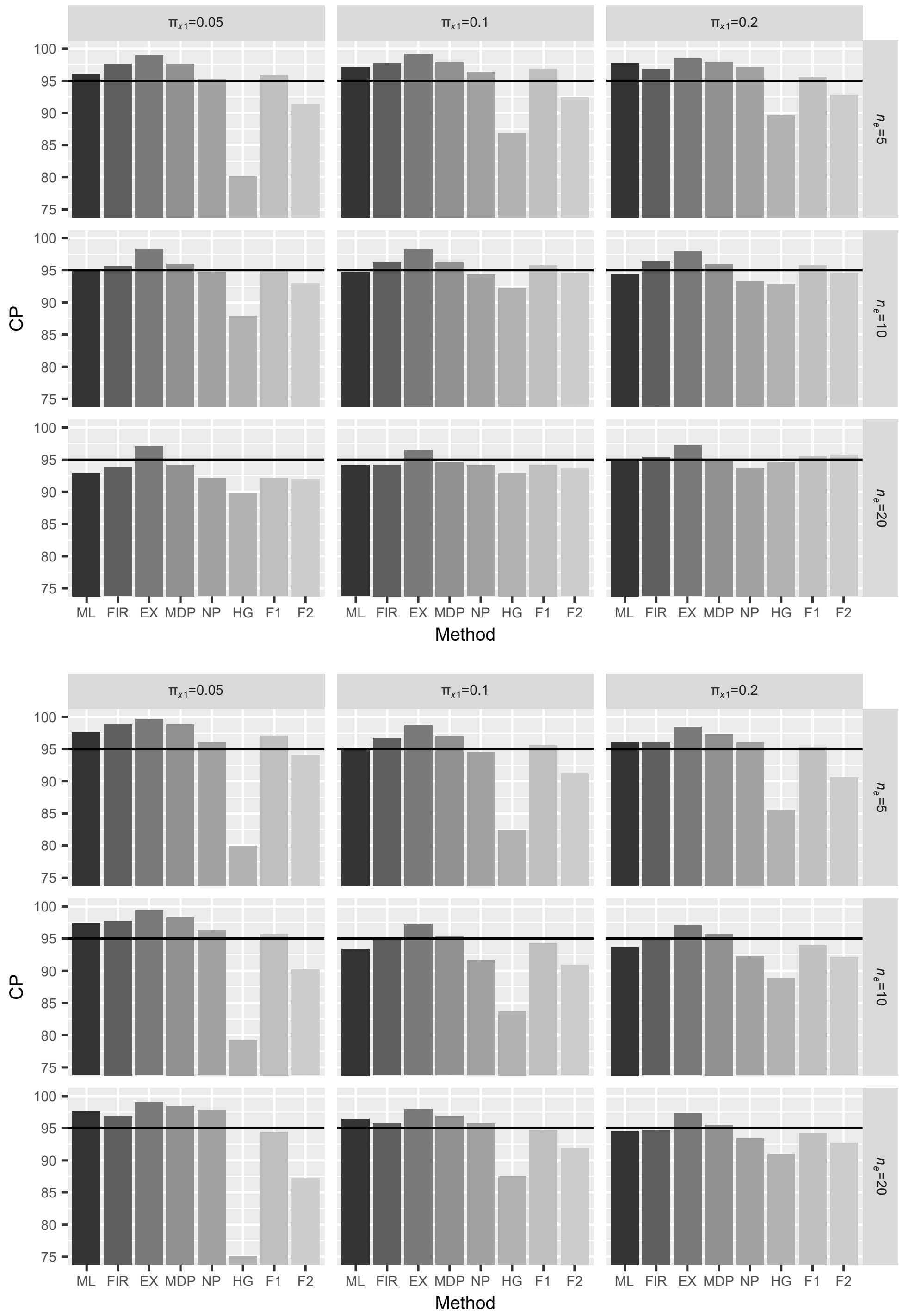

eFigure 33. 95% CI coverage probability under true OR = 16 in scenario 1 (top, n = 100; bottom, n = 1000). EX: exact method; F1: Bayesian data augmentation with log $F(1, 1)$; F2: Bayesian data augmentation with log $F(2, 2)$; FIR: Firth's method; HG: Bayesian method with hyper-g prior; MDP: mid P-type exact method; ML: ML method; NP: Bayesian method with $N(0, 100)$ prior.

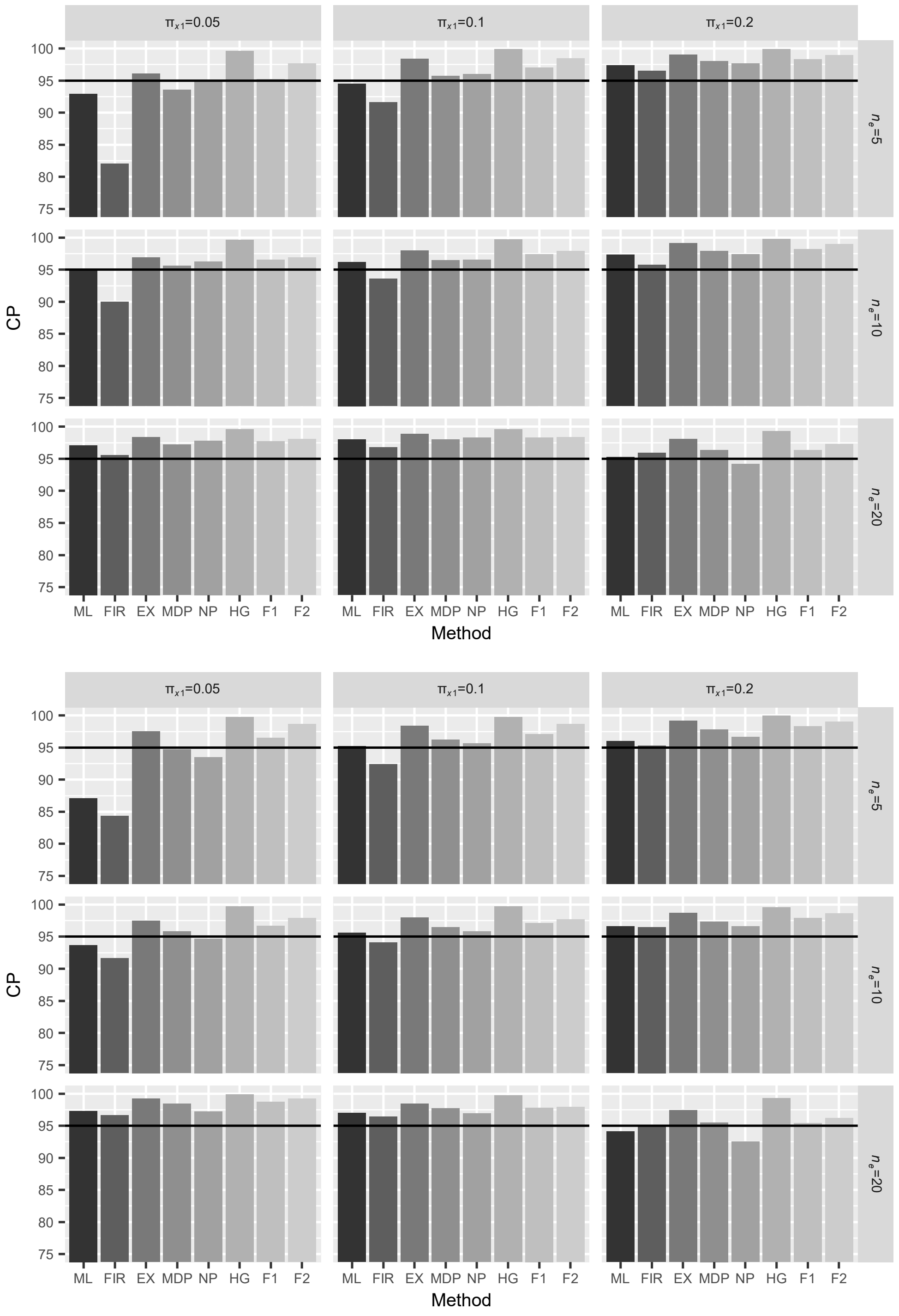

eFigure 34. 95% CI coverage probability under true OR = 1 in scenario 2 (top, n = 100; bottom, n = 1000). EX: exact method; F1: Bayesian data augmentation with log $F(1, 1)$; F2: Bayesian data augmentation with log $F(2, 2)$; FIR: Firth's method; HG: Bayesian method with hyper-g prior; MDP: mid P-type exact method; ML: ML method; NP: Bayesian method with $N(0, 100)$ prior.

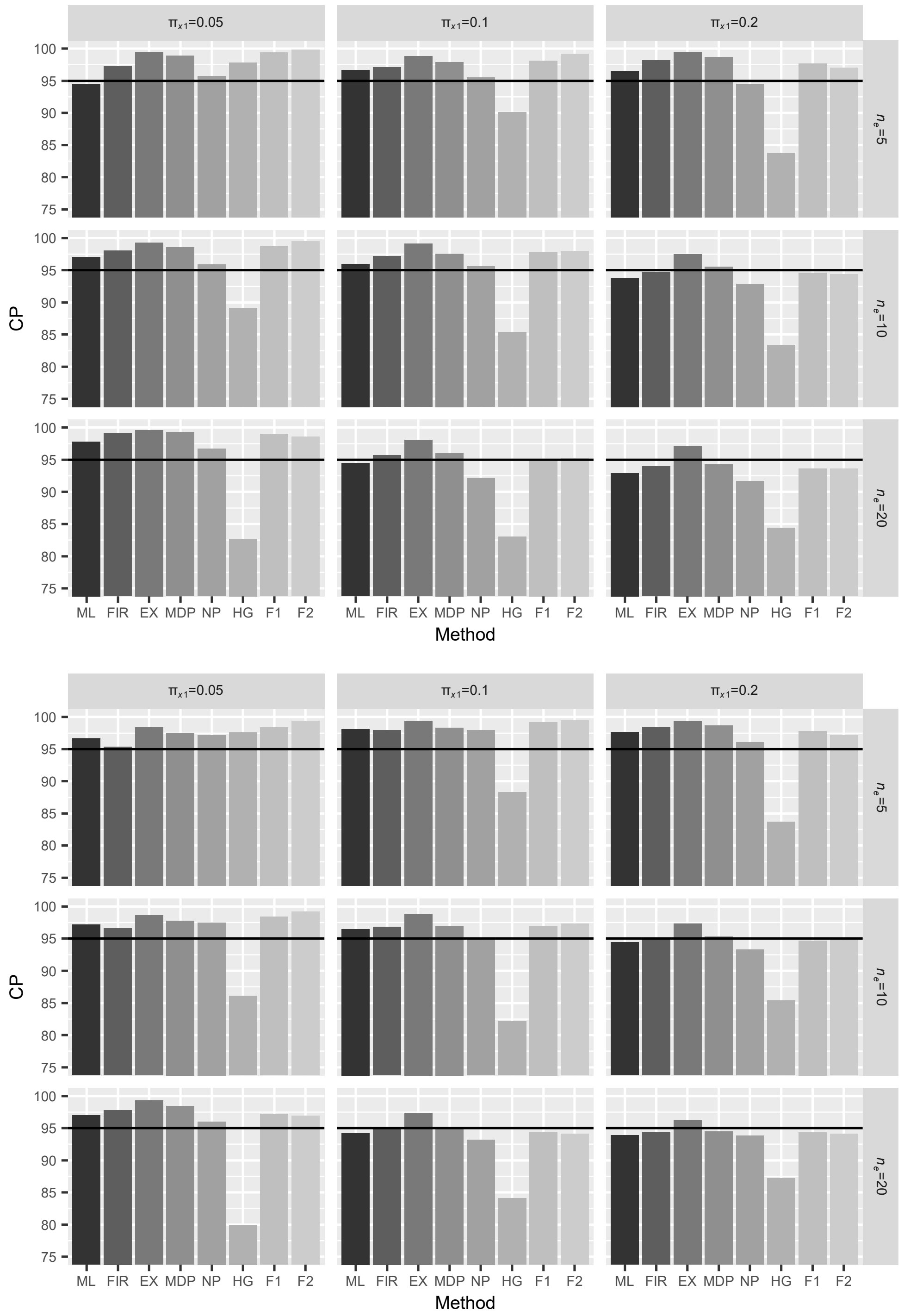

eFigure 35. 95% CI coverage probability under true OR = 4 in scenario 2 (top, n = 100; bottom, n = 1000). EX: exact method; F1: Bayesian data augmentation with log $F(1, 1)$; F2: Bayesian data augmentation with log $F(2, 2)$; FIR: Firth's method; HG: Bayesian method with hyper-g prior; MDP: mid P-type exact method; ML: ML method; NP: Bayesian method with $N(0, 100)$ prior.

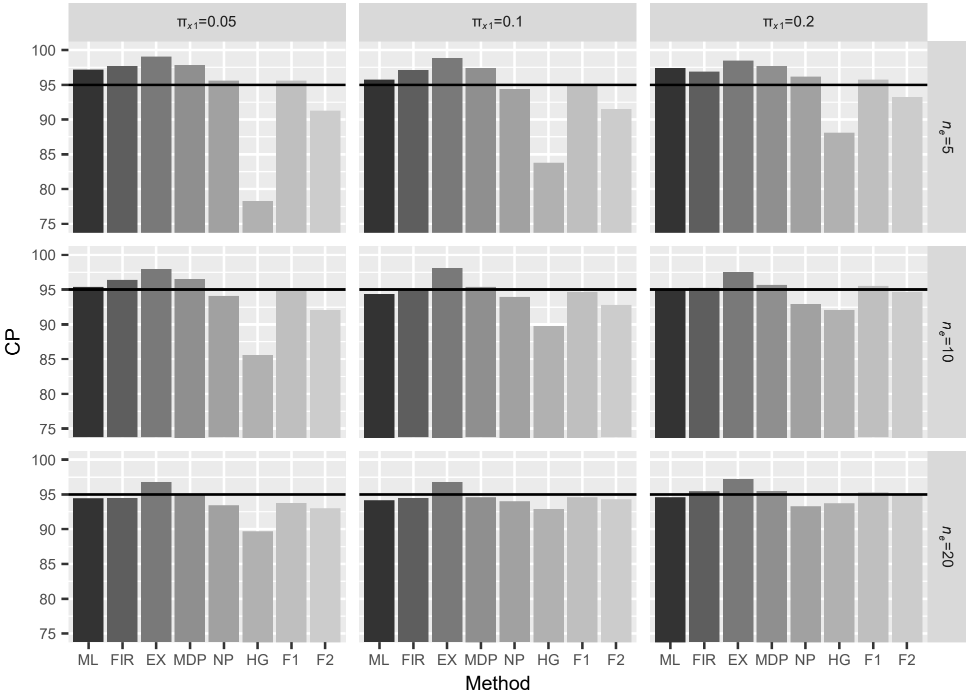

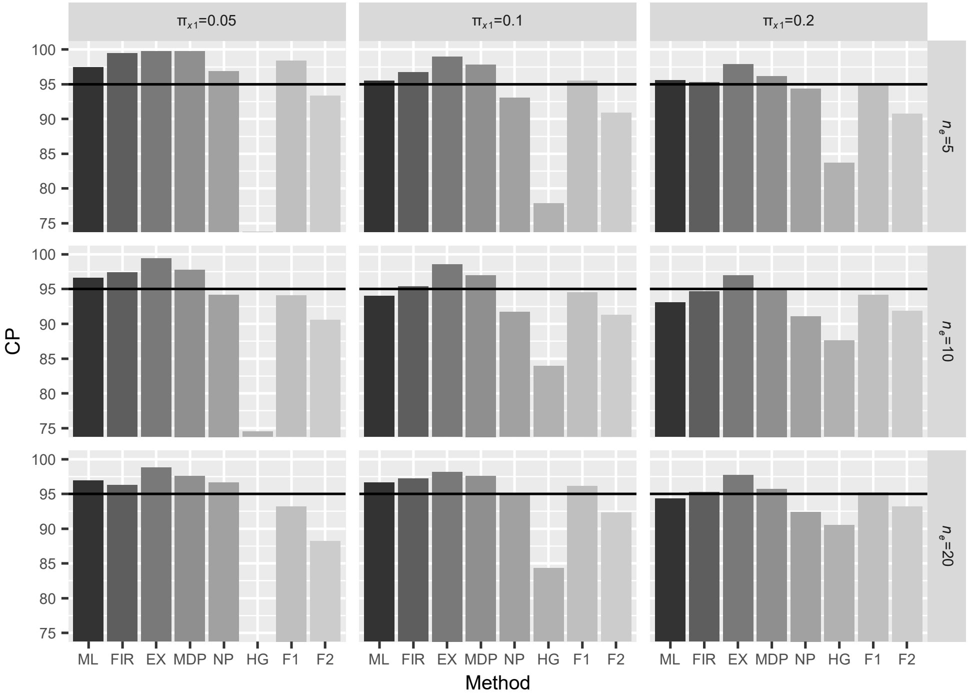

eFigure 36. 95% CI coverage probability under true OR = 16 in scenario 2 (top, n = 100; bottom, n = 1000). EX: exact method; F1: Bayesian data augmentation with log $F(1, 1)$; F2: Bayesian data augmentation with log $F(2, 2)$; FIR: Firth's method; HG: Bayesian method with hyper-g prior; MDP: mid P-type exact method; ML: ML method; NP: Bayesian method with $N(0, 100)$ prior.

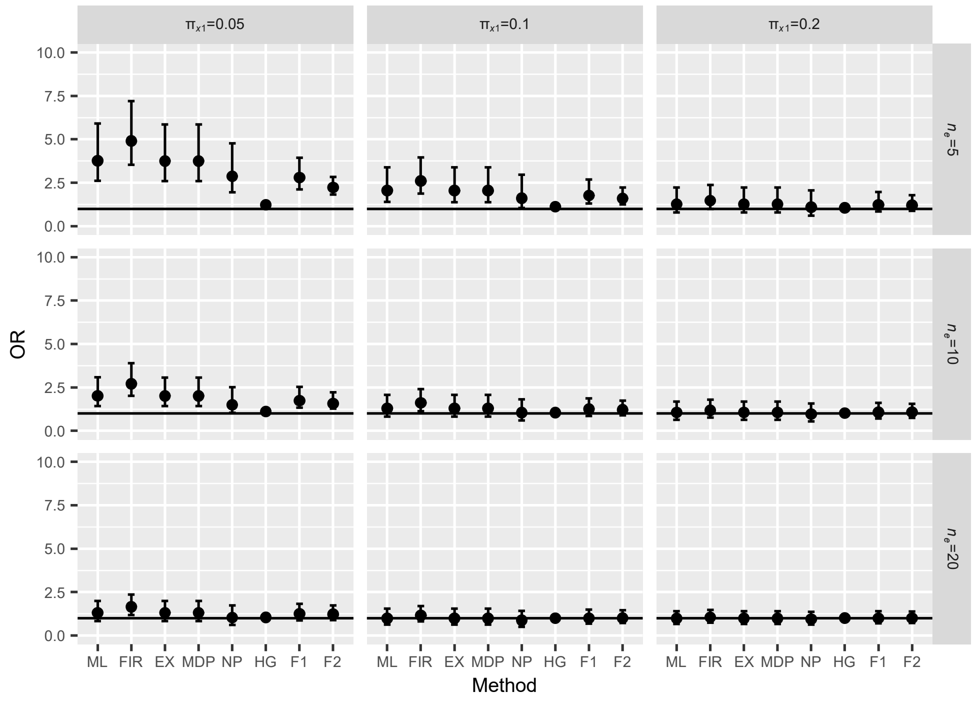

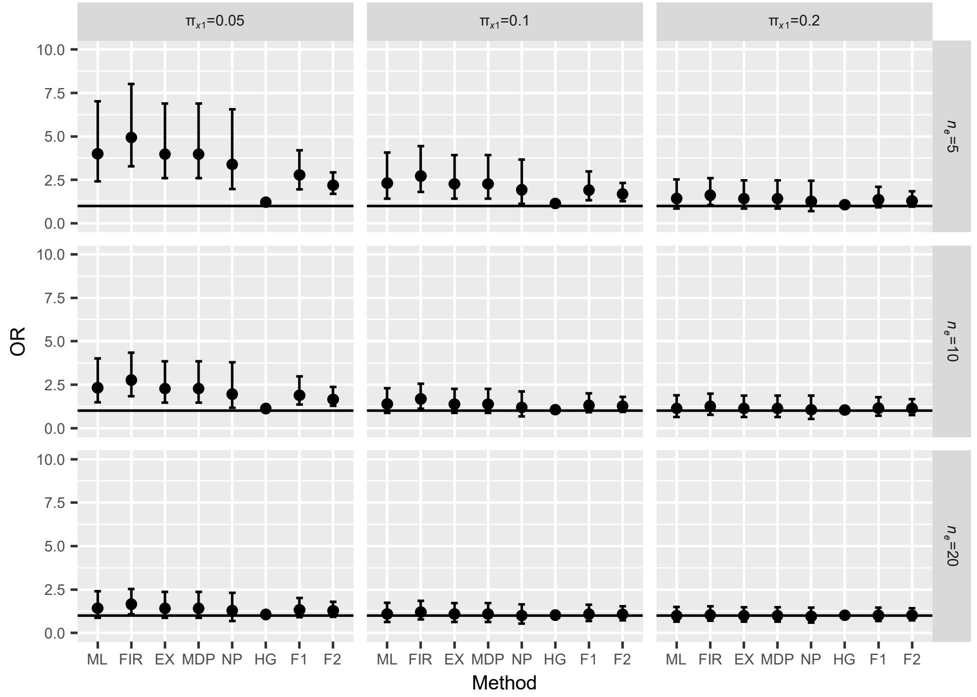

eFigure 37. 95% CI coverage probability under true OR = 1 in scenario 3 (top, n = 100; bottom, n = 1000). EX: exact method; F1: Bayesian data augmentation with log $F(1, 1)$; F2: Bayesian data augmentation with log $F(2, 2)$; FIR: Firth's method; HG: Bayesian method with hyper-g prior; MDP: mid P-type exact method; ML: ML method; NP: Bayesian method with $N(0, 100)$ prior.

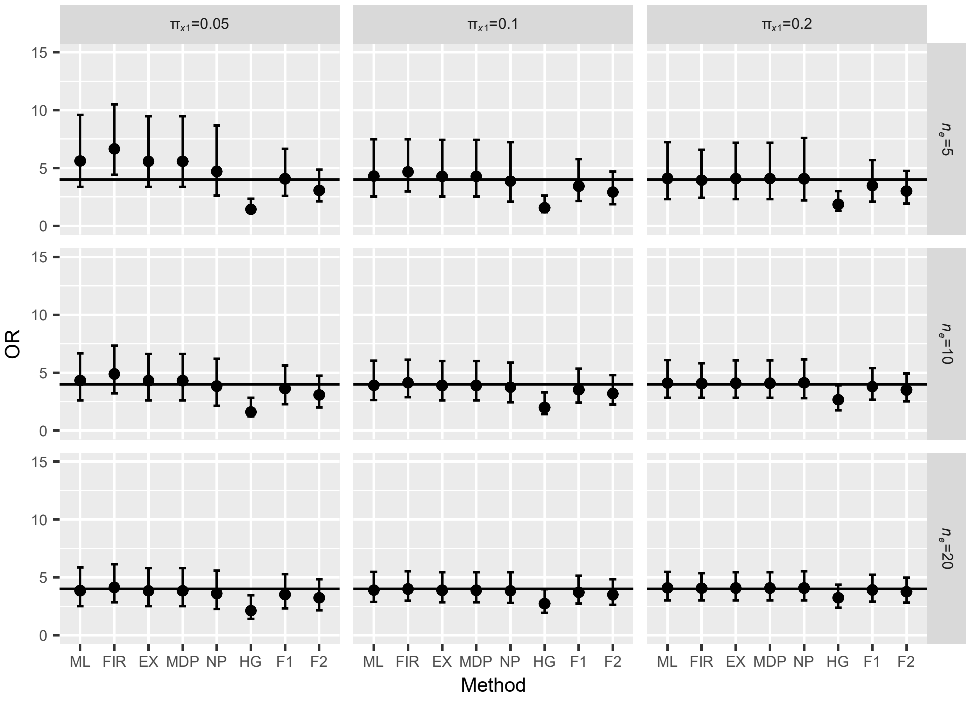

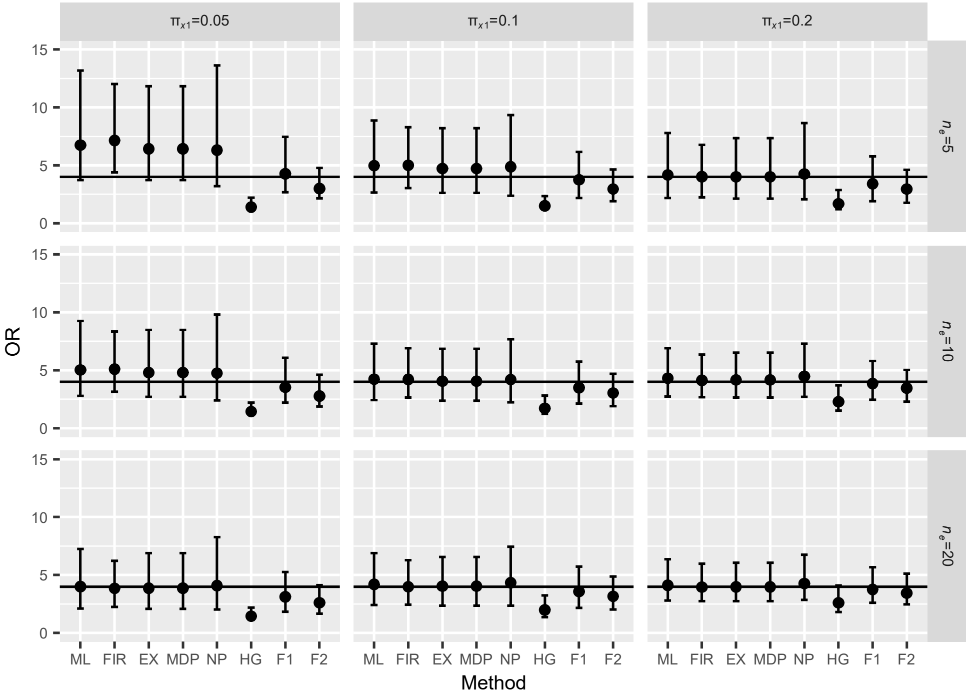

eFigure 38. 95% CI coverage probability under true OR = 4 in scenario 3 (top, n = 100; bottom, n = 1000). EX: exact method; F1: Bayesian data augmentation with log F(1, 1); F2: Bayesian data augmentation with log F(2, 2); FIR: Firth's method; HG: Bayesian method with hyper-g prior; MDP: mid P-type exact method; ML: ML method; NP: Bayesian method with N(0, 100) prior.

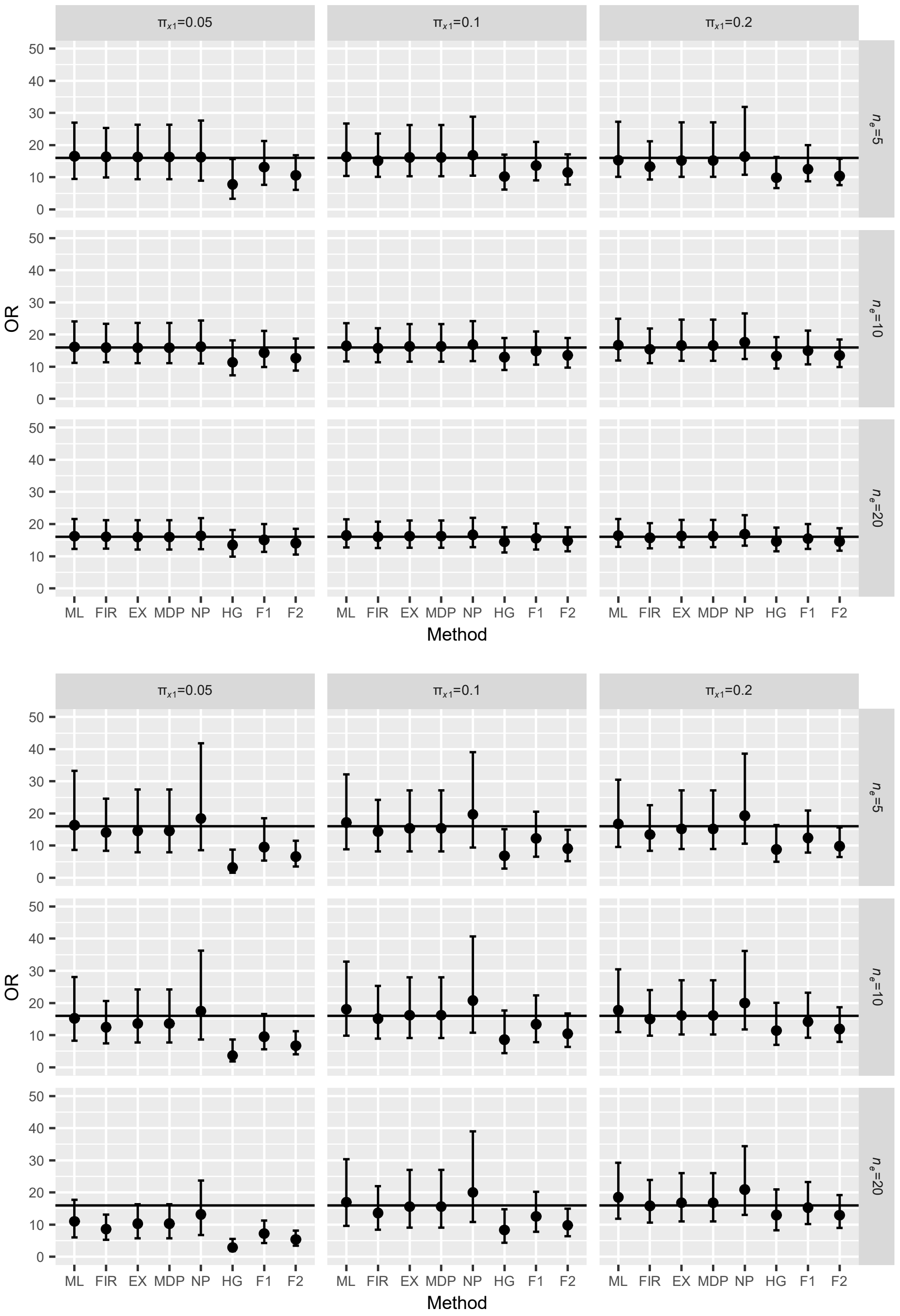

eFigure 39. 95% CI coverage probability under true OR = 16 in scenario 3 (top, n = 100; bottom, n = 1000). EX: exact method; F1: Bayesian data augmentation with log $F(1, 1)$; F2: Bayesian data augmentation with log $F(2, 2)$; FIR: Firth's method; HG: Bayesian method with hyper-g prior; MDP: mid P-type exact method; ML: ML method; NP: Bayesian method with $N(0, 100)$ prior.

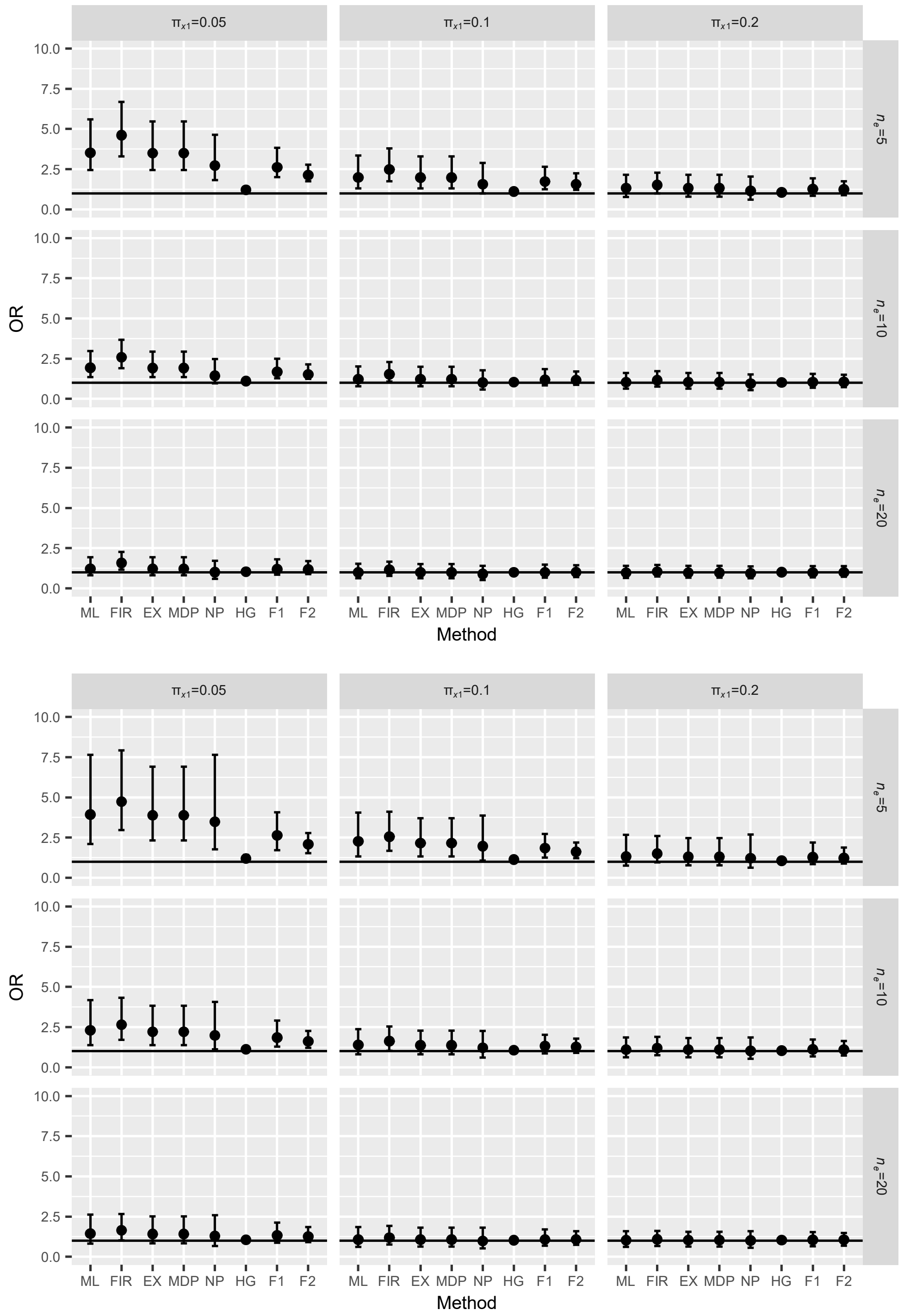

eFigure 40. 95% CI coverage probability under true OR = 1 in scenario 4 (top, n = 100; bottom, n = 1000). EX: exact method; F1: Bayesian data augmentation with log F(1, 1); F2: Bayesian data augmentation with log F(2, 2); FIR: Firth's method; HG: Bayesian method with hyper-g prior; MDP: mid P-type exact method; ML: ML method; NP: Bayesian method with N(0, 100) prior.

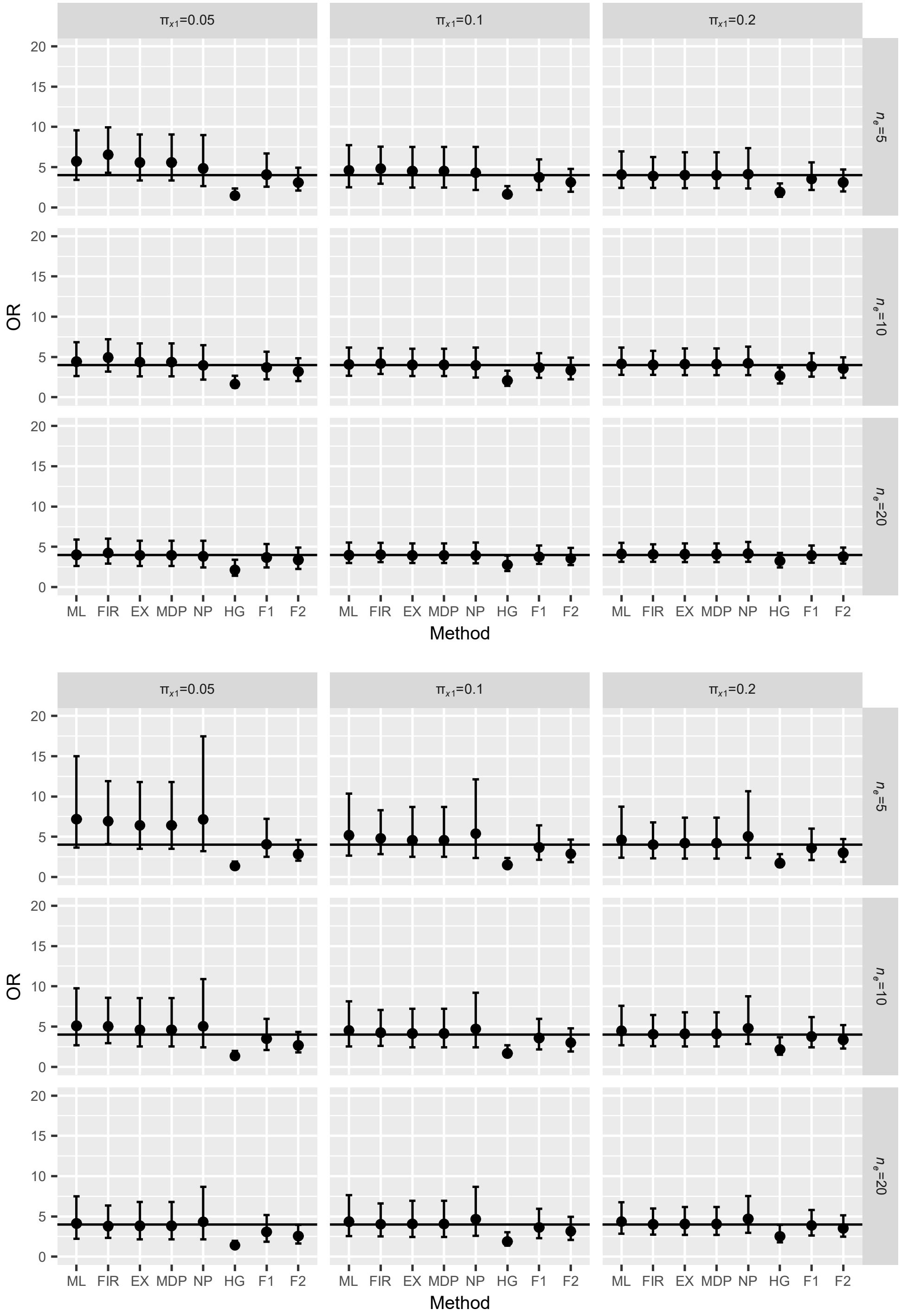

eFigure 41. 95% CI coverage probability under true OR = 4 in scenario 4 (top, n = 100; bottom, n = 1000). EX: exact method; F1: Bayesian data augmentation with log $F(1, 1)$; F2: Bayesian data augmentation with log $F(2, 2)$; FIR: Firth's method; HG: Bayesian method with hyper-g prior; MDP: mid P-type exact method; ML: ML method; NP: Bayesian method with $N(0, 100)$ prior.

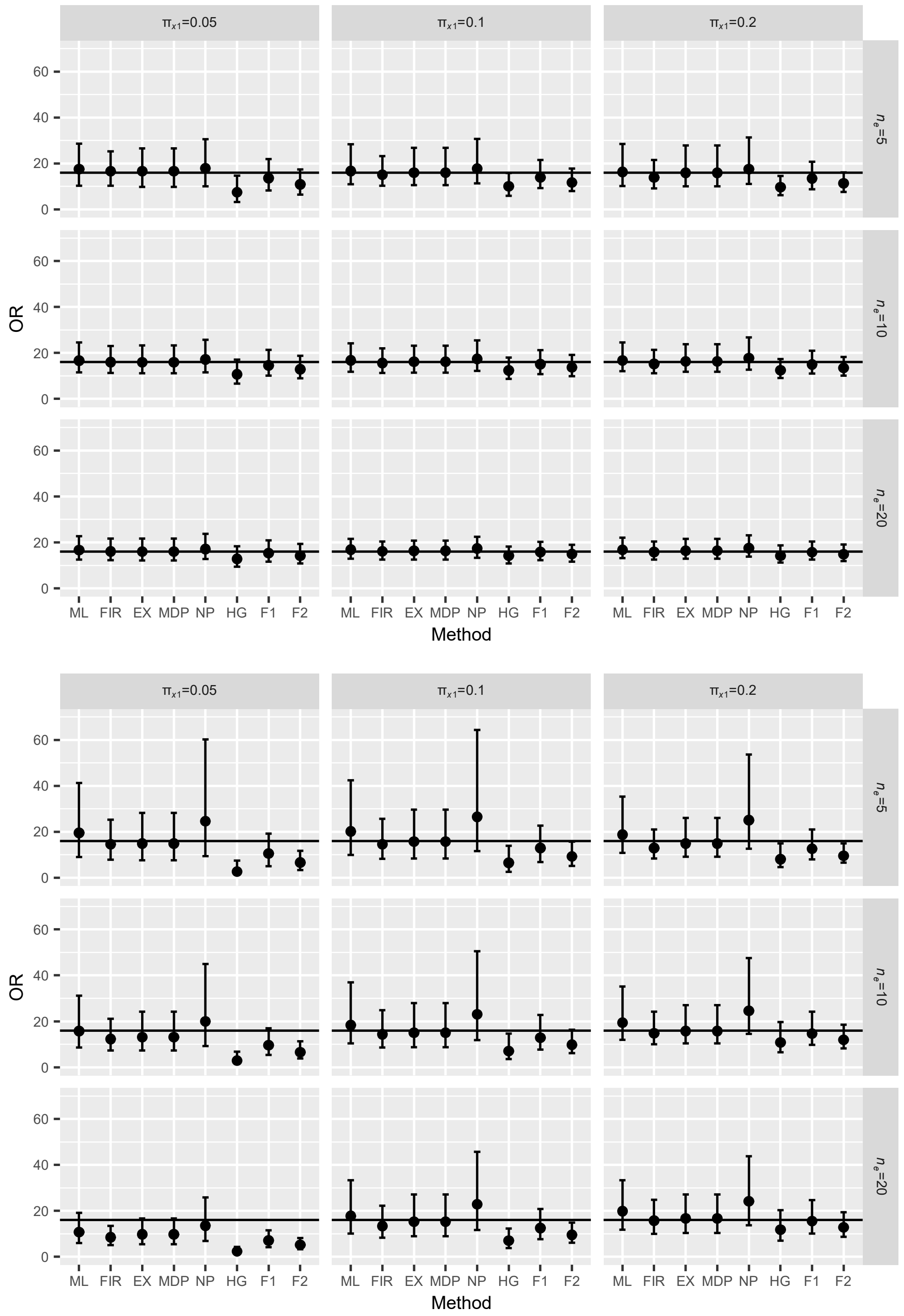

eFigure 42. 95% CI coverage probability under true OR = 16 in scenario 4 (top, n = 100; bottom, n = 1000). EX: exact method; F1: Bayesian data augmentation with log F(1, 1); F2: Bayesian data augmentation with log F(2, 2); FIR: Firth's method; HG: Bayesian method with hyper-g prior; MDP: mid P-type exact method; ML: ML method; NP: Bayesian method with N(0, 100) prior.

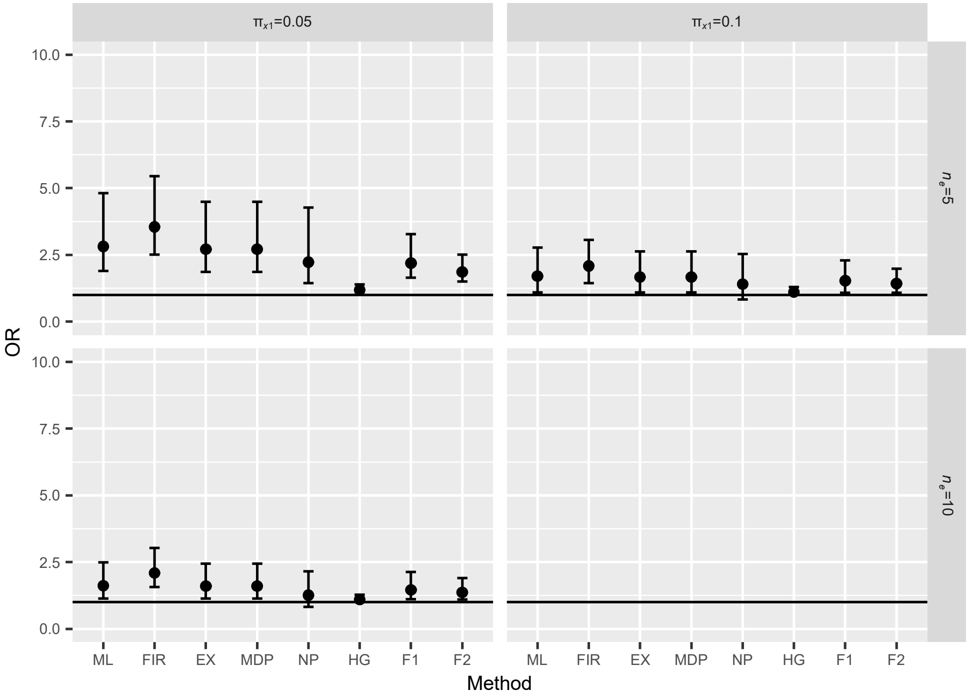

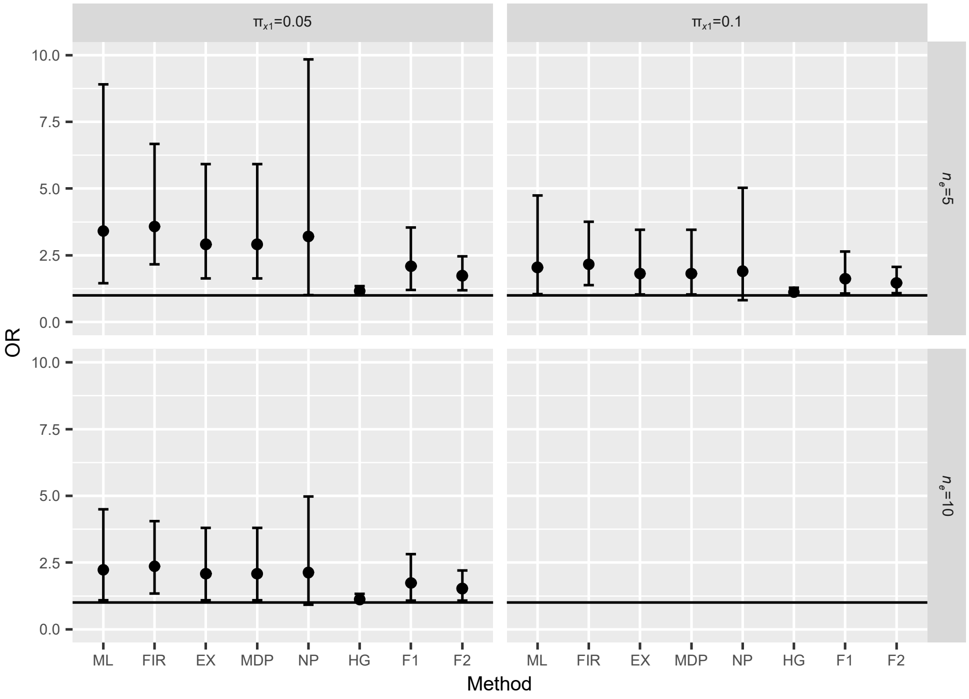

eFigure 43. 95% CI coverage probability under true OR = 1 in scenario 5 (top, n = 100; bottom, n = 1000). EX: exact method; F1: Bayesian data augmentation with log F(1, 1); F2: Bayesian data augmentation with log F(2, 2); FIR: Firth's method; HG: Bayesian method with hyper-g prior; MDP: mid P-type exact method; ML: ML method; NP: Bayesian method with N(0, 100) prior.

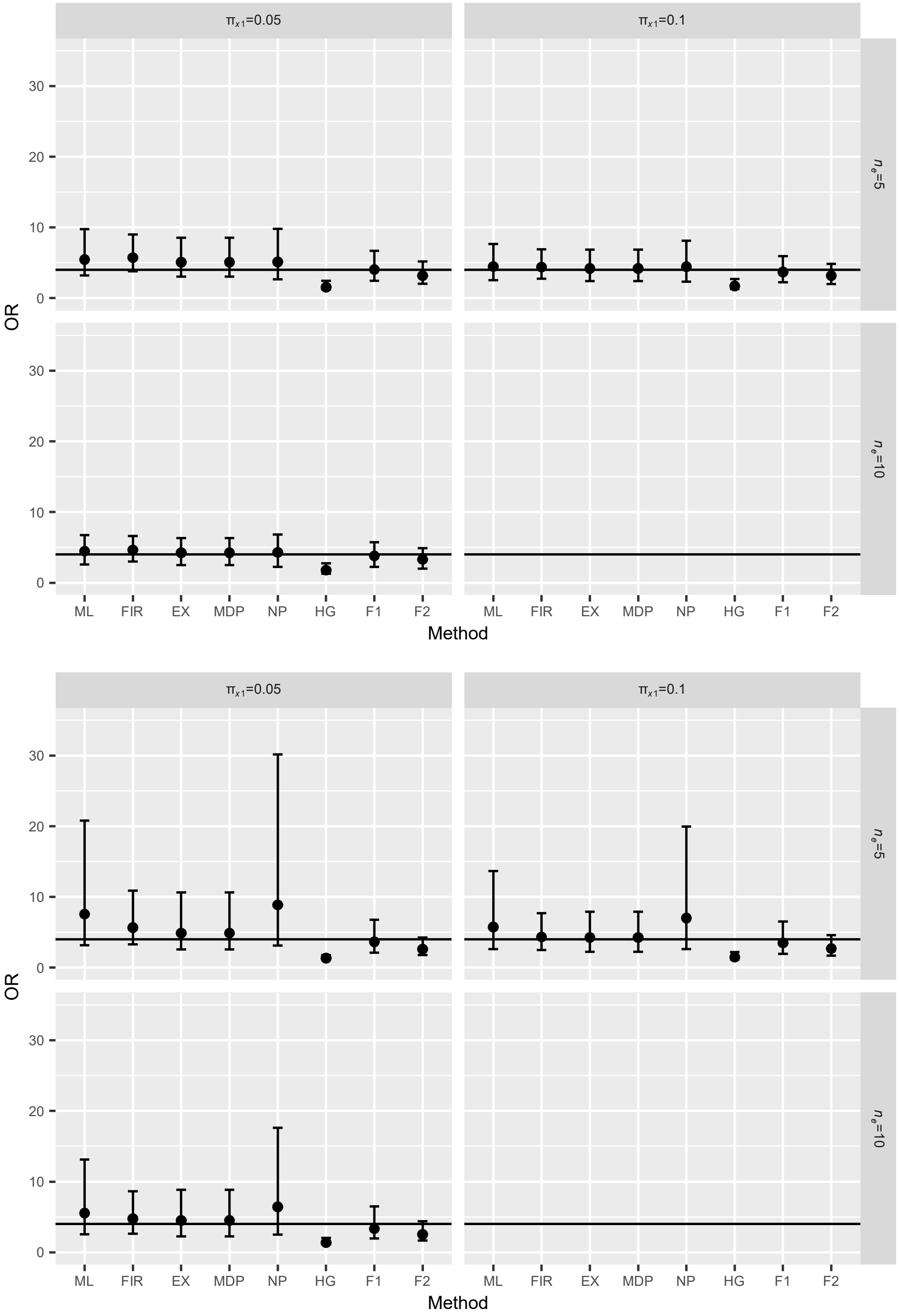

eFigure 44. 95% CI coverage probability under true OR = 4 in scenario 5 (top, n = 100; bottom, n = 1000). EX: exact method; F1: Bayesian data augmentation with log $F(1, 1)$; F2: Bayesian data augmentation with log $F(2, 2)$; FIR: Firth's method; HG: Bayesian method with hyper-g prior; MDP: mid P-type exact method; ML: ML method; NP: Bayesian method with $N(0, 100)$ prior.

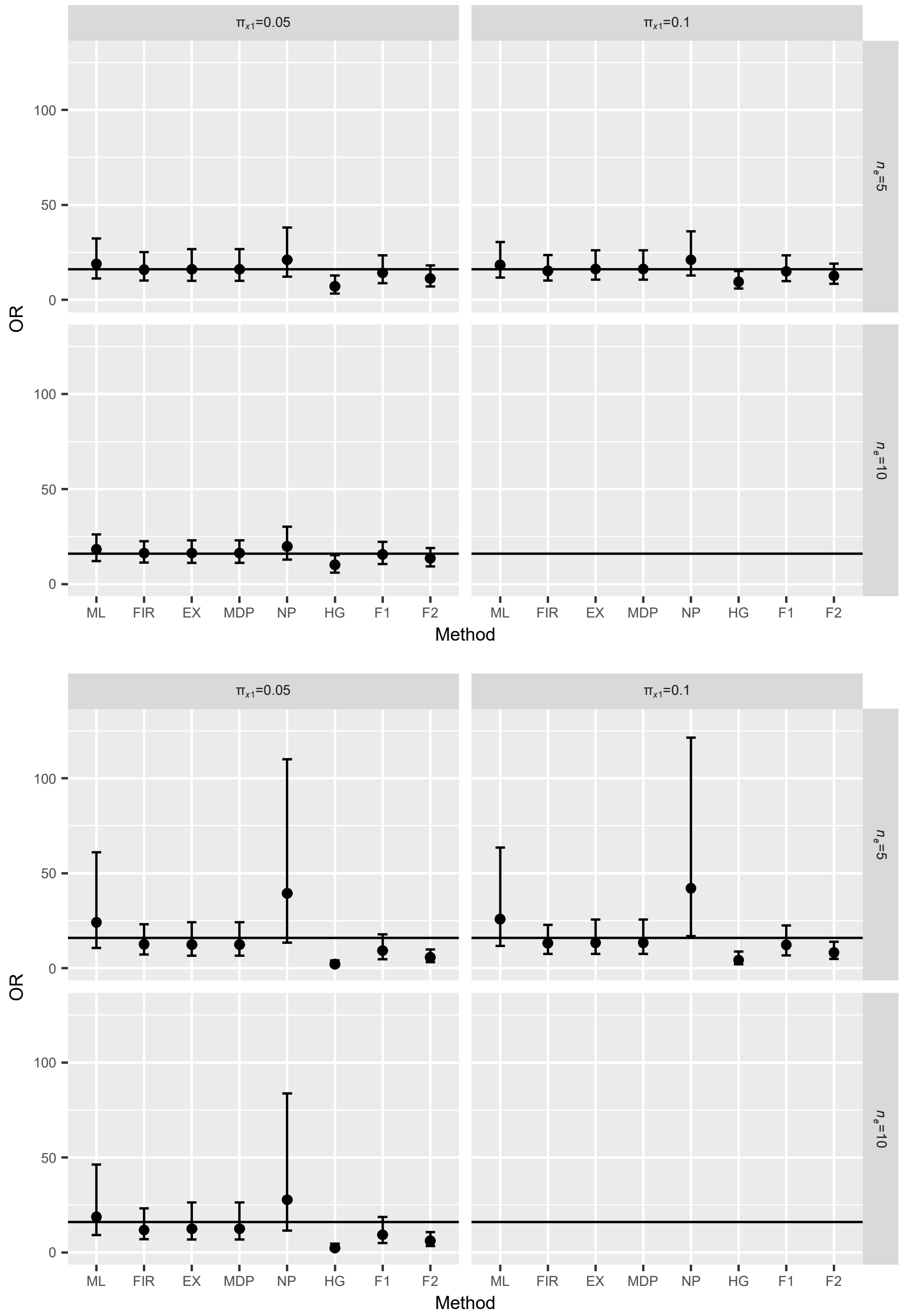

eFigure 45. 95% CI coverage probability under true OR = 16 in scenario 5 (top, n = 100; bottom, n = 1000). EX: exact method; F1: Bayesian data augmentation with log F(1, 1); F2: Bayesian data augmentation with log F(2, 2); FIR: Firth's method; HG: Bayesian method with hyper-g prior; MDP: mid P-type exact method; ML: ML method; NP: Bayesian method with N(0, 100) prior.

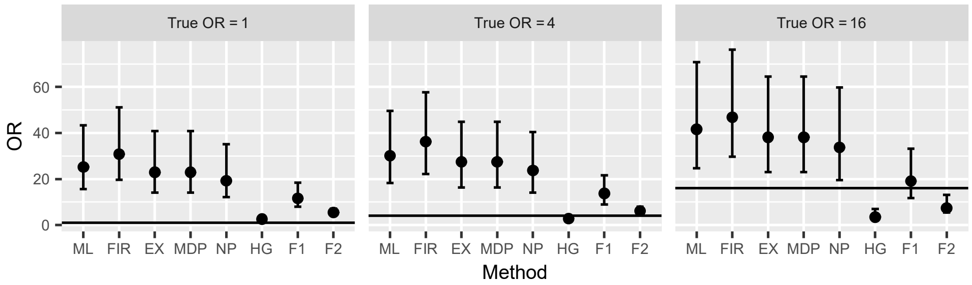

eFigure 46. Distribution of simulated OR in scenario 6. The square represents the median and the error bar represents quartiles 1 and 3. The solid horizontal line is the true OR value. EX: exact method; F1: Bayesian data augmentation with log $F(1, 1)$; F2: Bayesian data augmentation with log $F(2, 2)$; FIR: Firth's method; HG: Bayesian method with hyper-g prior; MDP: mid P-type exact method; ML: ML method; NP: Bayesian method with $N(0, 100)$ prior.

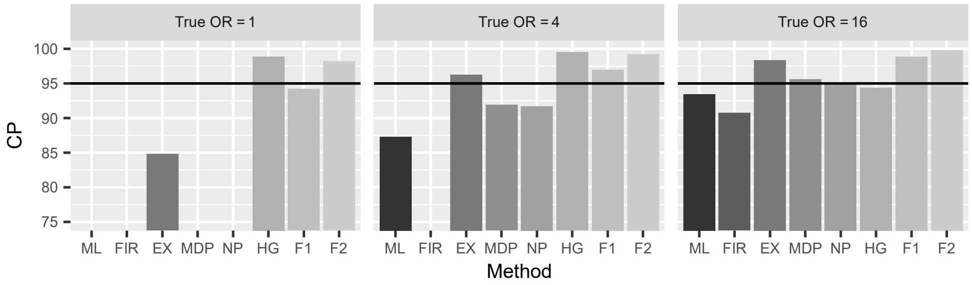

eFigure 47. 95% CI coverage probability (CP) in scenario 6. When true OR = 1, the CPs for ML, FIR, MDP, and NP are 43.3, 13.3, 59.5, and 67.4%, respectively. The CP for FIR is 61.0% when true OR = 4. EX: exact method; F1: Bayesian data augmentation with log $F(1, 1)$; F2: Bayesian data augmentation with log $F(2, 2)$; FIR: Firth's method; HG: Bayesian method with hyper-g prior; MDP: mid P-type exact method; ML: ML method; NP: Bayesian method with $N(0, 100)$ prior.

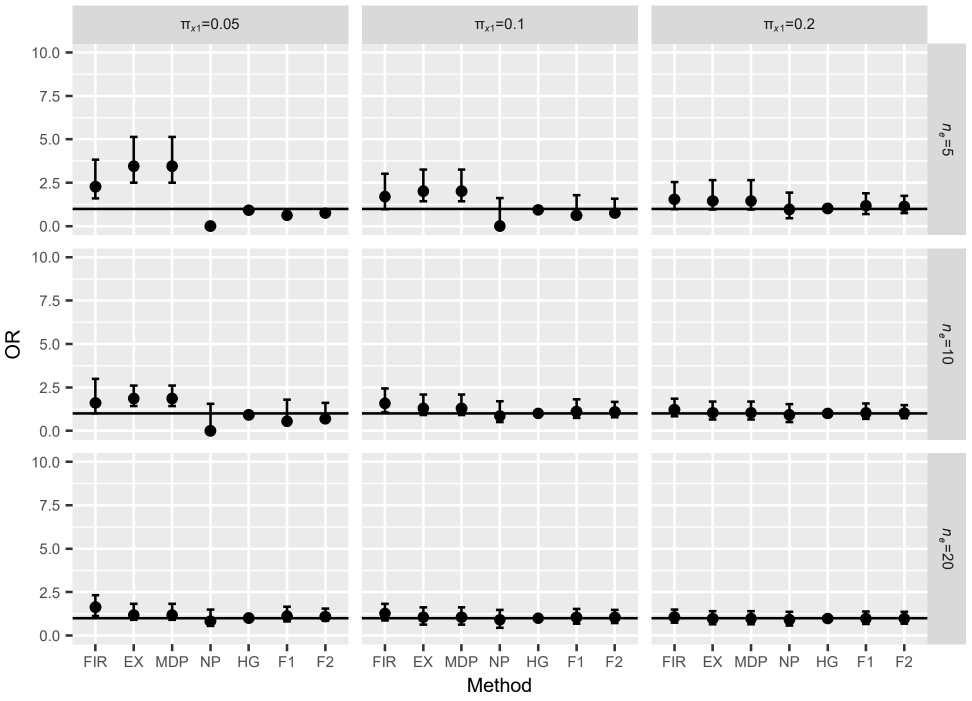

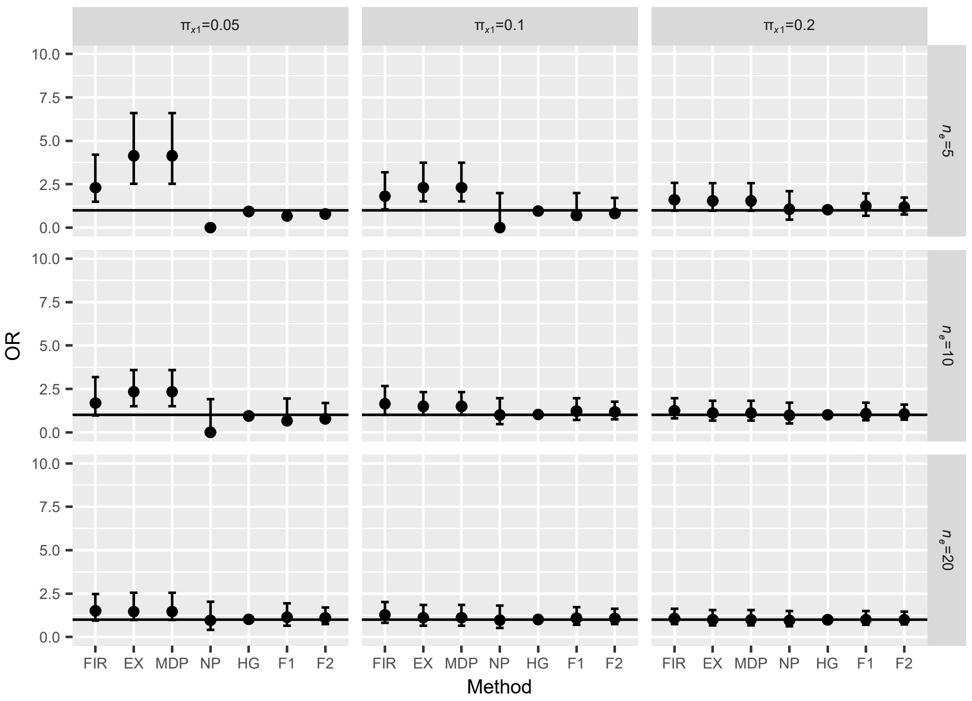

eFigure 48: Distribution of simulated OR under true OR = 1 in scenario 1 (top, n = 100; bottom, n = 1000). The square represents the median and the error bar represents quartiles 1 and 3. The solid horizontal line is the true OR value. EX: exact method; F1: Bayesian data augmentation with log $F(1, 1)$; F2: Bayesian data augmentation with log $F(2, 2)$; FIR: Firth's method; HG: Bayesian method with hyper-g prior; MDP: mid P-type exact method; NP: Bayesian method with $N(0, 100)$ prior.

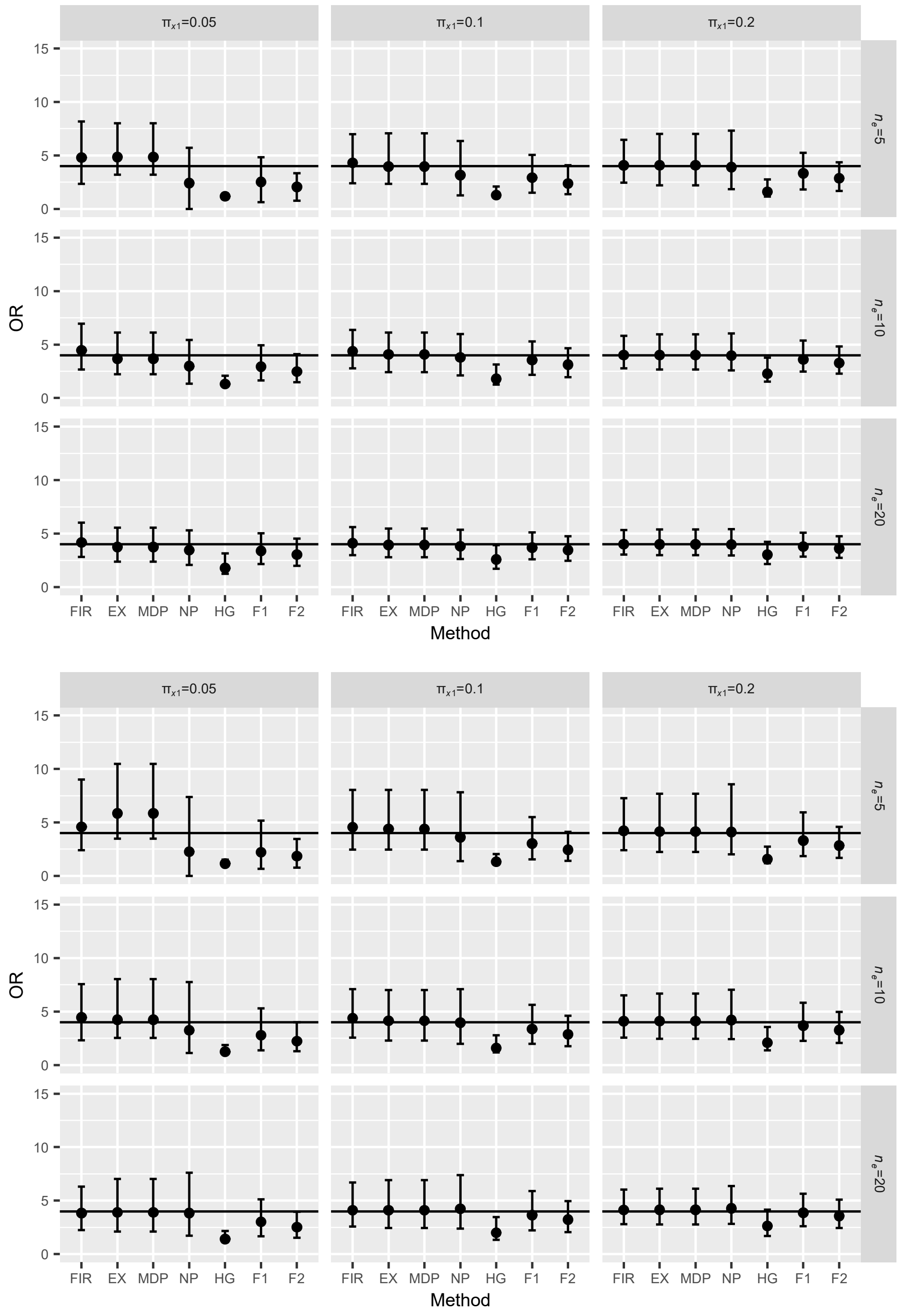

eFigure 49: Distribution of simulated OR under true OR = 4 in scenario 1 (top, n = 100; bottom, n = 1000). The square represents the median and the error bar represents quartiles 1 and 3. The solid horizontal line is the true OR value. EX: exact method; F1: Bayesian data augmentation with log $F(1, 1)$; F2: Bayesian data augmentation with log $F(2, 2)$; FIR: Firth's method; HG: Bayesian method with hyper-g prior; MDP: mid P-type exact method; NP: Bayesian method with $N(0, 100)$ prior.

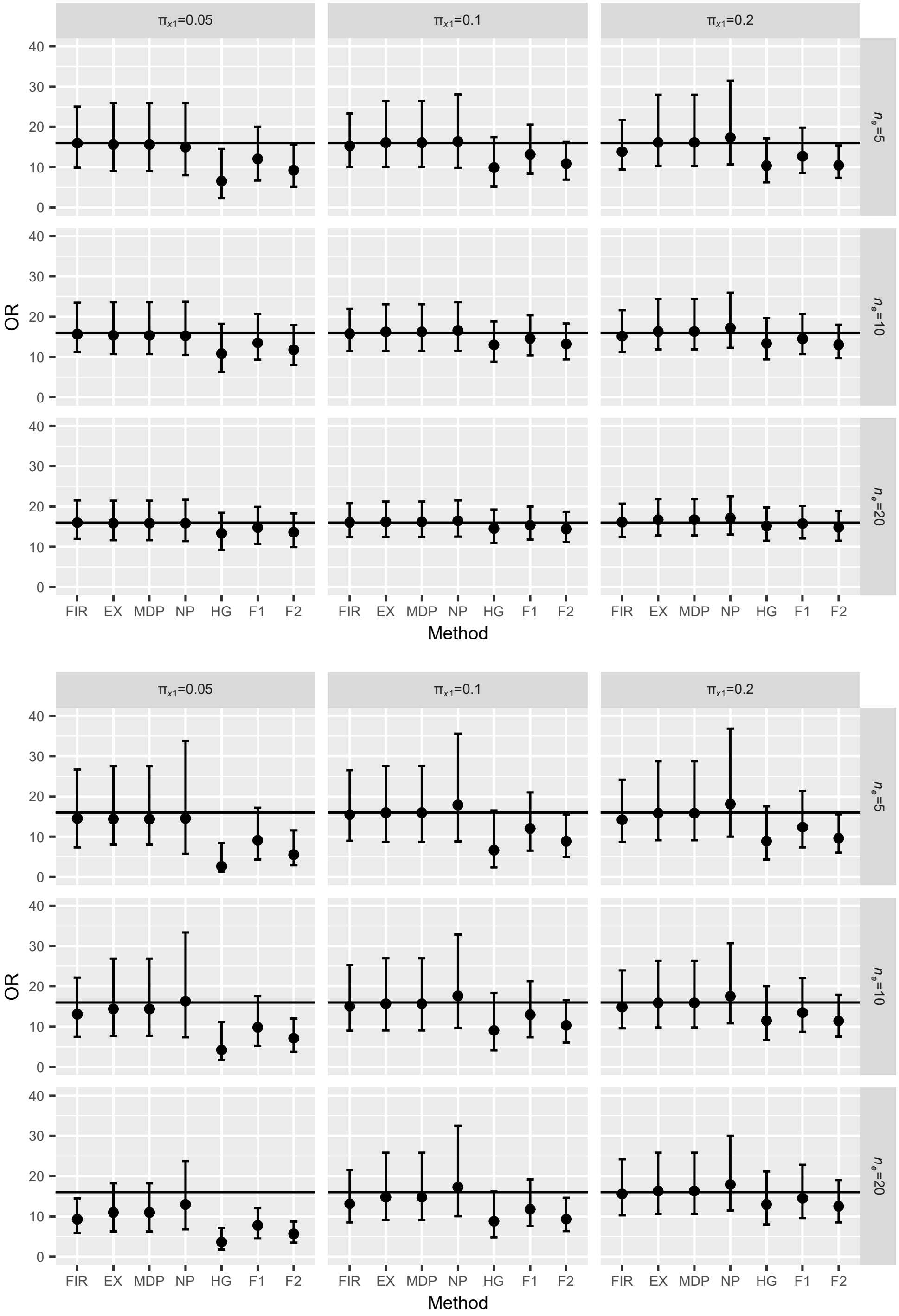

eFigure 50: Distribution of simulated OR under true OR = 16 in scenario 1 (top, n = 100; bottom, n = 1000). The square represents the median and the error bar represents quartiles 1 and 3. The solid horizontal line is the true OR value. EX: exact method; F1: Bayesian data augmentation with log $F(1, 1)$; F2: Bayesian data augmentation with log $F(2, 2)$; FIR: Firth's method; HG: Bayesian method with hyper-g prior; MDP: mid P-type exact method; NP: Bayesian method with $N(0, 100)$ prior.

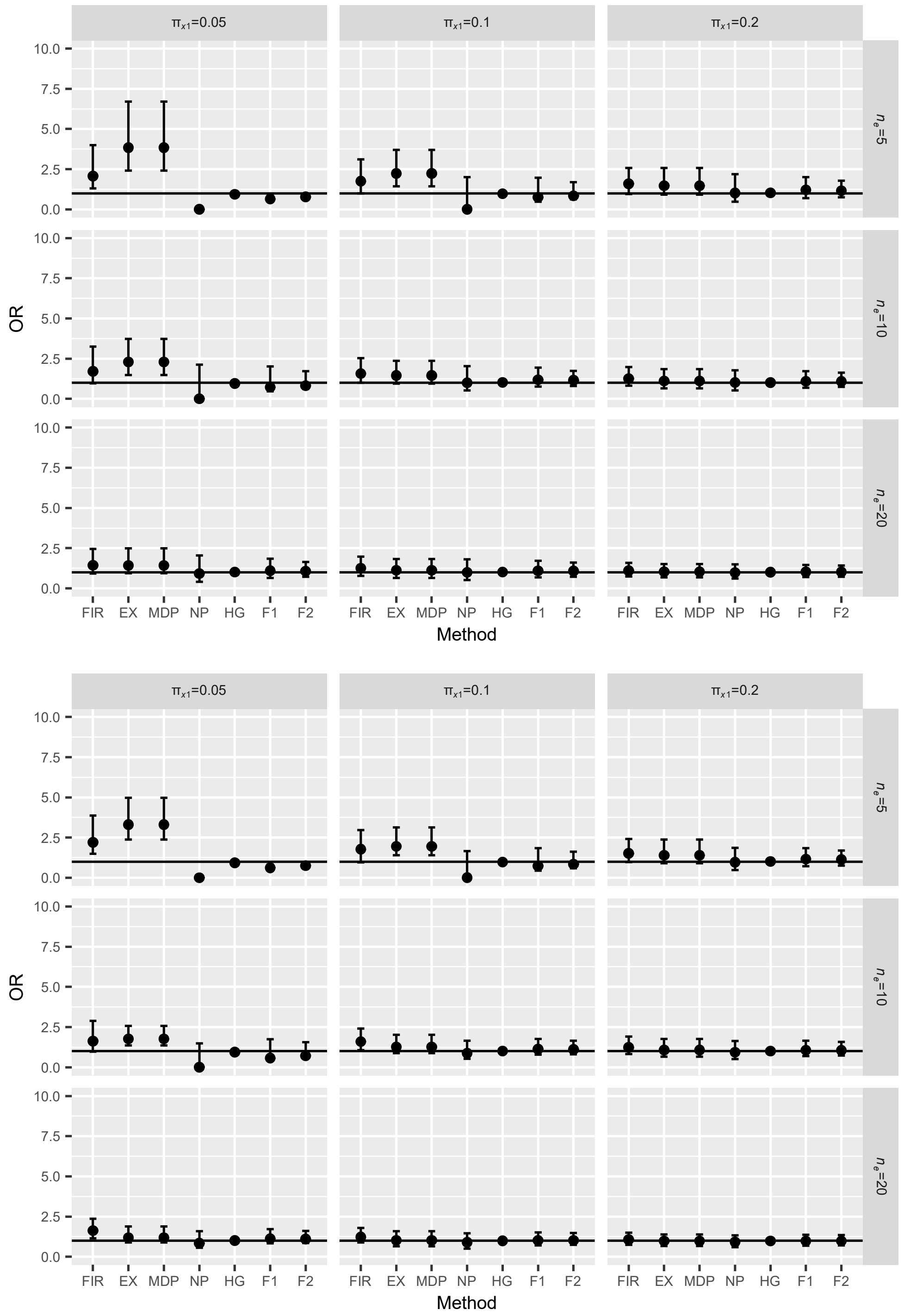

eFigure 51. Distribution of simulated OR under true OR = 1 in scenario 2 (top, n = 100; bottom, n = 1000). The square represents the median and the error bar represents quartiles 1 and 3. The solid horizontal line is the true OR value. EX: exact method; F1: Bayesian data augmentation with log $F(1, 1)$; F2: Bayesian data augmentation with log $F(2, 2)$; FIR: Firth's method; HG: Bayesian method with hyper-g prior; MDP: mid P-type exact method; NP: Bayesian method with $N(0, 100)$ prior.

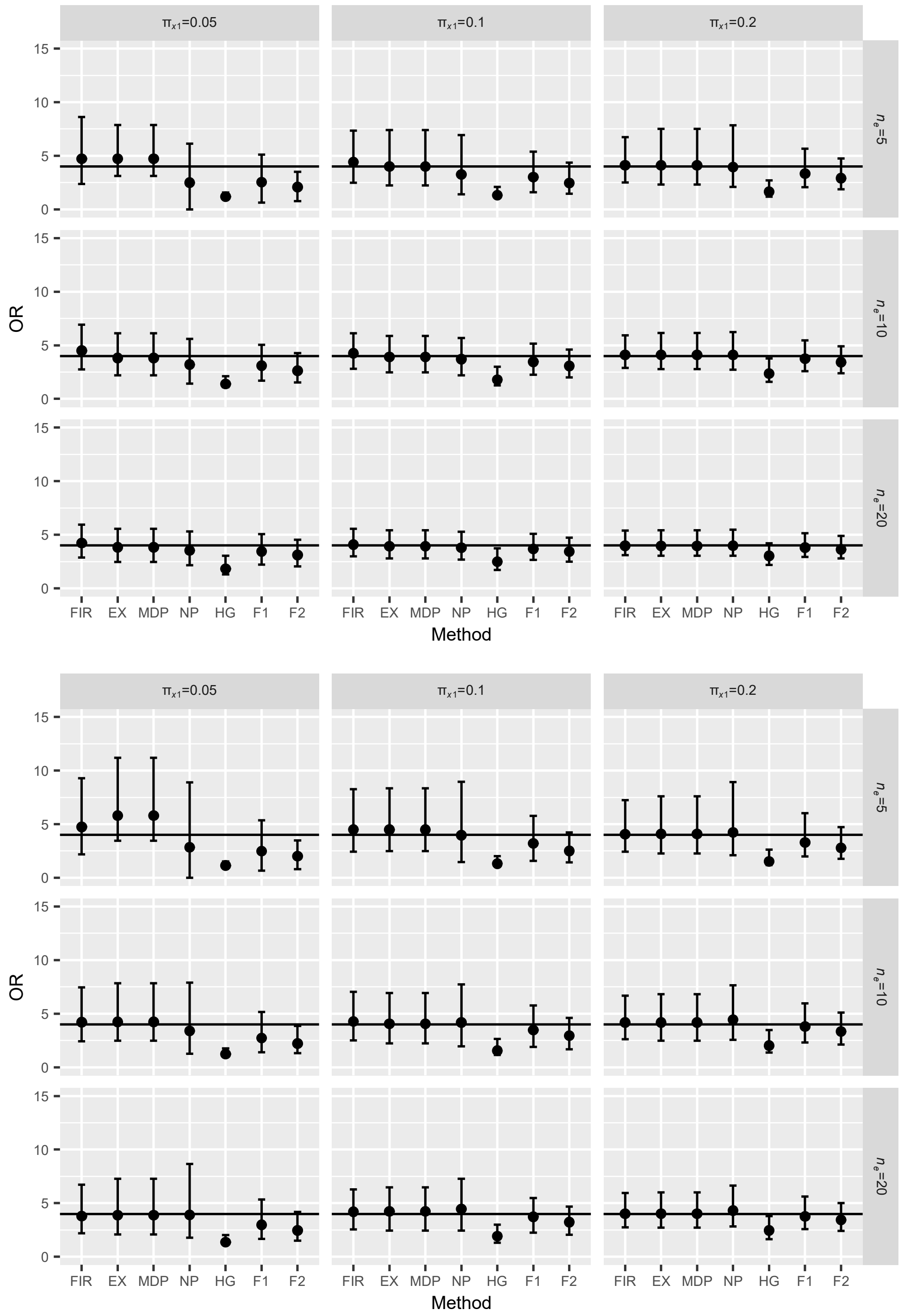

eFigure 52. Distribution of simulated OR under true OR = 4 in scenario 2 (top, n = 100; bottom, n = 1000). The square represents the median and the error bar represents quartiles 1 and 3. The solid horizontal line is the true OR value. EX: exact method; F1: Bayesian data augmentation with log $F(1, 1)$; F2: Bayesian data augmentation with log $F(2, 2)$; FIR: Firth's method; HG: Bayesian method with hyper-g prior; MDP: mid P-type exact method; NP: Bayesian method with $N(0, 100)$ prior.

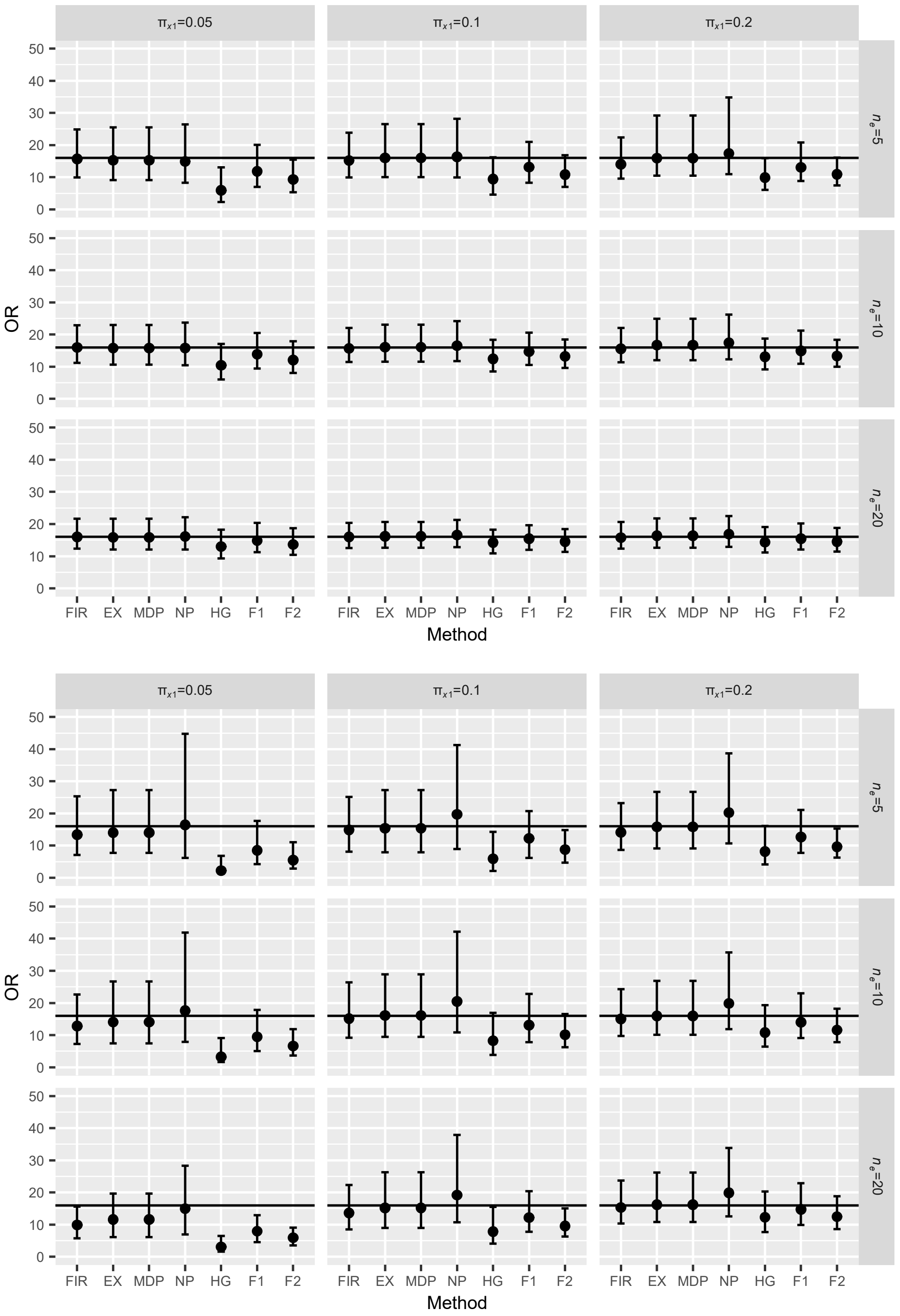

eFigure 53. Distribution of simulated OR under true OR = 16 in scenario 2 (top, n = 100; bottom, n = 1000). The square represents the median and the error bar represents quartiles 1 and 3. The solid horizontal line is the true OR value. EX: exact method; F1: Bayesian data augmentation with log $F(1, 1)$; F2: Bayesian data augmentation with log $F(2, 2)$; FIR: Firth's method; HG: Bayesian method with hyper-g prior; MDP: mid P-type exact method; NP: Bayesian method with $N(0, 100)$ prior.

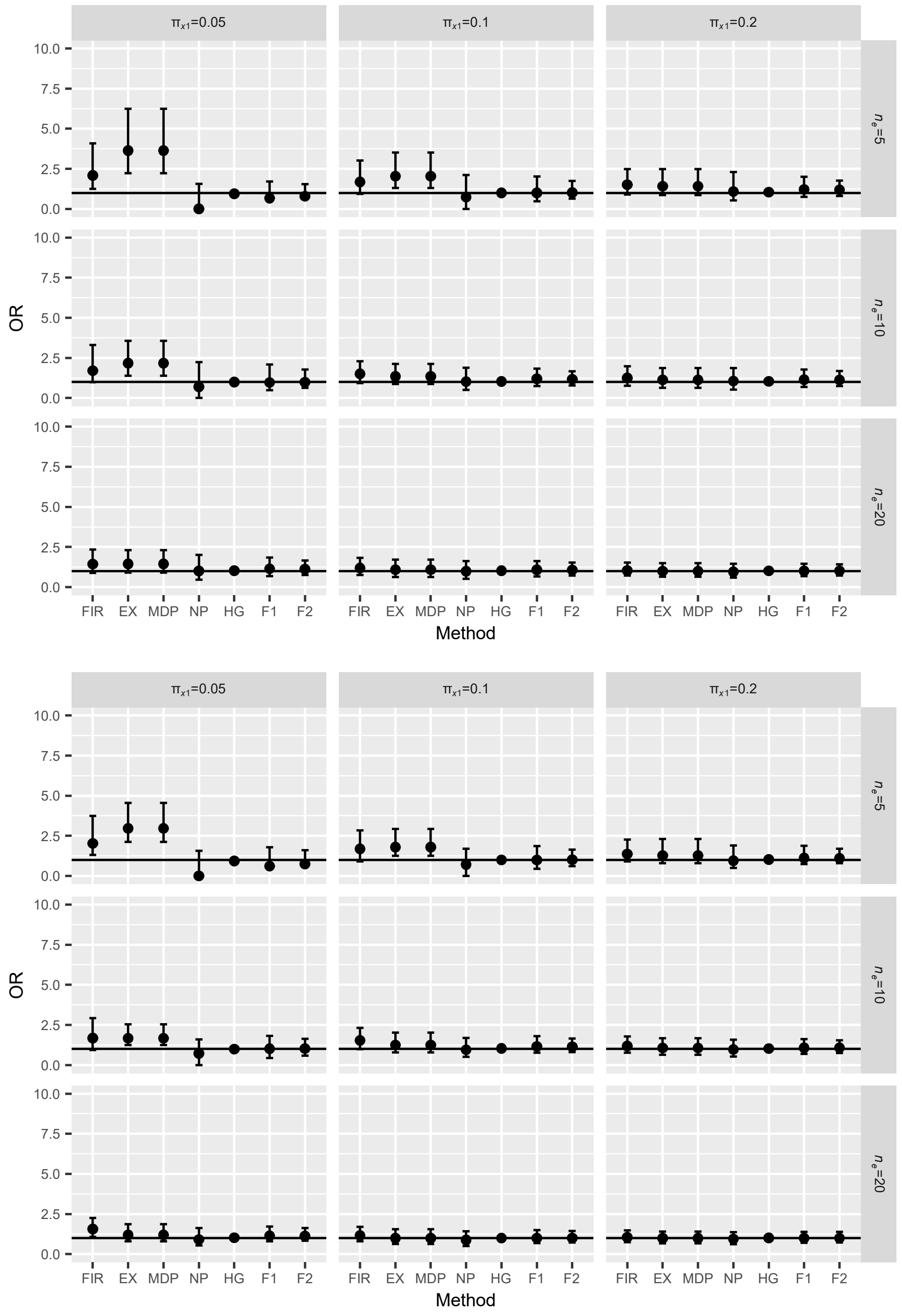

eFigure 54. Distribution of simulated OR under true OR = 1 in scenario 3 (top, n = 100; bottom, n = 1000). The square represents the median and the error bar represents quartiles 1 and 3. The solid horizontal line is the true OR value. EX: exact method; F1: Bayesian data augmentation with log $F(1, 1)$; F2: Bayesian data augmentation with log $F(2, 2)$; FIR: Firth's method; HG: Bayesian method with hyper-g prior; MDP: mid P-type exact method; NP: Bayesian method with $N(0, 100)$ prior.

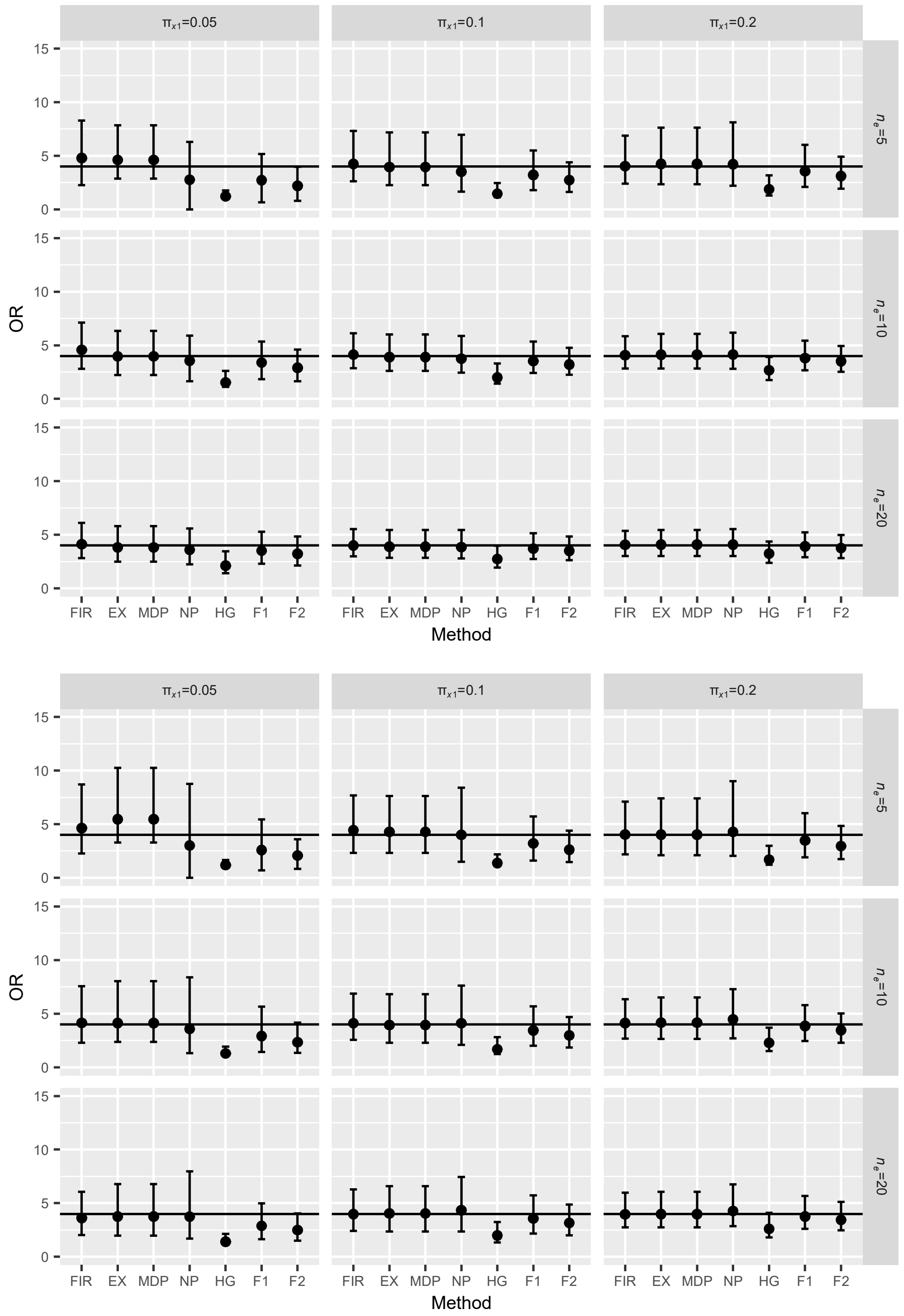

eFigure 55. Distribution of simulated OR under true OR = 4 in scenario 3 (top, n = 100; bottom, n = 1000). The square represents the median and the error bar represents quartiles 1 and 3. The solid horizontal line is the true OR value. EX: exact method; F1: Bayesian data augmentation with log $F(1, 1)$; F2: Bayesian data augmentation with log $F(2, 2)$; FIR: Firth's method; HG: Bayesian method with hyper-g prior; MDP: mid P-type exact method; NP: Bayesian method with $N(0, 100)$ prior.

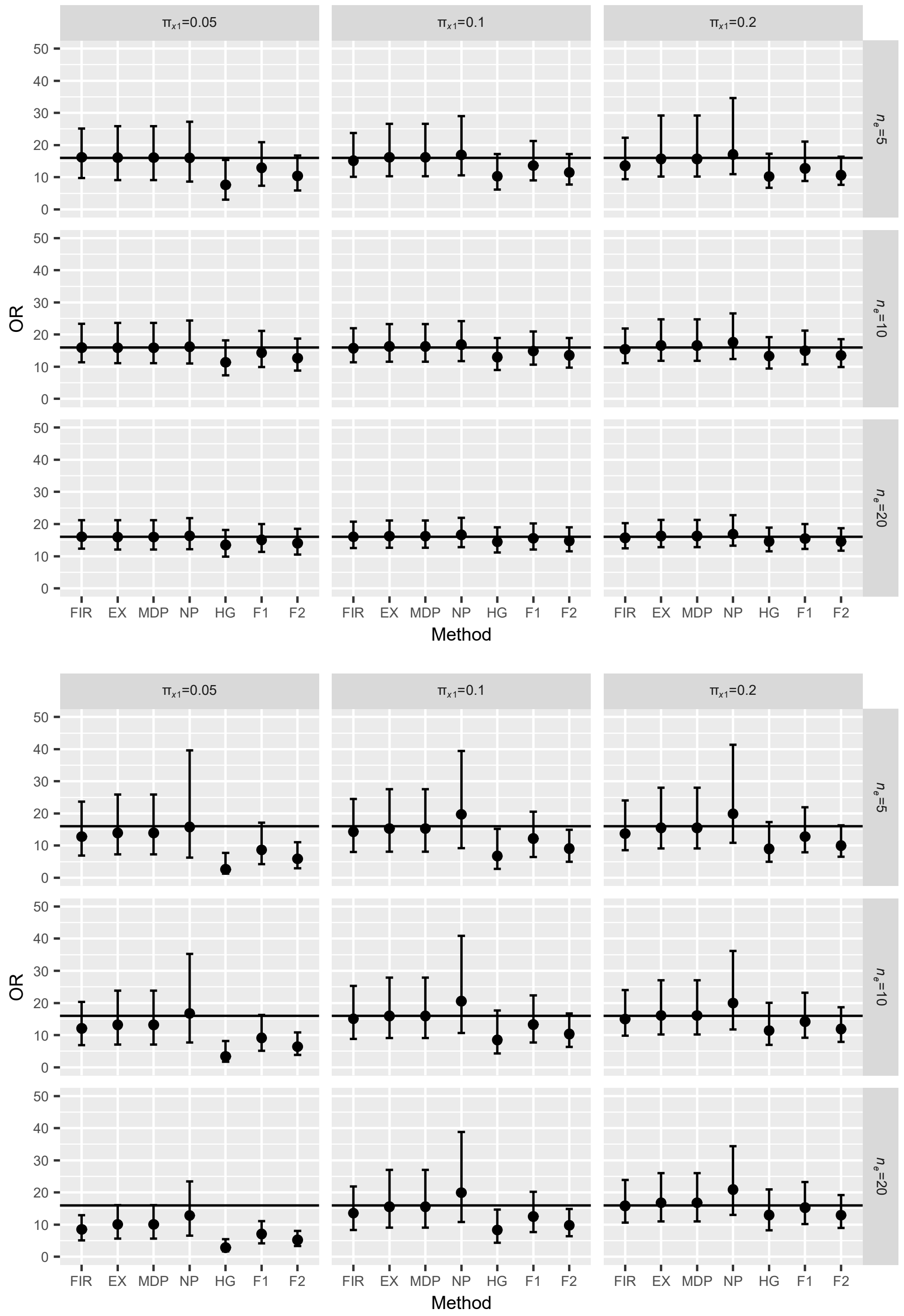

eFigure 56. Distribution of simulated OR under true OR = 16 in scenario 3 (top, n = 100; bottom, n = 1000). The square represents the median and the error bar represents quartiles 1 and 3. The solid horizontal line is the true OR value. EX: exact method; F1: Bayesian data augmentation with log $F(1, 1)$; F2: Bayesian data augmentation with log $F(2, 2)$; FIR: Firth's method; HG: Bayesian method with hyper-g prior; MDP: mid P-type exact method; NP: Bayesian method with $N(0, 100)$ prior.

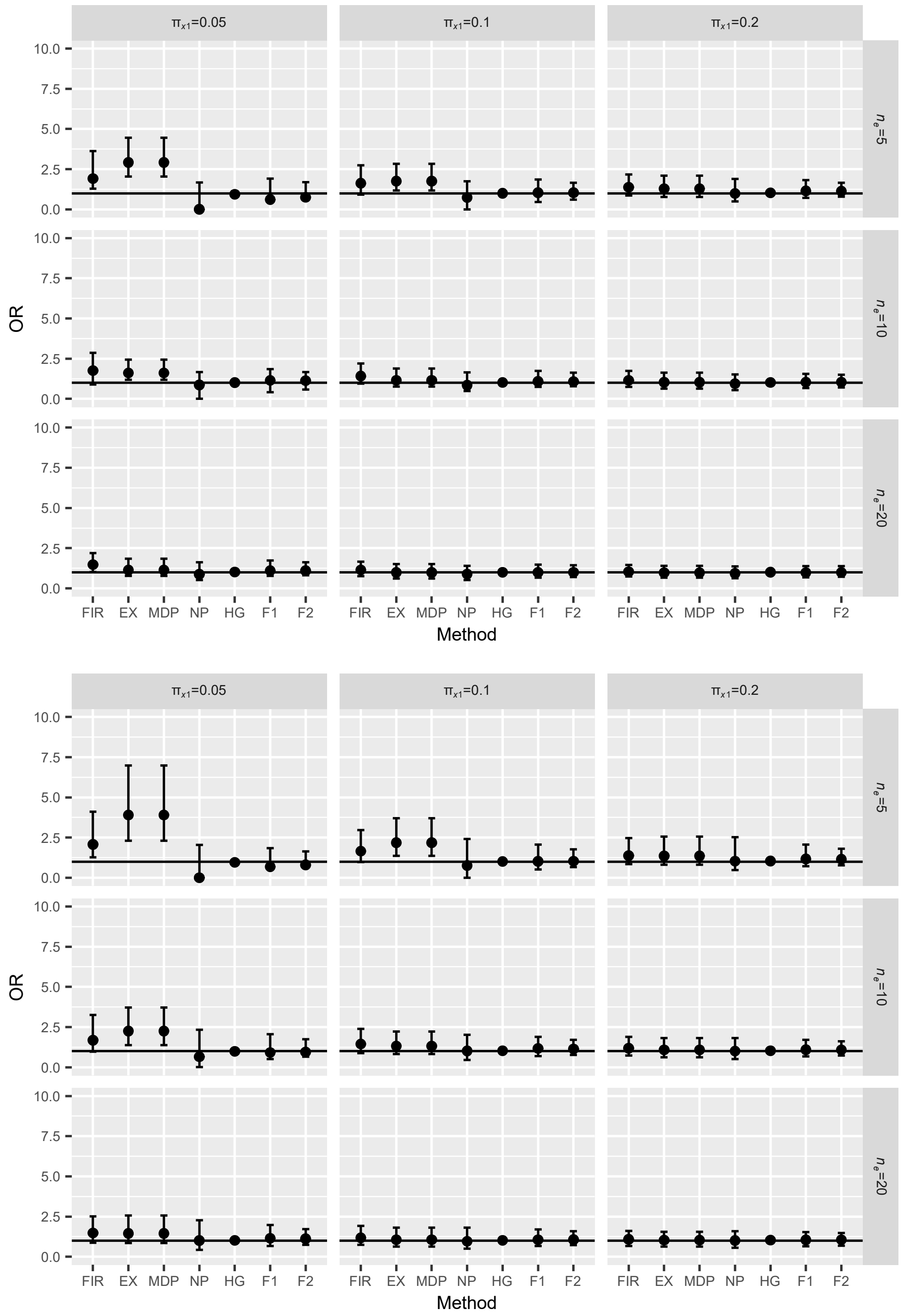

eFigure 57. Distribution of simulated OR under true OR = 1 in scenario 4 (top, n = 100; bottom, n = 1000). The square represents the median and the error bar represents quartiles 1 and 3. The solid horizontal line is the true OR value. EX: exact method; F1: Bayesian data augmentation with log $F(1, 1)$; F2: Bayesian data augmentation with log $F(2, 2)$; FIR: Firth's method; HG: Bayesian method with hyper-g prior; MDP: mid P-type exact method; NP: Bayesian method with $N(0, 100)$ prior.

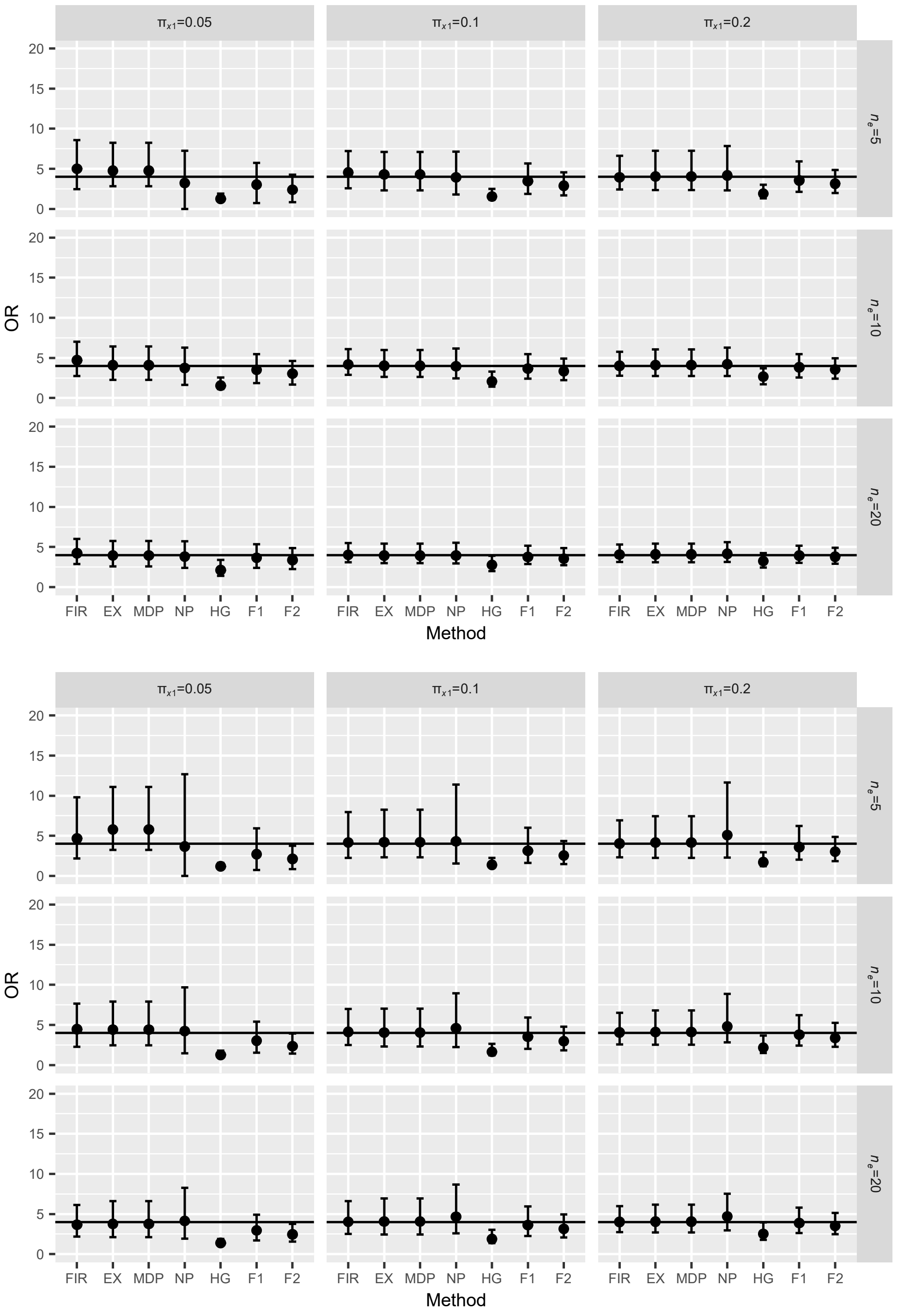

eFigure 58. Distribution of simulated OR under true OR = 4 in scenario 4 (top, n = 100; bottom, n = 1000). The square represents the median and the error bar represents quartiles 1 and 3. The solid horizontal line is the true OR value. EX: exact method; F1: Bayesian data augmentation with log $F(1, 1)$; F2: Bayesian data augmentation with log $F(2, 2)$; FIR: Firth's method; HG: Bayesian method with hyper-g prior; MDP: mid P-type exact method; NP: Bayesian method with $N(0, 100)$ prior.

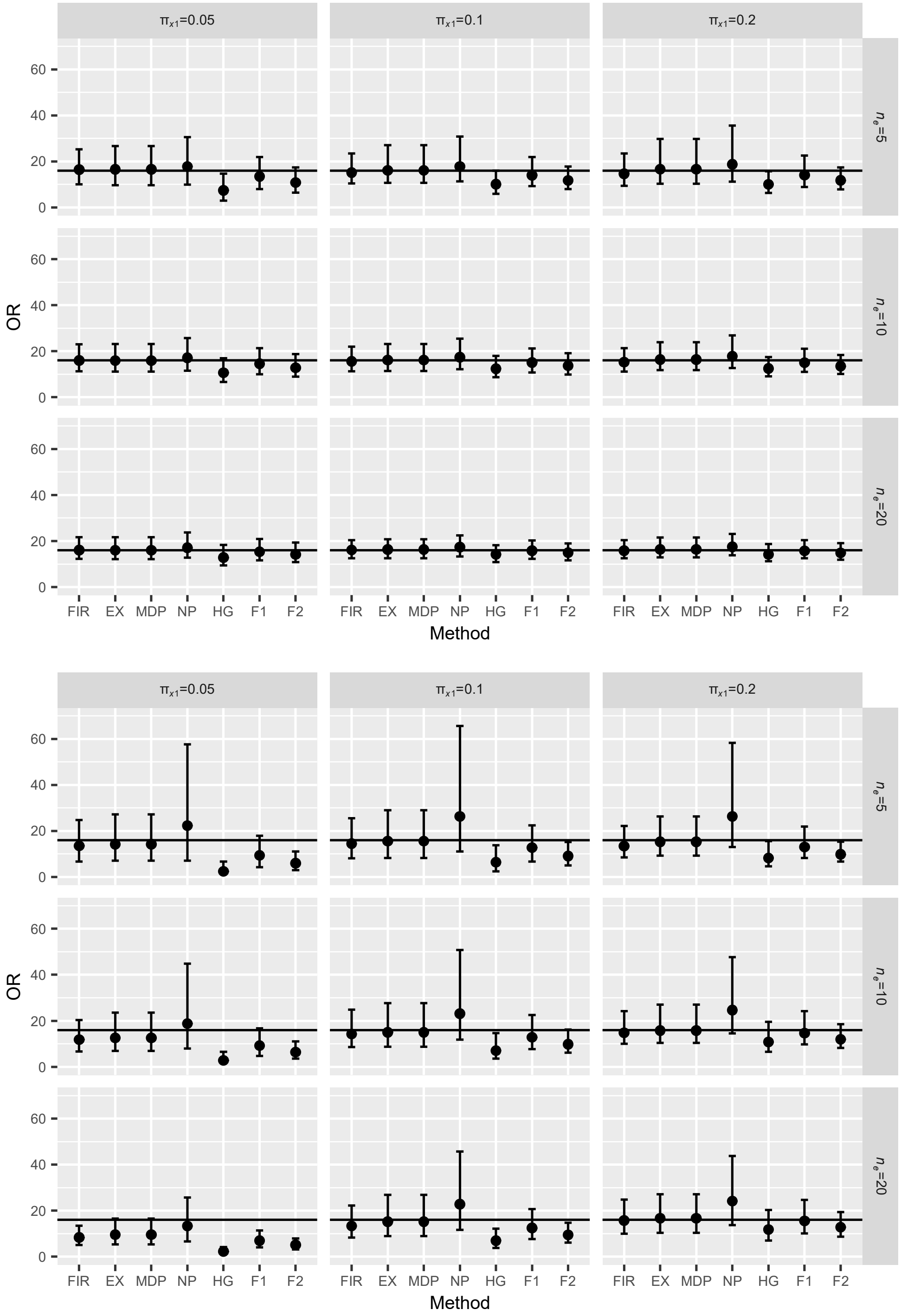

eFigure 59. Distribution of simulated OR under true OR = 16 in scenario 4 (top, n = 100; bottom, n = 1000). The square represents the median and the error bar represents quartiles 1 and 3. The solid horizontal line is the true OR value. EX: exact method; F1: Bayesian data augmentation with log $F(1, 1)$; F2: Bayesian data augmentation with log $F(2, 2)$; FIR: Firth's method; HG: Bayesian method with hyper-g prior; MDP: mid P-type exact method; NP: Bayesian method with $N(0, 100)$ prior.

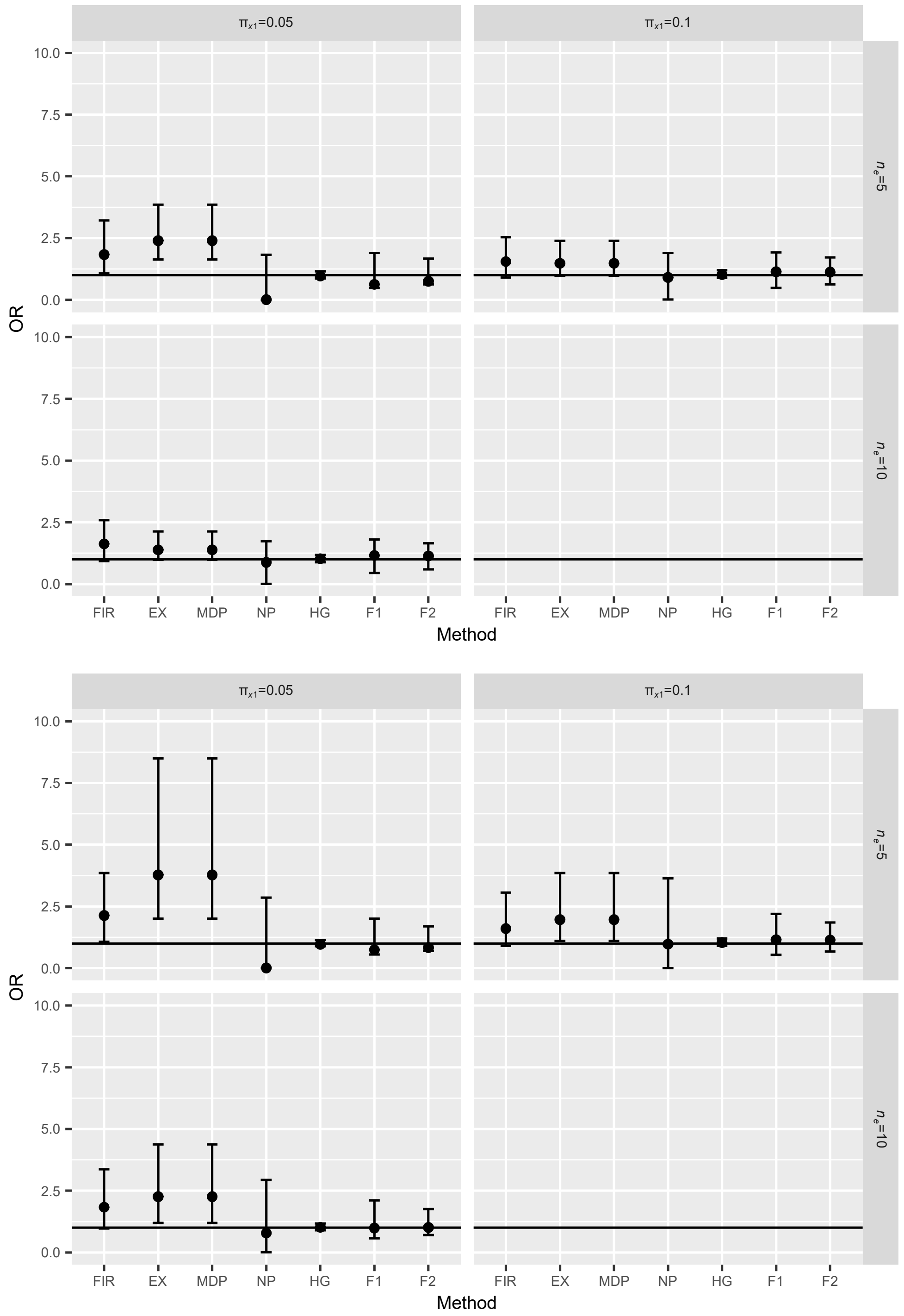

eFigure 60. Distribution of simulated OR under true OR = 1 in scenario 5 (top, n = 100; bottom, n = 1000). The square represents the median and the error bar represents quartiles 1 and 3. The solid horizontal line is the true OR value. EX: exact method; F1: Bayesian data augmentation with log $F(1, 1)$; F2: Bayesian data augmentation with log $F(2, 2)$; FIR: Firth's method; HG: Bayesian method with hyper-g prior; MDP: mid P-type exact method; NP: Bayesian method with $N(0, 100)$ prior.

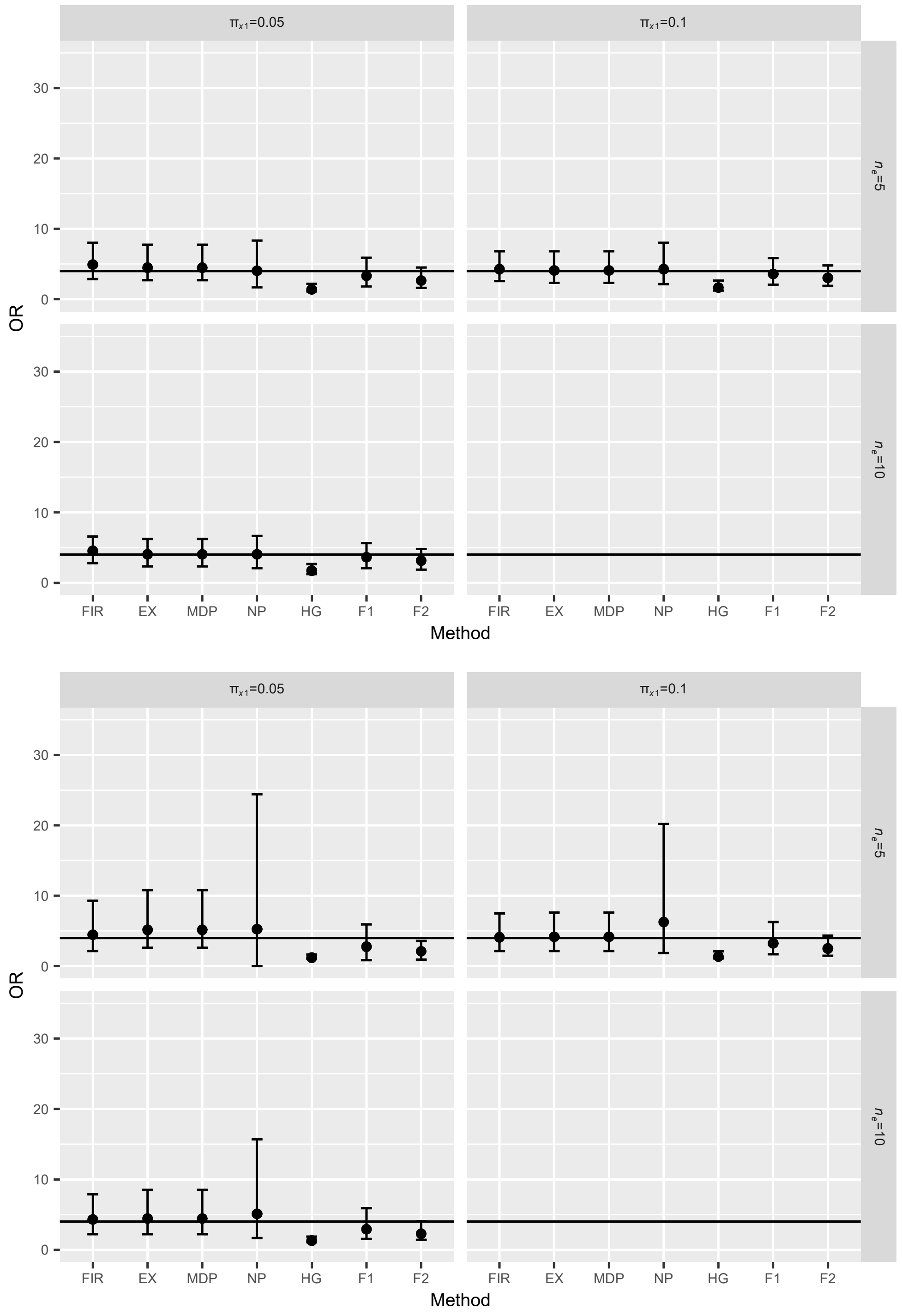

eFigure 61. Distribution of simulated OR under true OR = 4 in scenario 5 (top, n = 100; bottom, n = 1000). The square represents the median and the error bar represents quartiles 1 and 3. The solid horizontal line is the true OR value. EX: exact method; F1: Bayesian data augmentation with log $F(1, 1)$; F2: Bayesian data augmentation with log $F(2, 2)$; FIR: Firth's method; HG: Bayesian method with hyper-g prior; MDP: mid P-type exact method; NP: Bayesian method with $N(0, 100)$ prior.

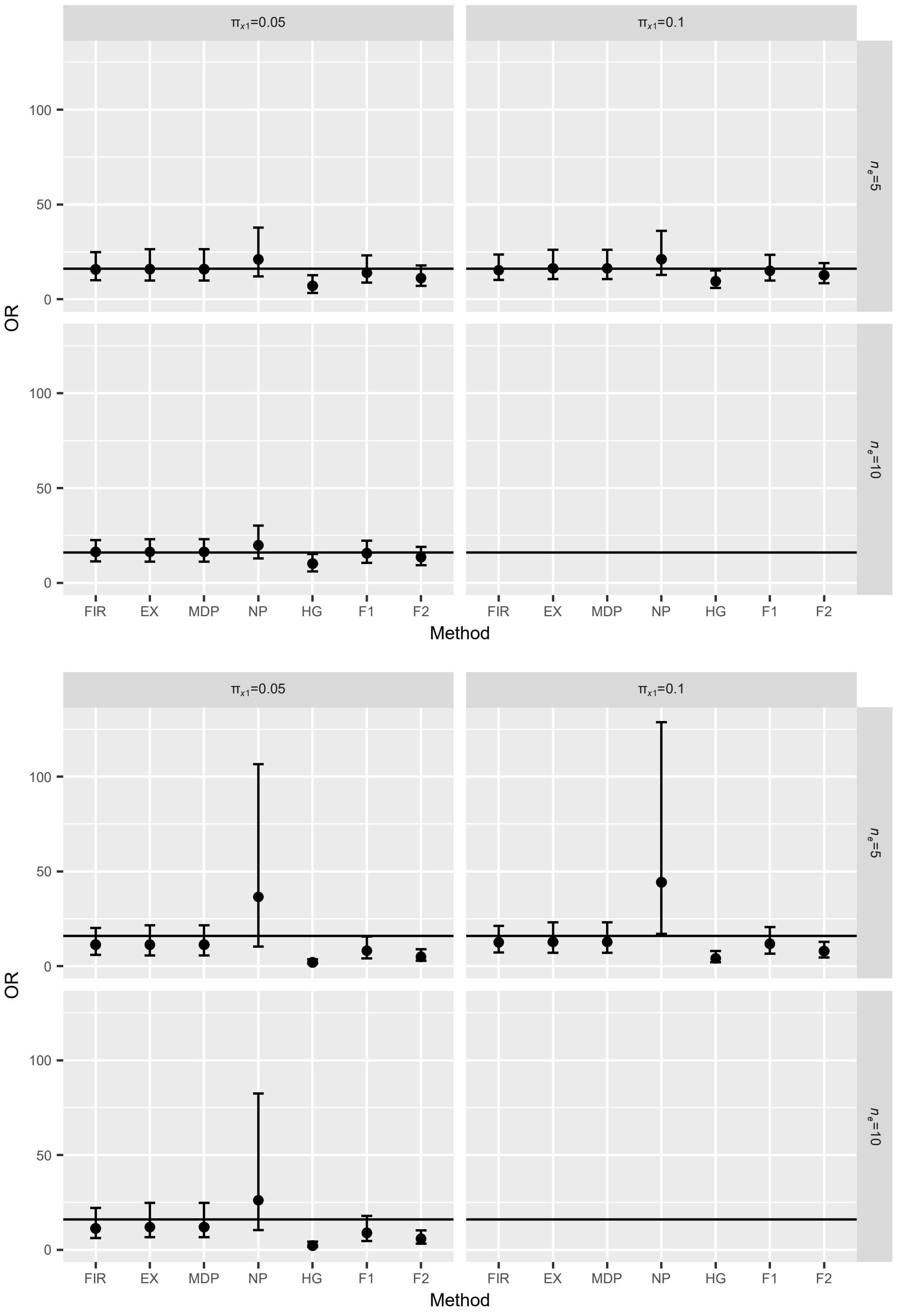

eFigure 62. Distribution of simulated OR under true OR = 16 in scenario 5 (top, n = 100; bottom, n = 1000). The square represents the median and the error bar represents quartiles 1 and 3. The solid horizontal line is the true OR value. EX: exact method; F1: Bayesian data augmentation with log $F(1, 1)$; F2: Bayesian data augmentation with log $F(2, 2)$; FIR: Firth's method; HG: Bayesian method with hyper-g prior; MDP: mid P-type exact method; NP: Bayesian method with $N(0, 100)$ prior.

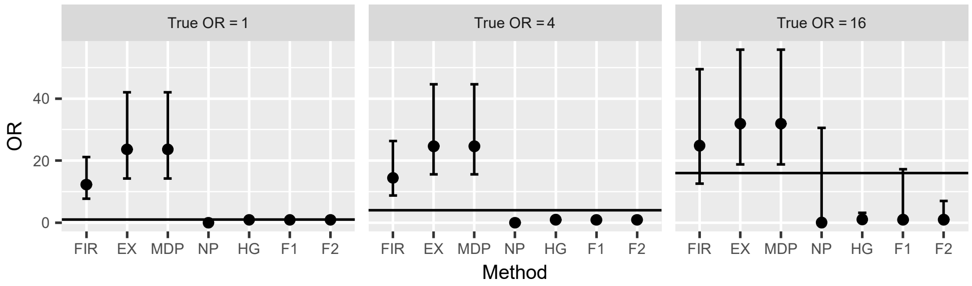

eFigure 63. Distribution of simulated OR in scenario 6. The square represents the median and the error bar represents quartiles 1 and 3. The solid horizontal line is the true OR value. EX: exact method; F1: Bayesian data augmentation with log $F(1, 1)$; F2: Bayesian data augmentation with log $F(2, 2)$; FIR: Firth's method; HG: Bayesian method with hyper-g prior; MDP: mid P-type exact method; NP: Bayesian method with $N(0, 100)$ prior.

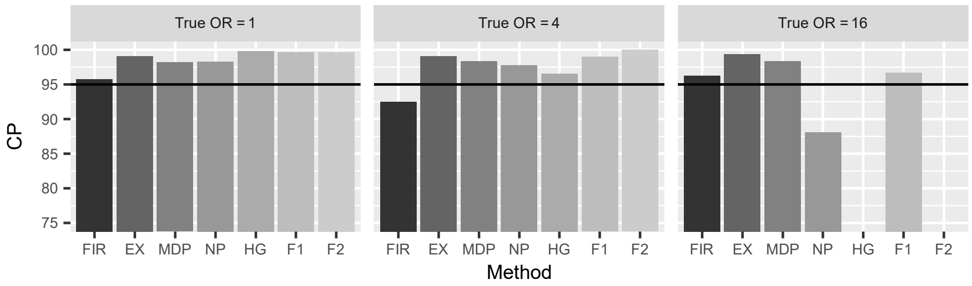

eFigure 64. 95% CI coverage probability (CP) in scenario 6. The CP is 49.2% for HG and is 71.2% for F2 when true OR = 16. EX: exact method; F1: Bayesian data augmentation with log $F(1, 1)$; F2: Bayesian data augmentation with log $F(2, 2)$; FIR: Firth's method; HG: Bayesian method with hyper-g prior; MDP: mid P-type exact method; NP: Bayesian method with $N(0, 100)$ prior.
